# Approachable Synthetic Methodologies for Second-Generation *β*-Lactamase Inhibitors: A Review

**DOI:** 10.3390/ph17091108

**Published:** 2024-08-23

**Authors:** Noor Fatima, Shehla Khalid, Nasir Rasool, Muhammad Imran, Bushra Parveen, Aqsa Kanwal, Marius Irimie, Codrut Ioan Ciurea

**Affiliations:** 1 Department of Chemistry, Government College University, Faisalabad 38000, Pakistan; noorfatima09263@gmail.com (N.F.); shehlakhalid47@gmail.com (S.K.); bushrachemgcuf@gmail.com (B.P.); aqsa6373@gmail.com (A.K.); 2 Chemistry Department, Faculty of Science, King Khalid University, P.O. Box 9004, Abha 61413, Saudi Arabia; imranchemist@gmail.com; 3 Faculty of Medicine, Transylvania University of Brasov, 500036 Brasov, Romania; marius.irimie@unitbv.ro (M.I.); codrut.ciurea@unitbv.ro (C.I.C.)

**Keywords:** *β*-lactamase inhibitors, diazabicyclooctanes (DBOs), boronate, metallo-*β*-lactamases, drug synthesis

## Abstract

Some antibiotics that are frequently employed are *β*-lactams. In light of the hydrolytic process of *β*-lactamase, found in Gram-negative bacteria, inhibitors of *β*-lactamase (BLIs) have been produced. Examples of first-generation *β*-lactamase inhibitors include sulbactam, clavulanic acid, and tazobactam. Many kinds of bacteria immune to inhibitors have appeared, and none cover all the *β*-lactamase classes. Various methods have been utilized to develop second-generation *β*-lactamase inhibitors possessing new structures and facilitate the formation of diazabicyclooctane (DBO), cyclic boronate, metallo-, and dual-nature *β*-lactamase inhibitors. This review describes numerous promising second-generation *β*-lactamase inhibitors, including vaborbactam, avibactam, and cyclic boronate serine-*β*-lactamase inhibitors. Furthermore, it covers developments and methods for synthesizing M*β*L (metallo-*β*-lactamase inhibitors), which are clinically effective, as well as the various dual-nature-based inhibitors of *β*-lactamases that have been developed. Several combinations are still only used in preclinical or clinical research, although only a few are currently used in clinics. This review comprises materials on the research progress of BLIs over the last five years. It highlights the ongoing need to produce new and unique BLIs to counter the appearance of multidrug-resistant bacteria. At present, second-generation BLIs represent an efficient and successful strategy.

## 1. Introduction

The growth and spread of antibiotic-resistant microorganisms pose severe threats to public health [1]. The global response to the challenge of bacterial resistance is being implemented urgently by governments, pharmaceutical companies, and academic institutions because of the concerning resistance levels. To address this issue, the World Health Organization developed an “international focus list of resistant-to-antibiotics bacteria”. First, various slightly to extremely drug-resistant Gram-negative opportunistic bacteria (such as *Enterobacteriaceae*, *Acinetobacter baumannii*, and *Pseudomonas aeruginosa*, which include *Klebsiella pneumonia* and *Escherichia coli*) have to be targeted. Such bacteria are unable to resist late-generation cephalosporins as well as carbapenems, which are hospital-recommended antibiotics. Such drugs are a part of the class of antibiotics of *β*-lactams, the type of antibiotics that is the most frequently prescribed to treat bacteria [2].

Many commonly used and commercially successful kinds of antibiotics are classified as *β*-lactams [3,4]. Their success can be attributed to both their wide range of activities and their good safety history. A four-membered amide cyclic functional group known as a *β*-lactam ring unites these molecules as an active group. The distinctive feature is a *β*-lactam ring consisting of four members, which is designed to inhibit the ability of penicillin-binding proteins (PBPs) to form transpeptide linkages. PBPs are enzymes essential to the formation of peptidoglycan (PG) in bacterial cell walls [5,6]. A five- or six-membered ring is added to cephalosporins, penicillins, and carbapenems, the most widely used *β*-lactam antibiotics [7].

New *β*-lactams aim to overcome resistance mechanisms and incorporate other types of bacteria. On the other hand, overuse has caused germs to become resistant. Bacteria develop resistance to *β*-lactam antibiotics through multiple mechanisms [7]. The most common resistance mechanism found in both Gram-positive and Gram-negative bacteria is the production of hydrolytic enzymes known as *β*-lactamases [8,9]. These enzymes disrupt the amide bonds in *β*-lactam antibiotics, rendering them ineffective against their intended targets [7].

Approximately 4000 *β*-lactamases have been discovered thus far, and they are divided into A, B, C, and D classes [10] according to their primary and tertiary structures and catalytic mechanisms. Serine hydrolases of A, C, and D classes exhibit a varied substrate profile, primarily comprising cephalosporins and penicillins. A small number of them, such as the KPC-type enzyme class A and OXA-48 class D, can also inactivate carbapenems. However, enzymes of class B, also known as metallo-*β*-lactamases (MBLs), because they catalyze reactions involving one or two Zn atoms, are all efficient carbapenemases that often possess several different substrates, such as penicillins and cephalosporins. Based on their structural characteristics, the three subclasses of MBLs are B1, B2, and B3 [11]. Clinically, the most important di-zinc enzymes are MBLs from subclass B1, which include VIM-, IMP-, and NDM-types. MBLs are also produced by Gram-negative bacteria on the WHO’s priority list of pathogens that can be dangerous when acquired outside of facilities. Additionally, MBL-producing isolates are becoming more and more responsible for community-acquired illnesses while originally spreading in the hospital context [12]. These enzymes are important therapeutic targets because isolates that produce MBL are widely distributed worldwide [13].

There is a lack of effective medicines to treat even mild infections caused by bacteria manufacturing *β*-lactamases because of the rise in enzyme variations. Researchers are currently focusing on finding new *β*-lactamase inhibitors (BLIs) from various sources and creating tactics that can prevent the development of drug-resistant bacteria, given the generation of novel medicines to address this issue. Consequently, to treat resistant bacterial infections, inhibitors of *β*-lactamase (BLIs), such as tazobactam, sulbactam, and clavulanate, have been used in combination with antibiotics of *β*-lactam [14]. Since then, the rise in antibiotic resistance around the world has rendered these inhibitors less effective in clinical settings. Consequently, the need for generating “second-generation” *β*-lactamase inhibitors (BLIs) to combat resistant types of virulent bacteria has increased [15,16]. Bicyclic boronic acid, metallo-*β*-lactamase inhibitors, and diazabicyclooctane (DBO) constitute the second class of non-*β*-lactam BLIs [17,18].

It is a well-known strategy to inhibit *β*-lactamases to protect *β*-lactam medicines, and various inhibitors of *β*-lactamase (such as clavulanate, tazobactam, avibactam, and vaborbactam) are now on the market [17]. They specifically target only a subset of serine-*β*-lactamases (SBLs) [19]. Only a few other drugs show promise; only two inhibitors of MBL are undergoing clinical trials (phase I trials for QPX7728 and phase III trials for taniborbactam) [18,20,21,22], and just a few other substances show potential [23,24,25]. The fact that there are several MBLs with slightly different active sites is one of the main challenges, and finding a broad-spectrum inhibitor is extremely challenging [19,20,26]. Furthermore, most inhibitors usually have a zinc-coordinating group, which raises the possibility that they could inhibit crucial human metalloenzymes [27]. The development of innovative combinations of inhibitors of *β*-lactam-*β*-lactamase, like imipenem–relebactam, ceftolozane–tazobactam, meropenem–vaborbactam, and ceftazidime–avibactam, has significantly improved the treatment arsenal against Gram-negative infections that are resistant to several drugs. Regretfully, resistance to these extremely potent medications is quickly developing in clinics [28].

In this review, the major developments toward the synthesis of *β*-lactamase inhibitors of the second generation will be discussed, along with useful approaches and techniques for developing inhibitors that work for a range of enzymes. We provide a five-year review of *β*-lactamase inhibitors, recapitulating the biological evolution and describing the development approaches that yield new *β*-lactamase inhibitors. In chemistry, second-generation beta-lactamase inhibitors are essential because they shed light on the mechanisms behind enzyme inhibition, direct the development of new drugs to address antibiotic resistance, and guide drug design efforts. Their research is critical to expanding our knowledge of antibiotic resistance and developing novel strategies to address this worldwide health issue.

## 2. Second-Generation *β*-Lactamase Inhibitors

For the past 80 years, millions of individuals have been treated for bacterial infections with antibiotics of *β*-lactam [29]. Antibiotic resistance, however, is encouraged worldwide toward the overuse or unnecessary use of antibiotics. Approximately 65% of the global antibiotic market is made up of antibiotics of *β*-lactam, which consist of penicillin, monobactams, carbapenems, and cephalosporins [30,31]. Unfortunately, the *β*-lactamase enzyme’s growth in Gram-negative bacteria and an effective mechanism of antibiotic resistance to *β*-lactams are formed by the hydrolysis of these *β*-lactam rings. Since the introduction of clavulanic acid and amoxicillin in 1981, combining an inhibitor of *β*-lactamase (BLI) with *β*-lactams has been demonstrated to be an effective method to treat this sort of resistance [14]. Class A *β*-lactamases were effectively treated with this combination therapy; but, because of the development of novel and mutant *β*-lactamases [32,33], which clavulanic acid was unable to inhibit, its effectiveness was compromised. Thus, the discovery of sulbactam and tazobactam [34] as inhibitors of class A, class C, and certain class D *β*-lactamases prompted a search for new inhibitors [35]. Because of growing resistance because of bacteria producing extended-spectrum *β*-lactamases, other well-known BLIs, like tazobactam and sulbactam, are no longer clinically effective [36]. Hence, the need for developing next-generation BLIs to combat resistant types of pathogenic bacteria has increased [15,16]. The new second-generation class of inhibitors of non-*β*-lactam beta-lactamase is divided into diazabicyclooctane (DBO), bicyclic boronic acid, metallo-, and dual-nature-based *β*-lactamase inhibitors.

## 3. 1,6-Diazabicyclo [3,2,1] Octanes

A class of inhibitors known as diazabicyclooctanes (DBOs) uses a strained-urea moiety as the warhead to bind specifically to the active serine residue in the SBL’s active site. One useful method for drug design is to derive new DBOs with increased activity by modifying chemicals using exhaustive structure–activity interaction assays [37]. Newly developed molecules have led to the expansion of the DBO class, typically because of alterations in the C-2 linkages. Gao et al. substituted the C-2 position of the DBO scaffold with several amidine derivatives to form water-soluble compounds. To produce the final product required for synthesizing BLIs utilizing DBO, the majority of the new chemical compounds, including active ingredients of pharmaceuticals (APIs), need to undergo an amide linkage [38]. A recent study indicates that DBO-based lactamase inhibition holds the potential for the development of new drugs. Several researchers and drug companies are working to synthesize new derivatives of DBO that can inhibit any kind of *β*-lactamase [39].

### 3.1. Avibactam Derivatives

**Scheme 1:**
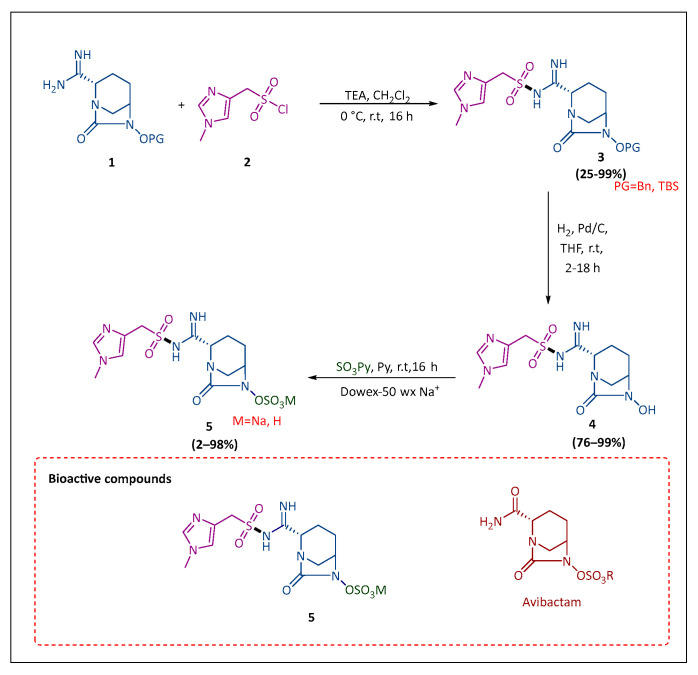
Synthesis of novel avibactam derivatives via sulfonyl amidine.

Clinically approved as an inhibitor of *β*-lactamase that is not dependent on *β*-lactam is avibactam. The diazabicyclooctane (DBO) family of antibiotics has two members: avibactam [40], which is the first member to enter clinical use with ceftazidime as the combination antibiotic, and relebactam, which was just approved when combined with imipenem [41]. Ji et al. effectively synthesized the new avibactam derivatives with sulfonyl amidine moieties at the diazabicyclooctane ring’s C-2 position. They evaluated the compound’s bacterial strains that are resistant to the in vitro effectiveness of antibacterial agents that expressed varied *β*-lactamase activity. The compound (**1**) was reacted with sulfonyl chlorides or anhydride (**2**) to form compound **3**. These compounds were subsequently debenzylated using Pd/C to produce the respective OH derivative (**4**). The final compound (**5**) was subsequently produced by the reaction between the hydroxy compound (**4**) and the SO_3_–pyridine complex [42,43]. When compound (**5**) was compared to the control inhibitor, relebactam, it showed the most effectiveness against bacterial strains. Compound (**5**) (<0.0312) might be a promising candidate for further research (Scheme 1) [39].

**Scheme 2:**
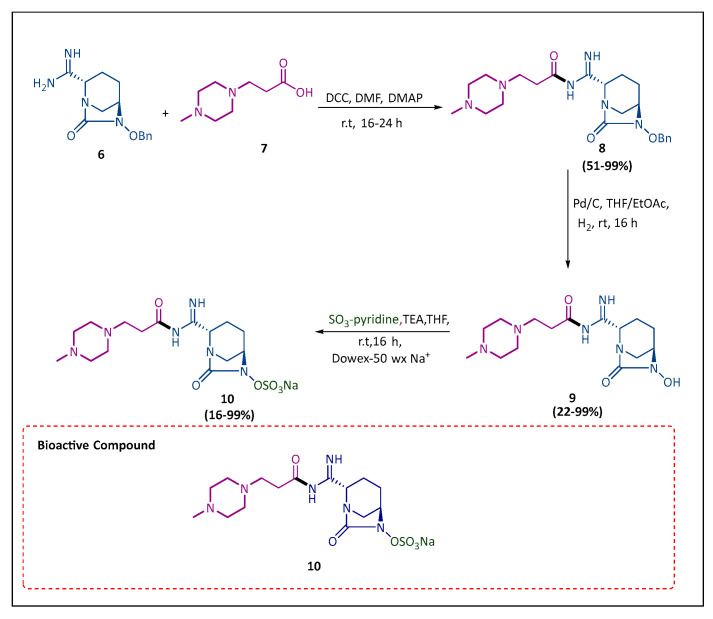
Synthesis of new avibactam derivative with amidine.

A non-*β*-lactam-based antibiotic of the second generation is avibactam. A substitute for the *β*-lactam ring was identified: the diazabicyclooctane (DBO) [44] ring [17]. Several inhibitors based on non-*β*-lactams, class A, C, and certain class D *β*-lactamases [35], suppress these classes of *β*-lactamases, like avibactam, relebactam, sulbactam, and tazobactam [34]. Gao et al. successfully synthesized a series of amidine-substituted avibactam derivatives in moderate to good yields. In vitro, tests were performed to assess the synthetic compound’s antibacterial activity and combined with an antibiotic with synergistic effects. These substances were evaluated both by the compounds and in conjunction with the widely used antibiotic meropenem against bacterial strains. Compound (**6**) and compound (**7**) form compound (**8**) by coupling reagents DCC/DMAP in the presence of DMF solvent, whereas DMAP was used as a base. The compound (**9**) was produced via the hydrogenation catalyzed by Pd of compound (**8**) in THF or EtOAc. The compound (**9**) then reacted with SO_3_ to obtain the sulfonic-acid derivative (**10**). Compound (**10**) showed activities against bacterial strains that were either equivalent to or better than those of avibactam, and it showed to be the more potent compound among the others in combating bacterial species, with MICs between 0.29 and 2.34 μM. Compound (**10**) might be a promising lead for further in vivo research and clinical investigations (Scheme 2) [45].

**Scheme 3:**
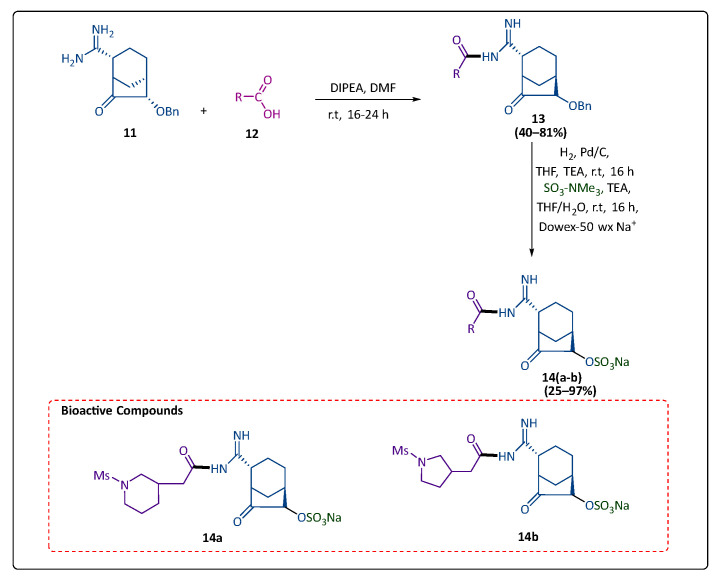
Synthesis of novel substituted amidine analogs.

Avibactam, a non-βLI made from a DBO scaffold, is an efficient inhibitor against *Pseudomonas aeruginosa* [46,47,48], extended-spectrum *β*-lactamases (ESBLs), carbapenemases, and multidrug-resistant *Enterobacteriaceae* (MDR) [43] when combined with ceftazidime [40]. Sun et al. synthesized new avibactam derivatives in which a substituted amidine group replacing the amide moiety were obtained in many steps. The most important step is to convert the 2-positioned cyano group of the 1,6-diazabicyclo [3,2,1] octane structure to an amidine group. The new compound’s synergistic antibacterial action in conjunction with meropenem suggests that these derivatives have an agonistic impact on *β*-lactamases. The organic acids at NH_2_ were combined to form compound (**11**), which then reacted with compound (**12**) to produce a significant compound (**13**) [46], which underwent additional debenzylation and converted to the corresponding compounds (**14(a,b)**) in good yields. Compared to MER alone (MIC, 4 mg dm^−3^), ‘’**14(a**,**b)’’** had the strongest activities in contrast to the majority of the tested strains of bacteria, with an MIC value of less than 0.125 mg dm^−3^. Compounds ‘’**14(a**,**b)’’** exhibited the highest efficacy, with an MIC value of less than 0.125 mg dm^−3^ against *P. aeruginosa* 9027 and were also highly active against *A. baumannii* 19,606 (MIC, 0.5 mg dm^−3^) and an *A. baumannii* clinical isolate (MIC, 0.25 mg dm^−3^) (Scheme 3) [43].

**Scheme 4:**
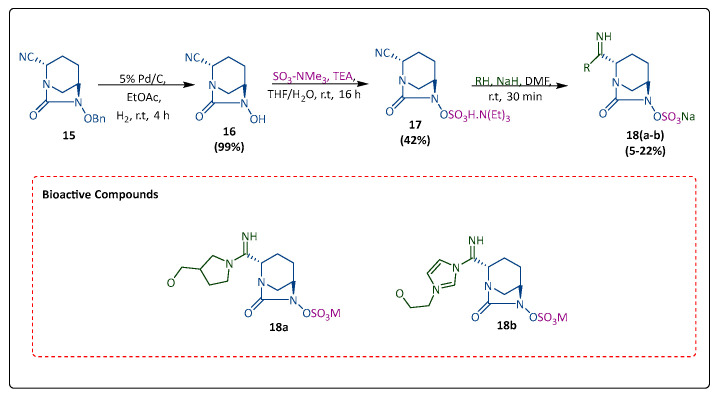
Synthesis of new imidate derivatives.

The lactamase inhibition capacity of inhibitors of *β*-lactamases (BLIs) [9] is used in multidrug therapy [49,50] to regenerate the antibacterial effectiveness of conventional *β*-lactam (BL) medications that have been impaired [17]. Since its potential to connect with serine-*β*-lactamases was discovered [3], the diazabicyclooctane (DBO) ring has been the focus of research toward the identification of second-generation BLIs. Zhai et al. synthesized several new imidate derivatives to investigate the potential *β*-lactamase inhibitory action of avibactam (diazabicyclooctane derivative). The compound (**18**) was synthesized by a series of processes, involving the hydroxy intermediate’s sulfonation, late-stage bicyclic ring alteration at C-2, and deprotection of benzyl in diazabicyclooctane’s cyano derivative. In the presence of EtOAc, compound (**15**) reacted with H_2_ to generate compound (**16**) [37]. The produced hydroxy derivative (**16**) was utilized for the following stage without being purified because it was unstable at room temperature. Subsequently, molecule **16** was sulfonated by reacting with the SO_3_–TEA complex to generate the triethylamine complex, which is the important common intermediate (**17**). The final compound (**18(a,b)**) was then formed in a good yield by reacting compound (**17**) with the suitable alkoxides, modifying it at the C-2 position. Despite being aliphatic or aromatic, nitrogen-containing heterocyclic rings also showed equal importance, as demonstrated by compounds **‘’18(a,b)’’**, demonstrating the involvement of nitrogen atoms in lactamase inhibition. (Scheme 4) [51].

**Scheme 5:**
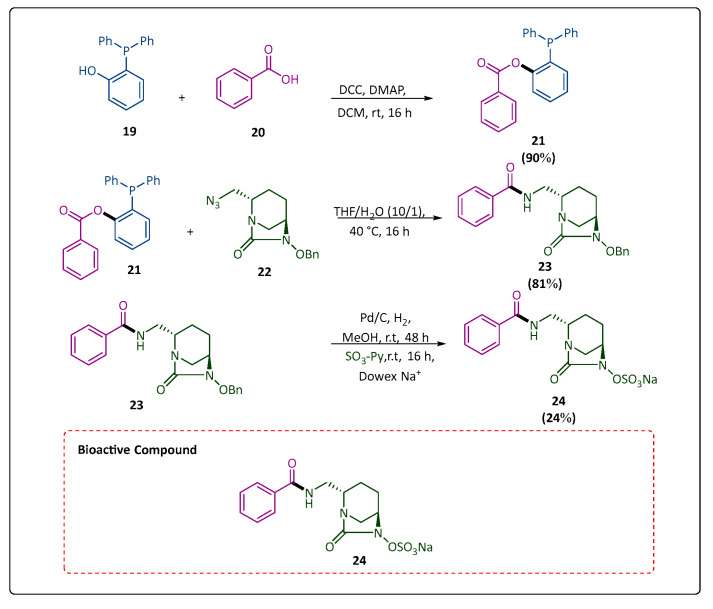
Synthesis of amide derivatives.

Two generations of inhibitors, based on the diazabicyclooctane scaffold (DBO; e.g., avibactam in 2015) [52,53] and a *β*-lactam (clavulanate in 1974) [54], have been successfully produced. The effectiveness of both kinds of inhibitors has been hindered, nonetheless, by the emergence of medical strains that become resistant to drug combinations after obtaining mutations containing ineffectively limited *β*-lactamases. This includes the acquisition of mutations during treatment as well as horizontal gene transfers [55]. Using a diazabicyclooctane (DBO) scaffold, Bouchat et al. studied the use of traceless Staudinger ligation to functionalize the C-2 position of inhibitors of second-generation *β*-lactamases. This was accomplished by creating a diazabicyclooctane (DBO) scaffold by ligating phosphine phenol esters to an azido-containing precursor. The phosphine phenol ester (**21**) formed when compound (**19**) and compound (**20**) were coupled with DCC and DMAP in DCM. Compound (**21**) and compound (**22**) were synthesized, and then both compounds underwent traceless Staudinger ligation in a mixture of (10/1) THF and H_2_O at 40 °C for 16 h to form the product compound (**23**). Compound (**23**) underwent a three-step process, which involved sulfurization with a sulfur trioxide–pyridine complex, hydrogenolysis in the presence of Pd/C to debenzylate, and elution using DOWEX Na^+^ to give compound (**24**) in a good yield. Compound **24** showed better inhibition of two of them (CTX-M-15 and OXA-48). Inhibitors were effective against these enzymes, especially OXA48, which is weakly inhibited by current DBO, and may be obtained by having access to the novel form of the amide linkage found in (**24**) (Scheme 5) [56].

### 3.2. IID572

**Scheme 6:**
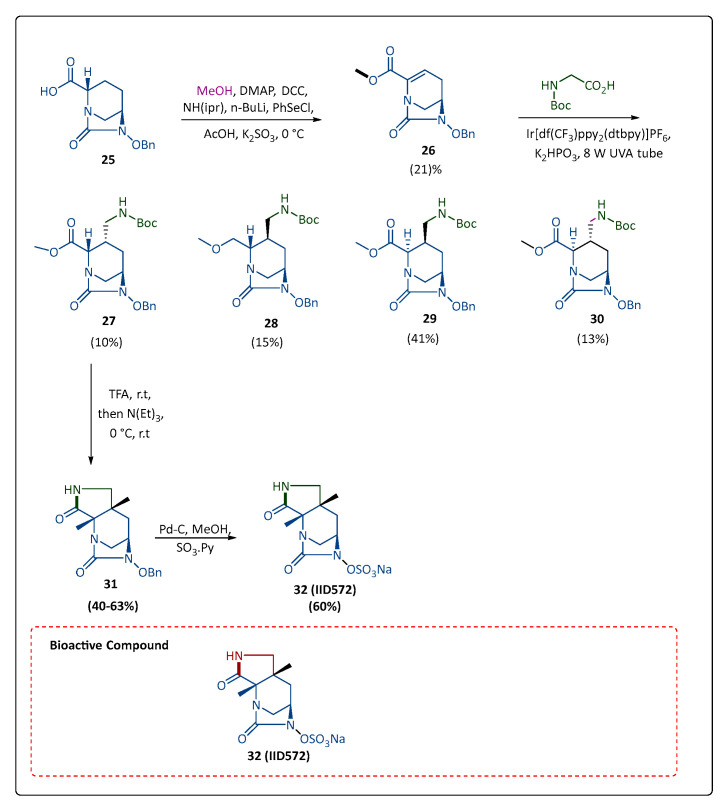
Synthesis of IID572 by photo-redox coupling.

When applied to clinically isolated bacteria that are resistant to piperacillin and tazobactam, **IID572**, a new broad-spectrum BLI related to the diazabicyclooctane (DBO) family, can reinstate the antibacterial activity of piperacillin. Reck et al. synthesized compound **25** using photo-redox coupling and is designated as IID572, which is distinct from other DBOs because of its extensive suppression of *β*-lactamases and absence of an inherent antibacterial activity. When compound (**25**) is exposed to methanol at room temperature, it is transformed to compound (**26**), which is then combined with Boc-Gly-OH to generate compounds (**27–30**) through radical Michael additions, which result in the amino-substituted diastereomeric methylene derivatives. Deprotection of the amino group in compound (**27**) with TFA led to cyclization, which produced compound (**31**) following neutralization, which is hydrogenated to remove the benzyl group. Then, compound (**31**) reacted with SO_3_/pyridine to give compound **32** (IID572) in a good yield. This compound (**32**) exhibits broad-spectrum inhibition of *β*-lactamases and efficiently shields piperacillin from degradation by drug-resistant *Enterobacteriaceae* that express serine-*β*-lactamase, hence restoring efficacy against the majority of the strains of TZP-resistant bacteria that were examined (Scheme 6) [57].

**Scheme 7:**
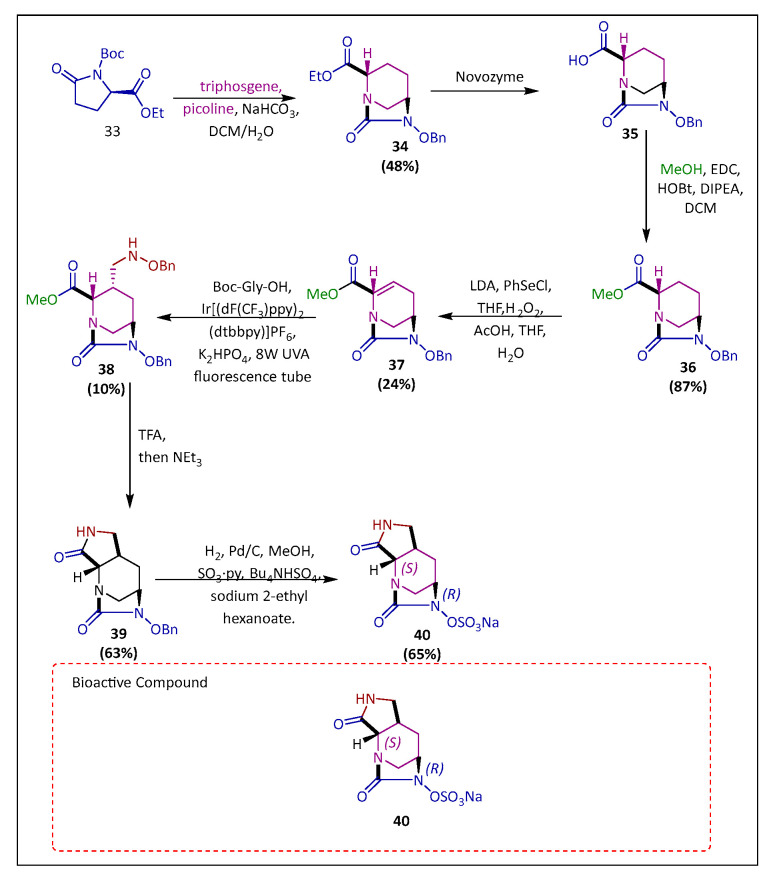
Synthesis of *β*-lactamase inhibitor IID572.

A novel *β*-lactamase inhibitor, IID572 maybe the best in its class because of a late-stage functionalization approach. According to the diazabicyclooctane (DBO) class, IID572 is possibly the best *β*-lactamase inhibitor available, as it has been demonstrated to be a potent, broad-based inhibitor without any antibacterial action. For the new *β*-lactamase inhibitor IID572, Furegati et al. proposed a synthesis technique to ensure material supply for preclinical toxicity research. Compound (**33**) reacted with triphosgene and picoline in DCM/H_2_O to form compound (**34**), which further reacted with novonzyme to form compound (**35**) [58]. Compound (**35**) reacted with methanol to form compound (**36**). A photochemical Giese-type radical addition to the Michael acceptor (**36**) was a crucial step in our LSF method, yielding a mixture of (**37**) [59]. Compound (**38**) was produced by reacting with Boc-Gly-OH. Compound (**39**) was generated spontaneously under basic conditions by the primary amine, following the separating of the required isomer (**38**) and deprotection with TFA. Compound (**39**) reacted with SO_3_·py and sodium 2-ethyl hexanoate to give compound 40. Diazabicyclooctane (DBO)-class new *β*-lactamase inhibitors showed (**40**) to be an effective, potentially among the best, wide-spectrum *β*-lactamase inhibitor (Scheme 7) [60].

### 3.3. 2-Sulfinyl-Diazabicyclooctane Derivatives

**Scheme 8:**
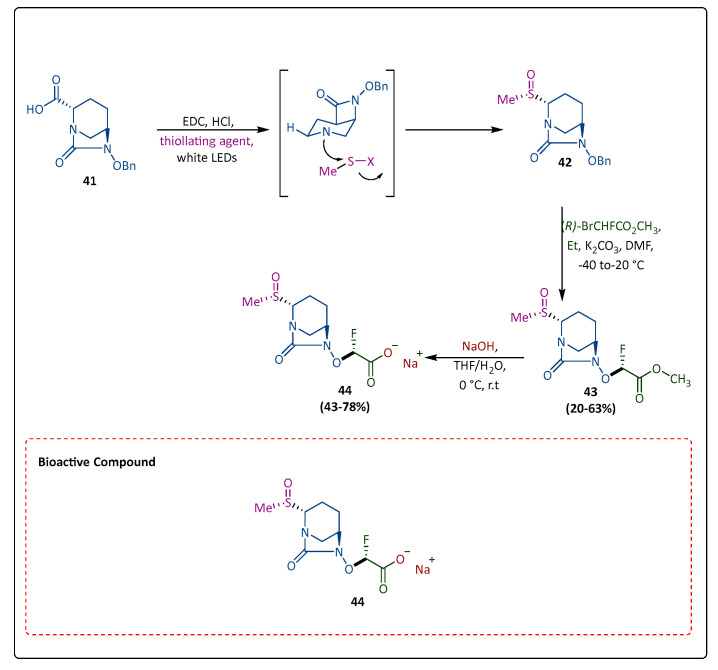
Synthesis of 2-sulfinyl-diazabicyclooctane derivative.

A well-researched therapeutic approach for bacterial infections caused by *β*-lactam-resistant Gram-negative bacteria is the co-administration of *β*-lactam and *β*-lactamase inhibitors (BLIs). For orally active BLIs, clavulanic acid and sulbactam are the only two options available. Fijiu et al. discovered that the 2-sulfinyl-diazabicyclooctane derivative inhibits oral lactamase production in infections caused by *Enterobacterales* that produce serine lactamases. Compound (**41**) reacted with a thiolating agent to form compound (**42**). Following alkylation with compound (**42**) produced by the analogous optical resolution [41,61], compound (**42**) reacted with (*R*)-BrCHFCO_2_R^2^ in the presence of DMF [62,63] to yield compound (**43**). Targeted compound (**44**) was obtained through the NaOH hydrolysis of the Bzh (benzhydryl) and ethyl ester. Compound (**44**) reinstated the antibacterial properties of ceftibuten (CTB), an oral third-generation cephalosporin, against a range of strains generating serine-*β*-lactamase, including *Enterobacteriaceae* that are unable to resist carbapenems. In conjunction with CTB, it demonstrates in vivo effectiveness and can be taken orally through ester prodrug modification (Scheme 8) [64].

### 3.4. Triazole Derivatives

**Scheme 9:**
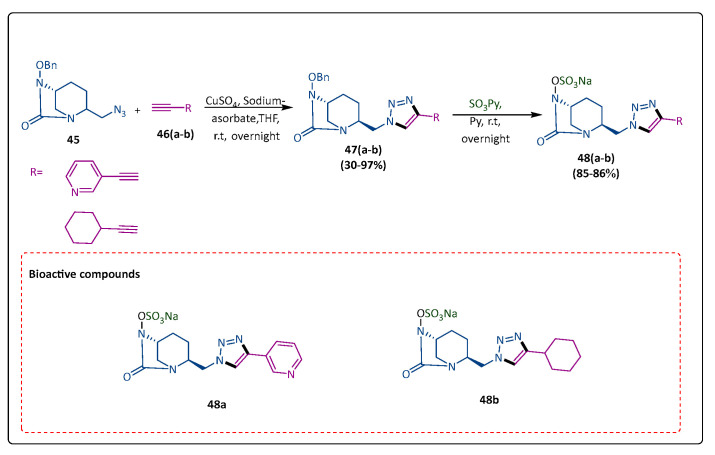
Synthesis of triazole-containing DBOs.

The diazabicyclooctane (DBO) design of inhibitors of second-generation *β*-lactamases makes *β*-lactams effective again against pathogenic bacteria, including those that produce enzymes of classes A, C, and D that are resistant to the *β*-lactam ring of inhibitors of the first generation. Bouchet et al. reported a synthetic pathway comprising triazole-containing DBOs that has been optimized. Furthermore, the biological evaluation of many compounds’ capacities to inhibit *β*-lactamases, common enzymes present in harmful Gram-negative bacteria, has been conducted. Compound (**45**) reacted with alkynes (**46(a,b)**) under CuAAC conditions, employing CuSO_4_ and the sodium salt of ascorbic acid in THF/H_2_O, during an overnight period at room temperature. Using the sulfur trioxide–pyridine complex, hydrogenolysis is used to deprotect the benzyl group of compounds (**47**). Filtration, residual dissolution in ethanol, and elution through DOWEX Na^+^ maximize the purity of the final product (**48(a,b)**). The most potent DBOs ‘’**48(a**,**b**)’’ in vitro were also some of the best substances for boosting *β*-lactam activity against *E. Coli* strains that produced CTX-M-15 or KPC-2 (Scheme 9) [65].

## 4. Boron-Based *β*-Lactamase Inhibitors

A new substrate for the generation of *β*-lactamase inhibitors is represented by boronate-based compounds. Out of all the *β*-lactamase inhibitors that have been produced thus far, boron-based inhibitors of *β*-lactamases are probably the most promising class that is currently on the market for the treatment of bacteria that are resistant to these enzymes. The finding that boronic acids can inhibit serine proteases led to the formation of these compounds [66]. Zhou et al. reported the design and synthesis of new transition-state derivatives of boronic acid with a 1,4-substituted 1,2,3-triazole scaffold. These scaffolds show promise in the generation of inhibitors of the KPC-2 development that can restore sensitivity to the antibiotic cefotaxime [67].

### 4.1. Vaborbactam Derivatives

**Scheme 10:**
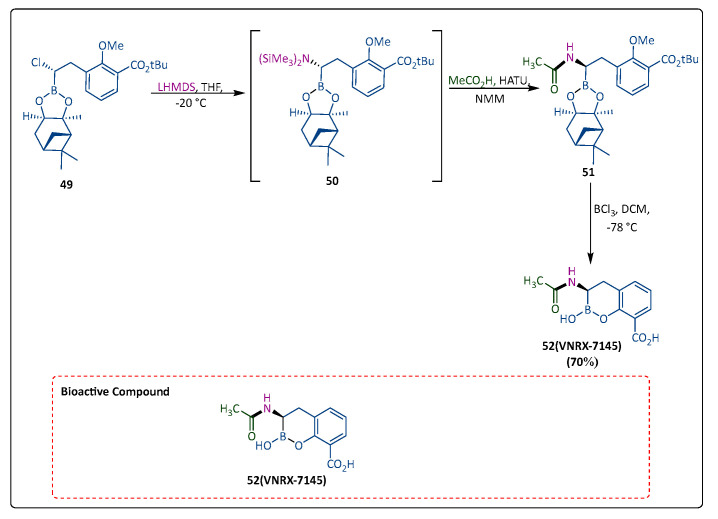
Synthesis of VNRX-7145.

The boron-based covalent and slowly reversible vaborbactam inhibitors [68] taniborbactam (formerly VNRX-5133) [22,69] and QPX7728 [70] efficiently block enzymes of classes A and C and enzymes of classes A, B, C, and D (taniborbactam and QPX7728). Nonetheless, IV products were the main focus of the pharmaceutical industry’s and academic groups’ early research on boronic-acid-based BLIs [22,68,69,70,71,72]. Trout et al. designed the lead optimization, prodrug pharmacokinetics (PKs), and microbiological profiling that resulted in the identification of VNRX-7145 (VNRX-5236; etzadroxil) [71]. At a temperature below 20 °C, compound (**49**) reacted with lithium *bis*(trimethylsilyl)amide to produce compound (**50**). Then, compound (**50**) reacted with methanoic acid to produce (**51**). To obtain the boronate (**52**), the amides (**51**) are cyclized and deprotected. Rats, dogs, and monkeys all showed good oral bioavailability. The prodrug ester hydrolyzes to liberate the active BLI (**52**), as some species have demonstrated. Compound (**52**) restored ceftibuten’s action in a study using mice exhibiting a urinary tract infection (UTI) produced by strains of *K. pneumonia* and *E. coli* that produce KPC carbapenemase and ESBL (Scheme 10) [71].

### 4.2. 1,2,3-Triazole Derivatives

**Scheme 11:**
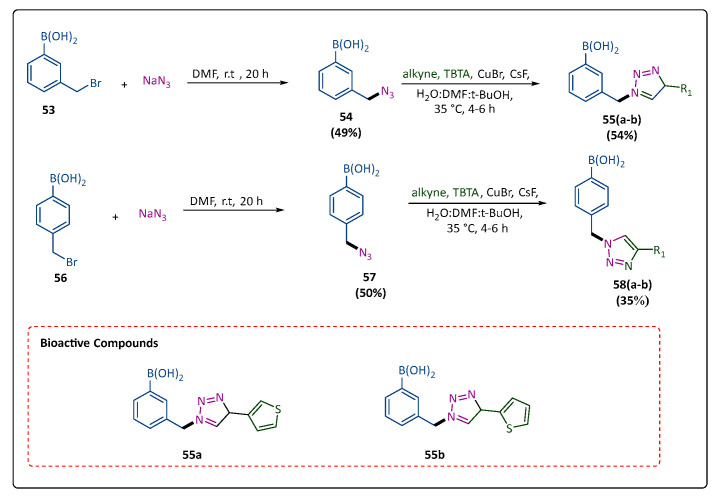
Synthesis of novel transition-state analogs of boronic acid.

One of the biggest issues facing medical science today is the quickening rise in antibiotic resistance. In most cases, the development of enzymes of *β*-lactamases, including KPC-2, is the reason behind antibiotic resistance to *β*-lactams. Therefore, it is critical to find new inhibitors of KPC-2 and similar enzymes. Using the well-known 3-nitrophenyl boronic acid inhibitor, Zhou et al. designed and synthesized novel transition-state derivatives of boronic acid through the use of a 1,4-substituted 1,2,3-triazole linker. The development of KPC-2-development inhibitors that can once again become susceptible to the antibiotic cefotaxime appears to be promising with these scaffolds. The compound (**53**) reacted with sodium azide in anhydrous DMF to produce compound (**54**), which subsequently combined with TBTA to produce (**55(a,b))**. The compound (**56**) also reacted with sodium azide to produce compound (**57**), which was subsequently treated with TBTA to form compounds (**58(a,b))**. The meta-analogs outperform the corresponding para-analogs according to susceptibility tests and in silico docking results. The results indicated that substituents that have smaller sizes at the triazole ring’s 4-position are more likely to be allowed. This is probably because of better packing against the hydrophobic loop that lies between the a10 and a11 helices. However, compared to both 3-NPBA (3-nitrophenylboronic acid) and the thiophene derivatives ‘’**55(a,b)’’**, it was shown that the zone of the inhibition increased statistically significantly (Scheme 11) [67].

**Scheme 12:**
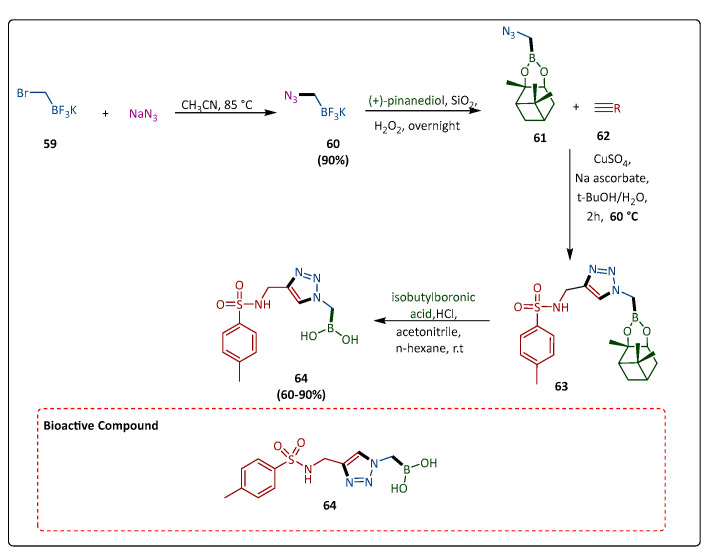
Synthesis of triazolylmethaneboronate.

Boric acid transition-state inhibitors, or BATSIs, are reversible covalent inhibitors of serine-*β*-lactamases. One well-known and tested synthesis technique is the high potency and selectivity of certain BATSIs with an amide side chain that resembles the amide side link of *β*-lactams. Caselli et al. developed bioisostere triazole, which is used in place of the amide group in a new class of BATSIs. These compounds were intended to function as molecular tools. A series of α-triazolylmethaneboronic acids were synthesized and compared with the therapeutically useful cephalosporinase ADC-7, which is generated from Acinetobacter. Compound (**59**) reacted with sodium azide in acetonitrile at 85 °C to produce compound (**60**), with a 90% yield, which was then converted to compound (**61**) by the reaction of pinanediol, with a 90% yield [73,74,75]. Compound (**61**) reacted with compound (**62**) to form compound (**63**) [76,77]. The compounds that were substituted differentially at the R_1_ group were effortlessly extracted and utilized as such for the subsequent phase. The final compounds (**64**) were obtained by transesterification using isobutyl boronic acid in a biphasic system of acetonitrile/n-hexane, which allowed for the final deprotection of compounds (**63**). Compound (**64**) (K_i_ 90 nM), the best inhibitor found, posits a tolyl group next to Arg340, resulting in advantageous cation–π interactions. Compound (**64**) turned out to be the best one: the lower the MIC, the stronger the compound’s affinity in both microbiological and kinetic profiles (Scheme 12) [78].

## 5. Metallo-*β*-Lactamase Inhibitors

MBLs are *β*-lactamases that catalyze processes with the help of metal ions. There are currently no MBL inhibitors that can be utilized in clinical trials, despite the well-known strategy for combining *β*-lactamase inhibitors to restore the efficacy of *β*-lactams. Consequently, the production of inhibitors of MBLs is essential. Because of their rising global frequency in pertinent opportunistic Gram-negative bacteria, the public’s health is severely threatened by metallo-*β*-lactamases (MBLs). The most valuable and often used antibiotics of *β*-lactams, such as carbapenems and oxyiminocephalosporins (ceftriaxone and ceftazidime), are effectively rendered as inactive by MBLs. Muhammad et al. used a three-stage technique, with the Banert cascade reaction serving as the crucial step, to prepare several NH-1,2,3-triazoles. Biochemical experiments were used to assess the inhibitor’s characteristics against the MBLs GIM-1, NDM-1, and VIM-2 [79]. Numerous substances, including carboxylic acids and molecules containing thiols, that can align potential MBL inhibitors are associated with the zinc ions at the active site of the enzyme [80].

### 5.1. Mercapto Derivatives

**Scheme 13:**
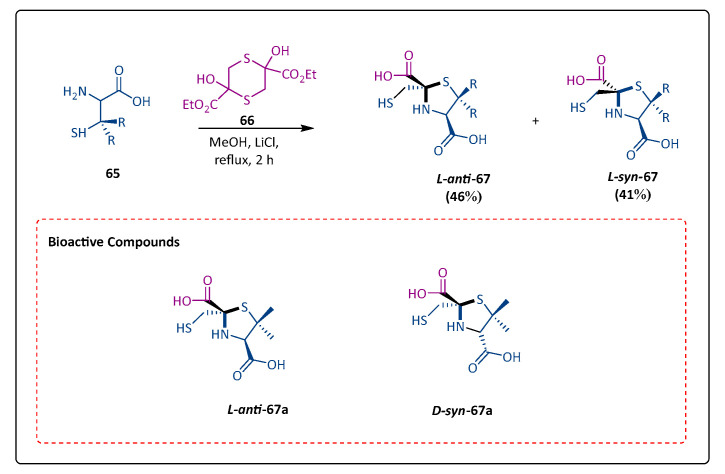
Synthesis of 2-mercaptomethyl-thiazolidines (MMTZs).

Among the most effective commercially available antimicrobial medicines for treating multidrug-resistant bacteria are carbapenems. Because MBLs of category B1 hydrolyze practically all the *β*-lactam antibiotics, the bacterial development of metallo-*β*-lactamases hydrolyzing carbapenem (MBLs) raises concerns about the safety and effectiveness of these antibiotics. MBL inhibitors would extend the half-life of these life-saving medications, thereby meeting an urgent therapeutic need. Rossi et al. synthesized a class of 2-mercapto methyl-thiazolidines (MMTZs) shown to be active in simulating MBL interactions with hydrolysis products or process intermediates. MMTZs exhibit a potent competitive inhibitory effect (K_i_ ¼ 0.44 mM with NDM-1) on B1 MBLs in vitro. The compounds (**65** and **66**) were cyclocondensed to generate MMTZs, which produced mixtures of diastereomers that were simple to purify and assess as pure compounds. The MMTZs that were prepared and isolated were ***L*-anti-67a**, ***D*-anti-67a**, ***L*-anti-67b**, ***D*-anti-67b**, ***L*-syn-67b**, and ***D*-syn-67b**; for MMTZ **67a**, only the anti-diastereomer could be separated. The two most potent NDM-1 inhibitors are ***D*-syn-67b** (0.60 mM) and ***L*-anti-67a** (0.44 mM). The best bisthiazolidine (BTZ) compound, L-BTZ-1, is 12 times more potent as an NDM-1 inhibitor than ***L*-anti-67a** and ***D*-syn-67a**; the K_i_ values of the other MMTZs are either the same as or lower than those of LBTZ (Scheme 13).

**Scheme 14:**
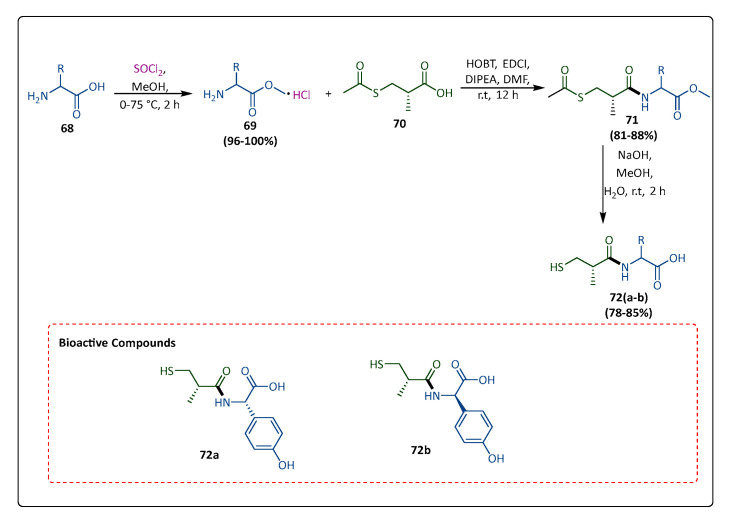
Synthesis of 2-substituted (*S*)-3-mercapto-2-methylpropanamido) acetic acid derivatives.

Public health is severely threatened by the advent and broad growth of metallo-*β*-lactamase (MBL)-mediated resistance to nearly all the *β*-lactam antibiotics. The search is now underway to find novel powerful broad-spectrum MBL inhibitors that are effective against antibiotic resistance, as there are currently no therapeutically relevant MBL inhibitors available. Liu et al. synthesized derivatives of 2-substituted (*S*)-3-mercapto-2-methylpropanamido) acetic acid. Many of these substances demonstrated potent inhibition to the therapeutically significant MBL subtypes with great ligand efficiency, Verona integron-encoded MBL (VIM)-2 and New Delhi MBL (NDM)-1. The compound (**68**) was reacted with SOCl_2_ in the presence of MeOH at 75 °C to produce intermediates (**69**) and (**70**), which were then used for the following step without being purified. Next, in the presence of HOBT, EDCI, and DIPEA, intermediates (**69**) and (**70**) underwent a reaction to yield the corresponding amides (**71**). Ultimately, the desired target compounds (**72(a,b)**) at room temperature were obtained by treating compound (**71**) with aqueous NaOH in methanol. According to molecular-docking investigations of (**72**) binding with NDM-1, (**72**) appears to be positioned to chelate with active-site zinc ions and interact with the catalytically significant residues, such as by creating hydrogen bonds with Lys224 and Asn233. The two most powerful compounds, ‘’**72(a**,**b)’’**, had LE values of 0.42 and 0.1, respectively, and showed inhibitory activities (IC_50_) of 3.57 μM and 5.59 μM, respectively (Scheme 14) [81].

**Scheme 15:**
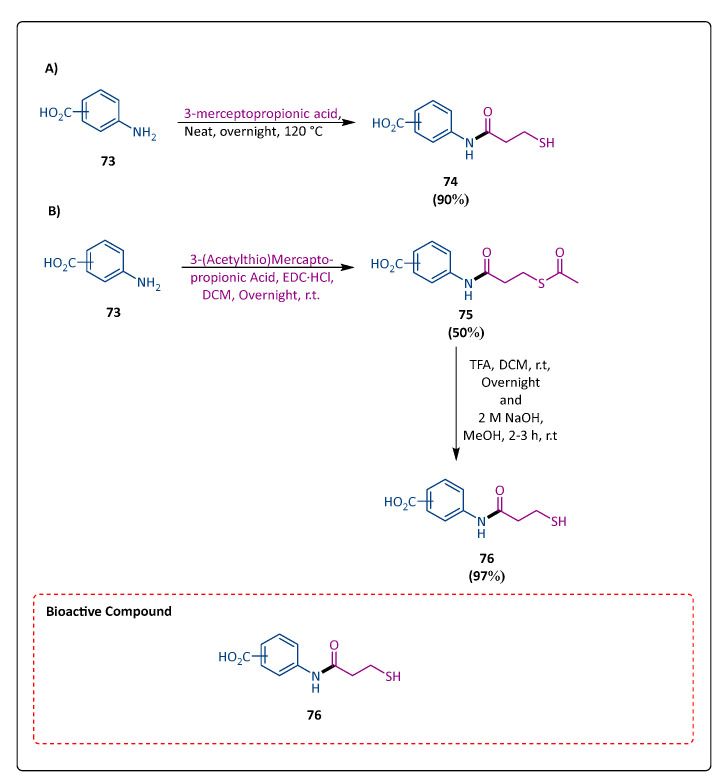
Synthesis of *N*-aryl mercaptoacetamide derivative.

A variety of zinc-containing enzymes known as metallo-*β*-lactamases (MBLs) accelerate the hydrolysis of *β*-lactam substances, including carbapenems, which are the final choices for treating severe infections. To develop strong inhibitors of multiple B1 classes, Kaya et al. synthesized derivatives of *N*-aryl mercaptopropionamide. They also discovered a highly selective hit structure that reinstated the action of imipenem and lowered the minimum inhibitory concentration (MIC) values in resistant *Escherichia coli* isolates by up to 256 times. At 120 °C, aniline (**73**) reacted with 3-mercaptopropionic acid to give compound (**74**) in scheme section A. For the activation of the 3-(acetylthio)-mercaptopropionic acid, they used three different coupling reagents. Activation with EDC·HCl followed by the reaction with the corresponding aniline afforded compound (**75**). At room temperature, BOC deprotection was accomplished in the presence of TFA in dichloromethane (DCM), using sodium hydroxide to hydrolyze thioacetate in MeOH at room temperature and yielding the target compound (**76**) up to a yield of 97% in scheme section B. Hit compound (**76**) had a good log D7.4 value and was found to drastically lower the MIC values of several resistant bacteria that produce MBLs, including clinical isolates, restoring imipenem’s action (Scheme 15) [82].

**Scheme 16:**
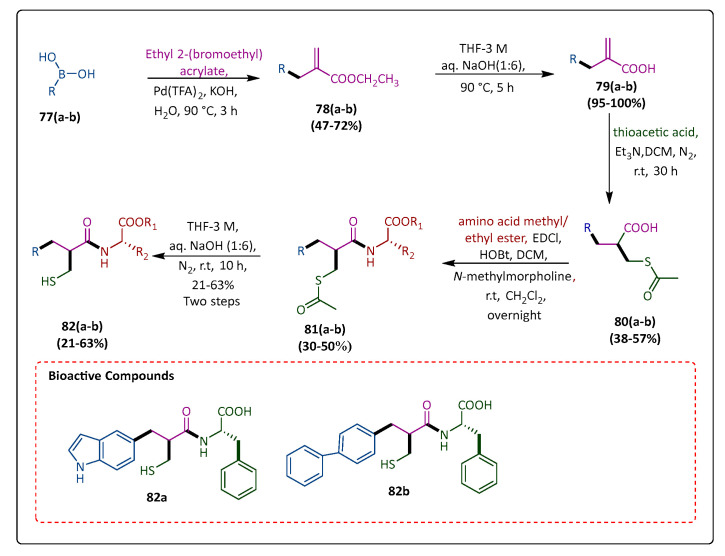
Synthesis of new mercaptopropionamide derivatives.

Global human health is severely threatened by the rise in metallo-*β*-lactamase 1 (NDM-1)-expressing Gram-negative bacteria that are resistant to most *β*-lactam antibiotics. Among the most problematic MBLs is NDM-1. Carbapenems, the antibiotics of “last resort”, are among almost every antibiotic that is a *β*-lactam that NDM-1 can inactivate [83]. Meng et al. synthesized and assessed the NDM-1 inhibitory effects of a range of compounds derived from the main drug, captopril. The NDM-1 inhibitory actions of all the drugs were either submicromolar or micromolar and were significantly more potent than that of captopril. Following a Suzuki coupling reaction [84], the compound (**77(a,b)**) reacted with ethyl 2-(bromomethyl)-acrylate to form intermediates (**78(a,b)**). These were then hydrolyzed with aqueous NaOH to produce compounds (**79(a,b)**). Subsequently, the compounds (**80(a,b)**) underwent a Michael’s addition reaction with thioacetic acid to produce intermediates (**80(a,b)**). These intermediates are then coupled with different methyl or ethyl amino acid esters to produce compounds (**81(a,b)**). Ultimately, the target compounds (**82(a,b)**) were obtained by hydrolyzing these esters (**81(a,b)**). Compounds ‘’**82(a**,**b)**’’ demonstrated remarkable inhibition of NDM-1, as evidenced by their respective IC_50_ values of 0.12 μM and 0.10. Subsequent research revealed that compound **82a** had a low level of acute toxicity and not much cytotoxicity in mice. Crucially, compound **82b** demonstrated strong synergistic antibacterial effects when combined with meropenem (MEM) to treat clinically isolated strains of NDM-1-expressing bacteria (Scheme 16) [85].

**Scheme 17:**
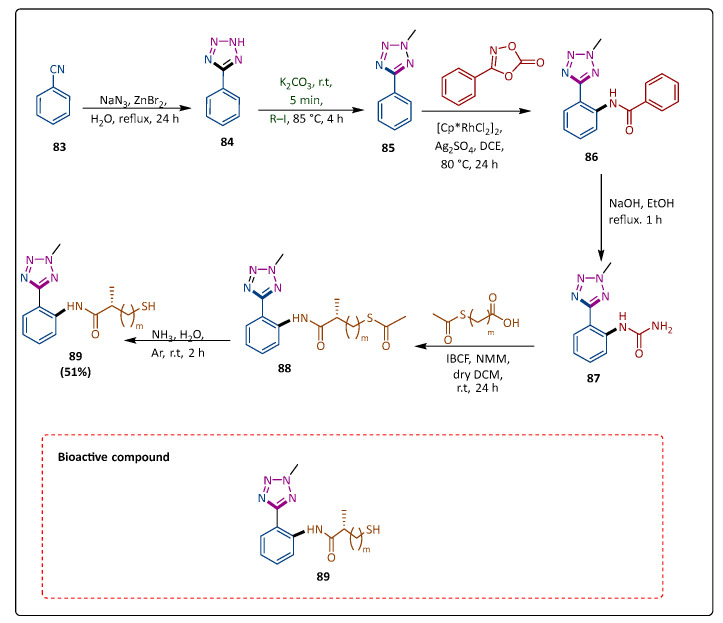
Synthesis of mercaptopropanamide-substituted aryl tetrazoles.

Metallo-*β*-lactamase (MBL)-induced *β*-lactam antibiotic resistance has become a global public health concern. At present, clinical trials are not able to use any MBL inhibitors. Using a hydroxyl group attached to zinc (II), MBLs attack the *β*-lactam carbonyl nucleophilically, breaking the ring in the process. Yan et al. employed C-H activation techniques to synthesize a range of *ortho*- and *meta*-mercaptopropanamide-substituted aryl tetrazole derivatives. They subsequently assessed the compound’s inhibitory potential in opposition to three MBL enzymes, namely, IMP-1, NDM-1, and VIM-2, in the presence of ZnBr_2_ and H_2_O. The compound (**83**) reacted with NaN_3_ to generate compound (**84**) [86], which reacted with iodoalkanes to form compound **85** [87]. Then, compound (**86**) reacted with 3-phenyloxazolidinone to yield the corresponding amides (**86**). Compound **86** further reacted with sodium hydroxide to produce (**87**) [88]. Compound (**87**) produced high yields of thioesters (**88**) by following the reaction with various carboxylic acids. Ultimately, compound (**88**) in the presence of NH_3_SH_2_O in an argon environment produced the required compound (**89**). Compound (**89**) showed strong inhibition of VIM-2 (IC_50_ = 0.035 mM) and moderate inhibitions of IMP-1 and NDM-1 (Scheme 17) [89].

### 5.2. Amino Carboxylic Acid Derivatives

**Scheme 18:**
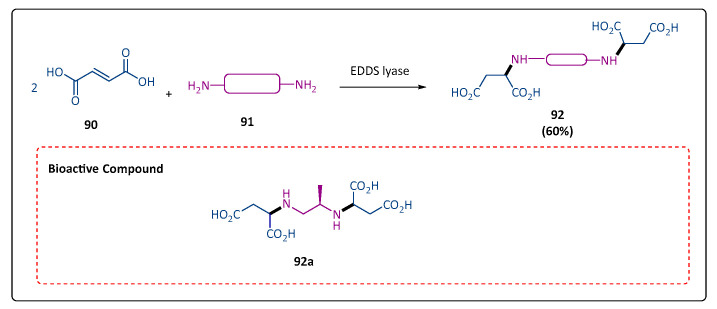
Synthesis of amino carboxylic acid analog.

The NDM-1 metallo-*β*-lactamase in bacteria is strongly suppressed by several AMA and EDDS analogs. The compound’s inhibitory action and its capacity to bind zinc were found to be strongly correlated in isothermal titration calorimetry studies. Tehrani et al. developed several amino carboxylic acid analogs of ethylenediamine-*N*,*N*′-*succinic* acid (EDDS) and *aspergillomarasmine* A (AMA) by chemo-enzymatically synthesizing fumaric acid with a variety of mono- and diamine substrates added, which was conducted under the direction of the enzyme EDDS lyase, in Na_2_HPO_4_ buffer (pH 8.5). The compound (**90**) reacted with the diamine substrate (**91**) and purified EDDS lyase to produce compound (**92**), and (**92a**) showed one of the most effective synergies among the EDDS analogs that were studied (Scheme 18) [90].

### 5.3. Triazole Derivatives

**Scheme 19:**
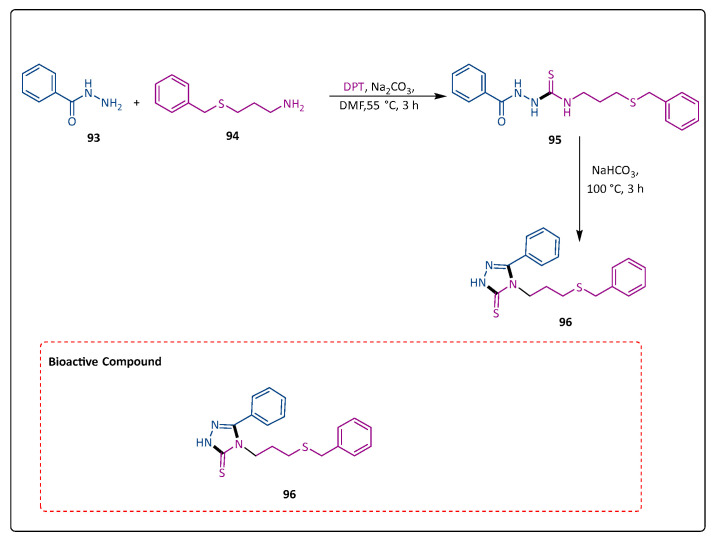
Synthesis of 1,2,4-triazole-3-thione derivatives.

Metallo-*β*-lactamases (MBLs) play a key role in the resistance of Gram-negative bacteria to *β*-lactam antibiotics. Because of their fast proliferation in important human opportunistic infections and carbapenemase activity, MBLs are highly concerning because there is currently no clinically useful inhibitor. Legru et al. developed a new class of compounds, wherein an alkyl-chain-containing thioether and an aryl or carboxylic group at the endpoint is substituted at position 4. The compound (**93**) reacted with compound (**94**) in the presence of dipyridylthionocarbonate (DPT) [2,91] to produce compound (**95**). Ultimately, compound (**95**) underwent cyclization to produce the anticipated 1,2,4-triazole-3-thione molecule (**96**) under basic conditions (NaHCO_3_) in moderate to good yields. The compound (**96**) was found to have a possible inhibitory action on human glyoxalase II, a di-zinc metallohydrolase that is a member of the MBL superfamily. At 52 μM for the IC_50_ value, it could moderately inhibit human glyoxalase II, demonstrating significant selectivity in favor of MBL enzymes (Scheme 19) [92].

**Scheme 20:**
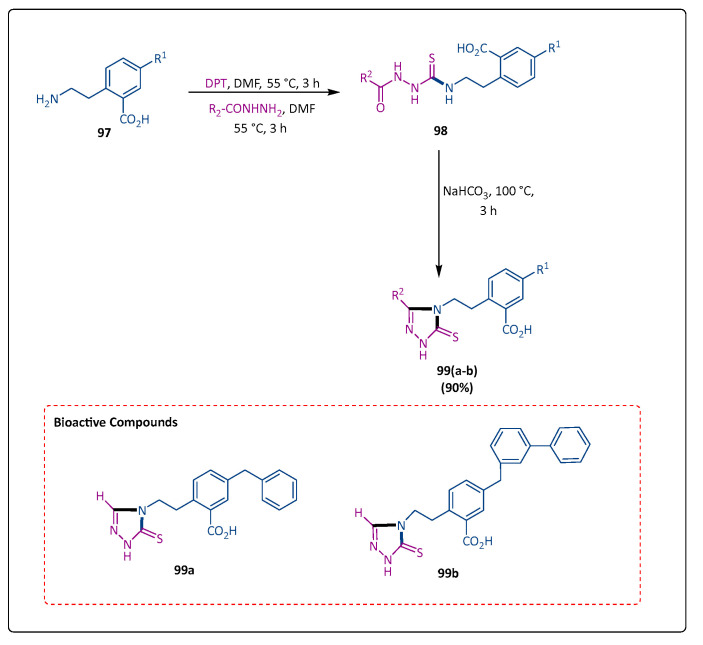
Synthesis of 1,2,4-triazole-3-thione analogs.

Gram-negative bacteria that are opportunistic and resistant to carbapenems are mostly because of the action of metallo-*β*-lactamases (MBLs). L1 [93], IMP-1 [80], VIM2 [94,95,96], and NDM-1 [80,93,96,97] are compounds containing this heterocycle that show MBL inhibition. Verdirosa et al. synthesized novel class of analogs of 1,2,4-triazanalogsone-based inhibitors of Schiff bases, in which an ethyl link containing a benzoic acid group has been substituted for the N-C double bond. Although benzoic compounds only inhibited VIM-type enzymes, switching to an alkyl bond was once more proven to greatly enhance their antibacterial synergistic activity. All the chemicals were synthesized using the following protocol [2,91]: Compound (**97**) was treated with dipyridylthionocarbonate (DPT) and hydrazides to form compound (**98**). The anticipated 1,2,4-triazole-3-thione molecules (**99(a,b)**) were produced under basic conditions (NaHCO_3_). Utilizing K_i_ data, the research validated the greater potencies of **99a** and **99b** in inhibiting VIM-2 in comparison to their hydrazone analogs (K_d_ values of 38 and 61 nM, respectively, versus 68 and 519 nM) (Scheme 20) [91,98].

**Scheme 21:**
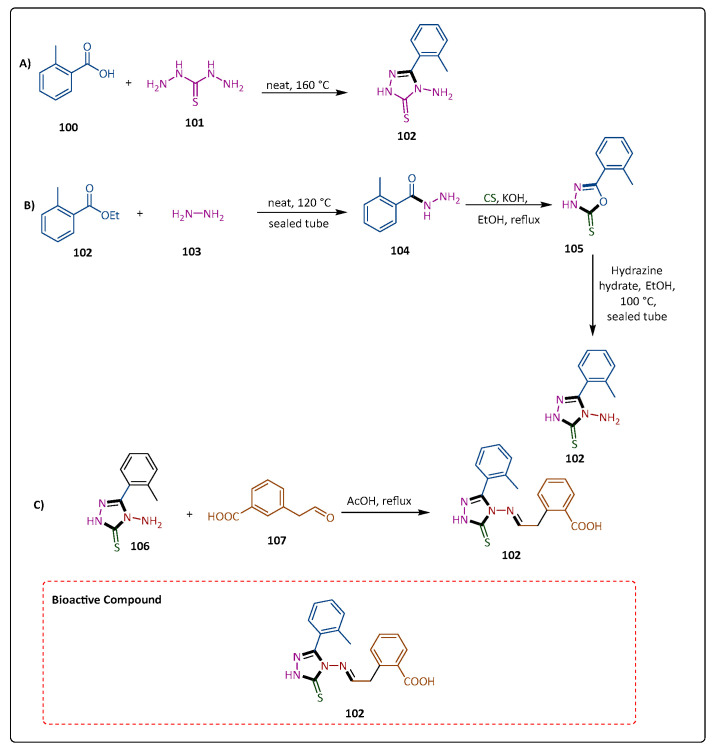
Synthesis of 4-amino-1,2,4-triazole-3-thione derivatives.

The development of metallo-*β*-lactamases (MBLs) produced by Gram-negative bacteria that are resistant to *β*-lactam antibiotics poses a severe medical risk, making the development of clinically effective inhibitors imperative. Only one MBL inhibitor—taniborbactam or VNRX-5133—was able to advance to the clinical development stage [21,22,69,92,99]. Using Olsen’s XIII Schiff base analog, Gavara et al. synthesized new analogs of 1,2,4-triazole-3-thione molecules [97]. The inclusion of an aryl moiety at the heterocycle’s 4-position resulted in more effective inhibitors, with a broad spectrum of the most therapeutically significant MBLs, such as IMP-1, NDM-1, and VIM-2, when compared to IIIA analogs [100]. Compound (**102**) is formed by different procedures in scheme section A, B and C. The substituent at position 5 of the 4-amino-1,2,4-triazole-3-thione compounds was produced according to the prior description [100]. The Schiff base (**102**) is then produced in moderate to good yields when the 4-amine group reacts with benzaldehyde in the presence of acetic acid, introducing the substituent at position 4. For all the examined enzymes, IA: K_i_ = 81 μM (VIM-4) or 108 μM (L1) or inhibition was ≤ 30% at 100 μM (IMP-1, NDM-1, and VIM-2) [27]. Compound (**102**) demonstrated greater inhibitory efficacy compared to its heterocyclic fragment (Scheme 21) [95].

**Scheme 22:**
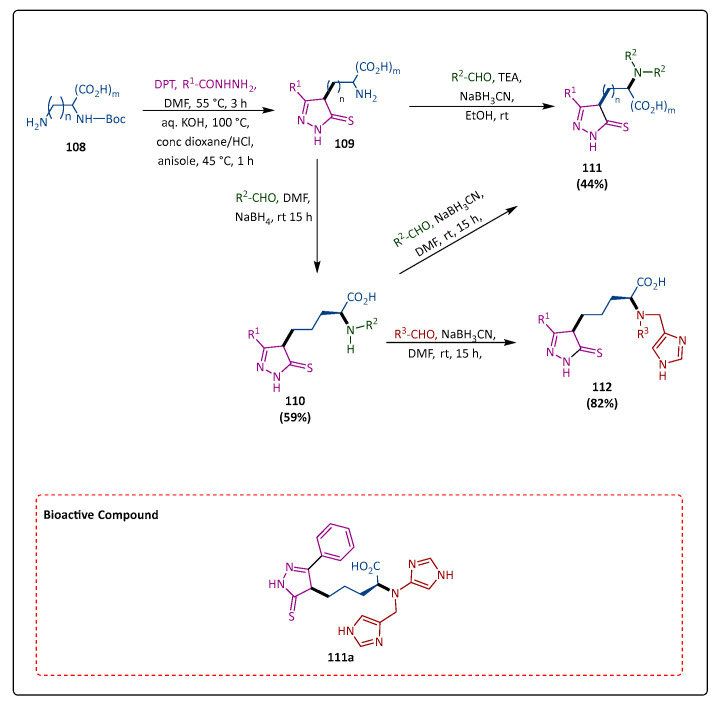
Synthesis of 1,2,4-triazole-3-thione derivatives.

Metallo-*β*-lactamases (MBLs), also known as class B *β*-lactamases, were initially discovered in 1966 and are distinguished by having one or two zinc ions functioning as Lewis acids at the active site [101]. Hospitals use carbapenems as last-resort antibiotics, but Gram-negative bacteria develop resistance to them because of metallo-*β*-lactamases (MBLs). Therefore, MBL inhibitors are critically needed to maintain the efficacy of these vital antibacterial medications. Legru et al. synthesized a class of inhibitors based on 1,2,4-triazole-3-thione with an α-amino acid substituent, whereby the amine was either mono- or disubstituted by (hetero)aryl groups. The commercially available α-Boc-protected diamine compounds (**108**) were treated with DPT (dipyridylthionocarbonate) to form the intermediate isothiocyanates, which were directly reacted with hydrazides to form the thiosemicarbazide derivatives (**109**). Cyclization toward the anticipated 1,2,4-triazole-3-thione molecules was produced by a basic treatment. Finally, the Boc-protecting group was eliminated under acidic conditions in the presence of anisole. Various (hetero)aryl-carbaldehyde compounds are used, and reductive amination is used to replace the deprotected amine. In ethanol, homo-disubstituted analogs (**111**) are often produced by treatment with NaBH_3_CN. Nonetheless, it was discovered that this response was fairly effective. In this case, the homo-disubstituted analogs might be made utilizing NaBH_3_CN on the identical aldehyde in combination with AcOH/DMF to form compound (**112**). An alternative would be to isolate and purify the monosubstituted chemicals before testing. The hetero-disubstituted (**112**) analogs that are produced from the monosubstituted ones use a similar methodology. To obtain just the monosubstituted analogs, the reductive amination step is carried out with NaBH_4_ present. Compound **111a** (JMV706144) had excellent inhibitory efficacy against NDM-1 (K_i_ value of 30 nM) and was a submicromolar inhibitor of both MBL-type VIMs (Scheme 22) [102].

**Scheme 23:**
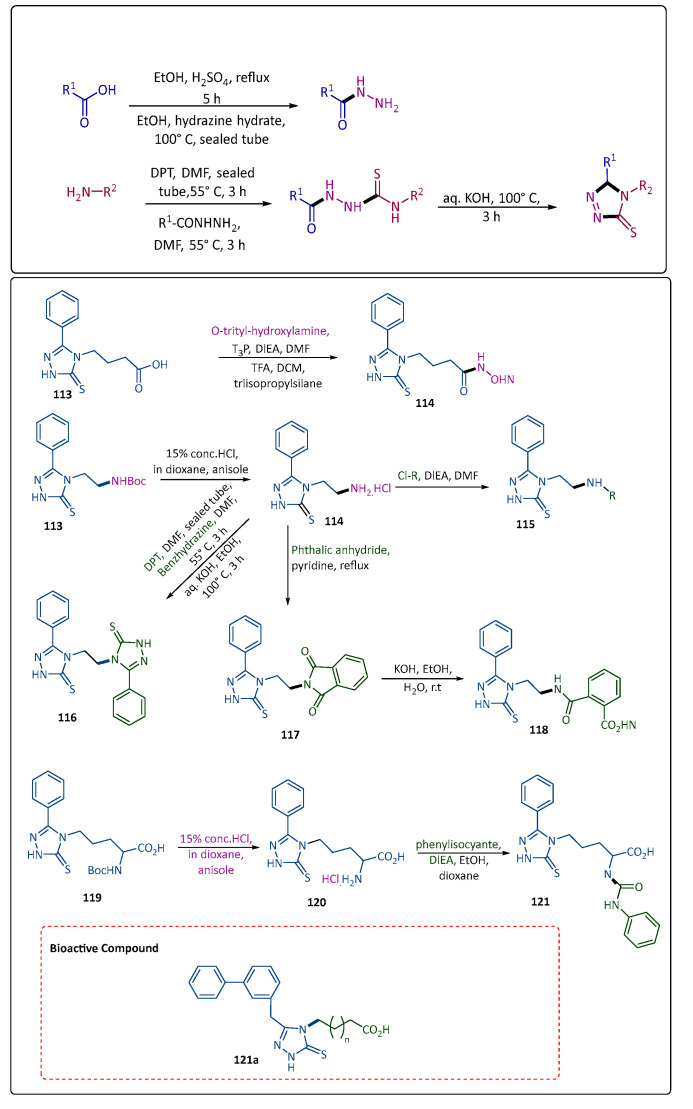
Synthesis of 4-alkyl-1,2,4-triazole-3-thione derivatives.

The primary mechanism by which Gram-negative bacteria resist *β*-lactam antibiotics is by the production of one or more *β*-lactamases (BLs), among which the highly problematic carbapenemases are one. Although these enzymes have recently been commercialized, inhibitors of these enzymes only work against serine carbapenemases (KPC-type, for example), and there is currently no effective inhibitor that can neutralize the metallo-*β*-lactamase (MBL) class. Gavara et al. synthesized a group of compounds with a heterocycle’s 4-position alkyl chain functionalized in a variety of ways. From the corresponding carboxylic acid, the hydrazide precursor of the substituent at position 5 is produced in two stages. Subsequently, DPT and the alkylamines R_2_ and NH_2_ reacted to produce intermediate isothiocyanates, which subsequently reacted directly with hydrazides to form the derivatives of the thiosemicarbazides. They obtain the 1,2,4-triazole-3-thione compounds by their basic treatment. By connecting (**113**) to *O*-trityl-hydroxylamine through trityl elimination in an acidic environment, hydroxamate (**114)** was generated. Compound (**115**) is produced from the Boc-protected precursor, and it reacts with (i) benzoyl and *p*-tosyl chlorides to yield compounds (**116**); (ii) DPT is followed by condensation with benzhydrazide and a hot basic treatment to produce the di-triazole-thione compound (**118**); (iii) phthalic anhydride is refluxed with pyridine to produce the phthalimide (**117**), which opened under basic conditions to yield (**116**). Compound (**119**) is produced from (**120**) by Boc removal, and it condensed to phenyl isocyanate to produce urea (**121**) in a good yield. Ultimately, the binding mechanism of the alkanoic analogs in the VIM-2 active region was investigated using docking assays. With K_i_ values of 5.50, 0.35, and 0.27 μM, respectively, compound **121a** showed the strongest activities against VIM-1, VIM-2, and VIM-4 in this series, demonstrating the interest of an *m*-biphenyl group at position 5 (Scheme 23) [2,95,100,103].

**Scheme 24:**
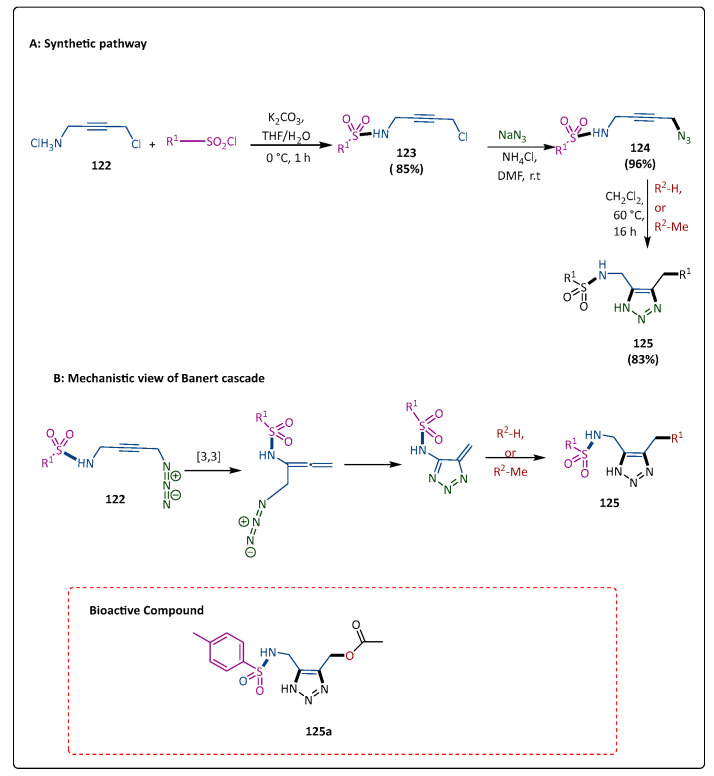
Synthesis of NH-1,2,3-triazole.

Metallo-*β*-lactamases (MBLs) hydrolyze all types of *β*-lactams, except monobactams. MBLs are a growing cause of antibiotic resistance in bacteria. Moreover, currently available serine-*β*-lactamase inhibitors do not reduce MBL activity. Muhammad et al. prepared numerous NH-1,2,3-triazoles using a three-stage procedure, with the Banert cascade reaction acting as a critical step. Biochemical experiments were used to assess the inhibitors’ characteristics against the MBLs GIM-1, NDM-1, and VIM-2. VIM-2 demonstrated IC_50_ values as low as the nanomolar range. The compound (**122**) produced chlorosulfonamides (**123**) by reacting with sulfonyl chlorides in a base (K_2_CO_3_), which then transformed to the corresponding azidosulfonamides (**124(a–d)**). In the presence of a variety of nucleophiles, the crude azides (**125**), carrying various sulfonamide groups, underwent the Banert cascade [104,105,106]. For a mechanistic proposal of the Banert cascade, see [106,107,108]. According to the docking, the findings unequivocally demonstrate that the inhibitors do not substitute the hydroxide ion and that the triazole moiety only interacts with one of the two zinc ions (Zn^2^), while the sulfonamide group is not involved in the zinc-binding process. With reasonable IC_50_ values of 48 μM (GIM-1), 23 μM (VIM-2), and 231 μM (NDM-1), **125a** was the most promising inhibitor (Scheme 24) [79].

### 5.4. ANT2681

**Scheme 25:**
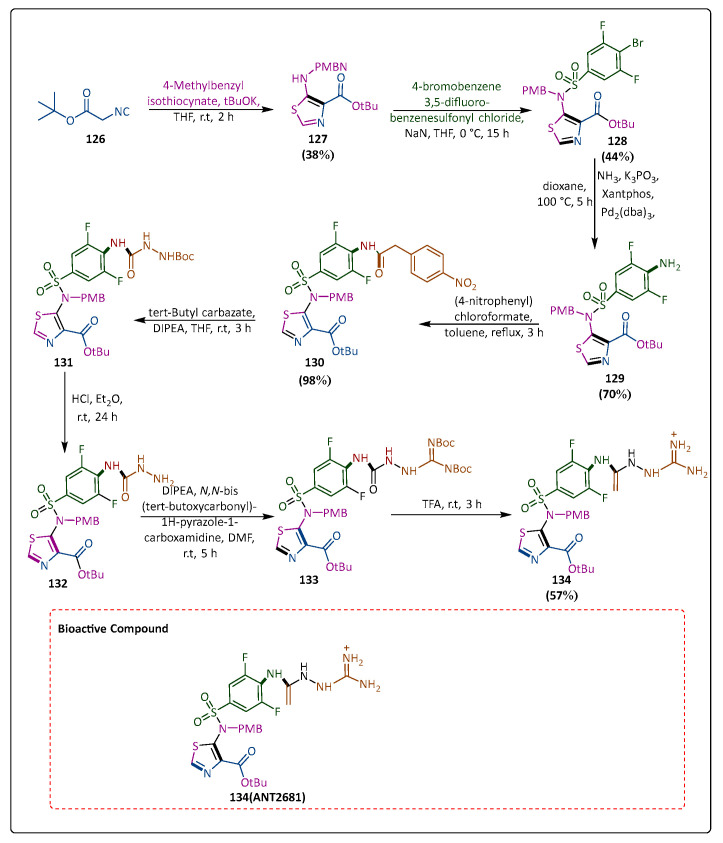
Synthesis of aminoguanidine analog.

The development of acquired metallo-*β*-lactamases and enzymes of serine lactamase is the primary cause of resistance, which threatens the therapeutic efficacy of the crucial *β*-lactam class of antibiotics. Over the past few decades, numerous combinations of *β*-lactam/*β*-lactamase inhibitors have been effectively introduced to clinics to address this resistance issue. Davies et al. reported an understanding of a lead optimization campaign that led to the synthesis of the aminoguanidine analog ANT431 by palladium-assisted aminolysis and the identification of the candidate of preclinical ANT2681, a powerful inhibitor of NDM with great potential for clinical use. The compound (**134**) is known as ANT2681. The aminolysis of the bromosulfonamide with palladium catalysis, aniline chloroformate activation, and interaction with Boc-protected hydrazine via the isocyanate were the steps involved in the production of the crucial aminoguanidine analog (**133**). Then, (**134**) was obtained by selectively removing the Boc group and *t*-butyl ester, guanidinylation, global deprotection, and HPLC purification. Compound (**134**) can inhibit various MBL enzymes. Compound (**134**) also inhibited additional MBLs, and when combined with meropenem, it was effective against a variety of *Enterobacteriaceae* that produced MBLs (Scheme 25) [109].

### 5.5. Thiol Derivatives

**Scheme 26:**
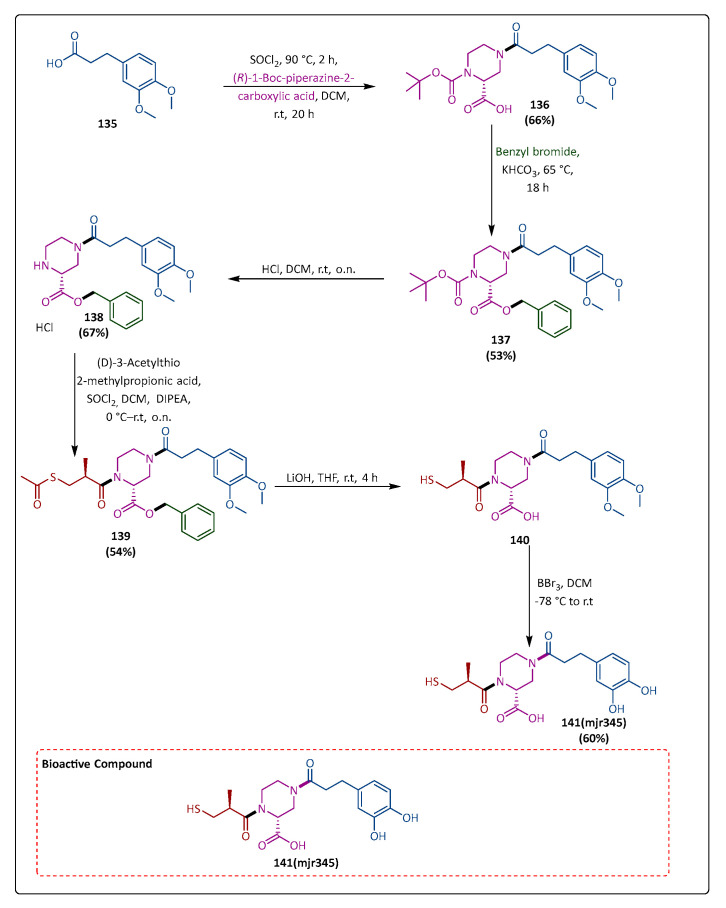
Synthesis of thiol-based inhibitors by integrating catechol moieties.

Metallo *β*-lactamases (MBLs) are among the most challenging antibiotic-resistant resistance mechanisms seen in Gram-negative bacteria because of their broad substrate range and lack of known inhibitors. Inhibitors of thiol-based MBL, such as thiorphan, captopril, tiopronin, and unithiol, have been the subjects of multiple reports [110]. MBLs are strongly inhibited by a variety of thiol-containing inhibitors. To generate thiol-based MBL inhibitors, Rotter et al. reported catechol compounds. They additionally developed two dimethoxy analogs of catechol-containing M*β*L inhibitors and assessed their inhibitory activities against NDM-1, IMP-7, and VIM-1 in vitro. The compound (**135**) reacted with (*R*)-1-Boc-piperazine-2-carboxylic acid to give compound (**136**), which further reacted with benzyl bromide to form compound (**137**). Then, compound (**137**) undergoes deprotection to yield compound (**138**). Compound (**138**) reacted with (*D*)-3-acetylthio-2-methylpropionic acid to generate compound (**139**). The mercaptomethylpropionic acid (**139**), under Schotten–Baumann conditions, combined with LiOH to form compound (**140**). The required compound (**141**) is obtained through deprotection processes using BBr_3_. Compound (**141**) reduced all the examined M*β*Ls to submicromolar levels, broadening the spectrum of M*β*L inhibitors based on D-pipecolic acid. Because of this, it works better than captopril or tiopronin, which has an activity assay with similar results, displaying inhibitory levels in the low-micromolar range (Scheme 26) [110,111].

**Scheme 27:**
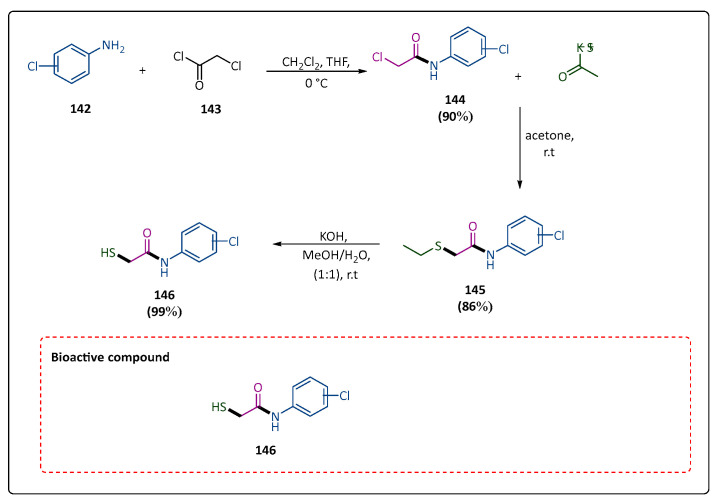
Synthesis of the thiols.

The lack of novel, effective, and imaginative therapeutic options and the emergence of drug-resistant bacteria make it imperative to treat conditions caused by Gram-negative pathogens, in particular. Innovative methods concentrate on virulence rather than the growth of bacteria. Restoring the effectiveness of antibiotics currently in clinical use is another tactic. Inhibiting resistance factors, like metallo-*β*-lactamases (MBLs), can help to achieve this. Yahiaoui et al. described the synthesis and in vitro evaluation of *N*-aryl-mercaptoacetamides as inhibitors of the virulence factor (LasB) of *Pseudomonas aeruginosa* and clinically important MBLs. VIM-1 (Verona integron-encoded metallo-*β*-lactamase), NDM-1 (New Delhi metallo-*β*-lactamase), and IMP-7 (imipenemase) enzyme tests revealed from low micromolar to submicromolar activities for all the investigated *N*-aryl-mercaptoacetamides. The target compound (**146**) was obtained by a three-step synthetic process that began with the matching of aniline derivatives. The first step involves forming an amide by adding chloroacetyl chloride dropwise to a cold solution of the appropriate aniline and DIPEA in THF or CH_2_Cl_2_. Second, the thioacetate (**145**) was produced by an S_N_2 reaction between potassium thioacetate in acetone and the alkyl chlorides (**144**). Aqueous KOH hydrolyzed compounds (**145**) in MeOH to produce compound (**146**). With submicromolar IC_50_ values for both NDM-1 and IMP-7 and low-micromolar activity against VIM-1, compound **150** showed the highest inhibitory effects (Scheme 27) [112].

**Scheme 28:**
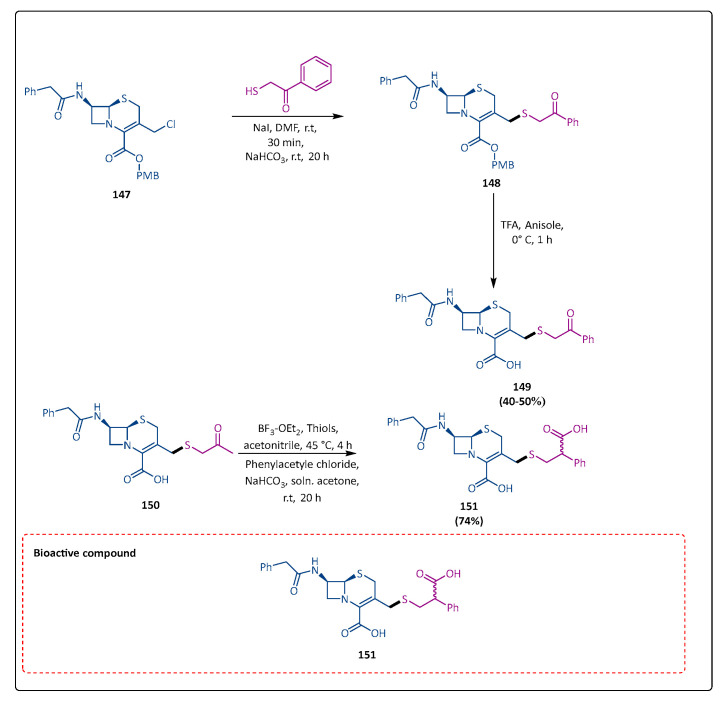
Synthesis of cephalosporin–thiol conjugates.

There are currently no approved medications that target metallo-*β*-lactamases (MBLs) on the market, despite the growing concern about resistance to *β*-lactam antibiotics caused by these enzymes. Several other substances have been identified as MBL inhibitors; most of these substances work by either sequestering zinc or by joining forces with metalloenzymes to create ternary complexes [113,114]. Tehrani et al. developed and produced an array of innovative cephalosporin prodrugs to administer thiol-based metallo-*β*-lactamase (MBL) inhibitors in a regulated spatiotemporal manner. There are two methods for synthesizing the cephalosporin–thiol conjugates. Mercaptoacetophenone is alkylated with the chloromethyl cephalosporin “GCLE” (**147**) to generate compound (**148**), which is then deprotected with trifluoroacetic acid to yield compound (**149**). As an alternative, compound (**150**) was generated by acylating the 7-amino group after substituting the appropriate thiols for compound (**151**). This process was facilitated by BF_3_ [115]. Studies on the structure–activity relationship have shown that **151**’s effectiveness depends on its carboxyl and phenyl moieties. Additionally, research on modeling suggests that the potency and selectivity of (**151**) may be attributed to the fruitful interactions that the mandelic acid molecule of (**151**) has with Trp28 within the IMP active site (Scheme 28) [116].

**Scheme 29:**
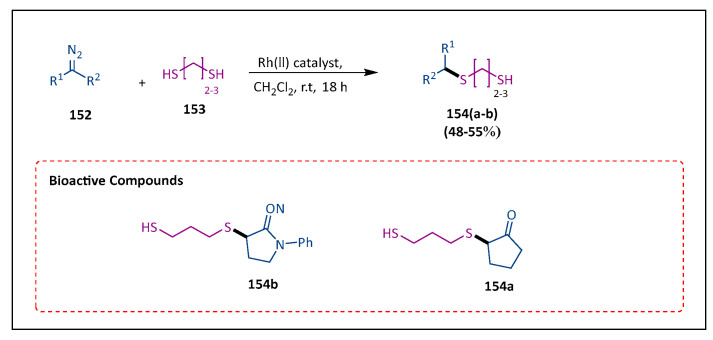
Synthesis of alkylthio-substituted thiols.

The most common form of *β*-lactamases efficiently protects bacteria against *β*-lactam antibiotics, such as cephalosporins and last-resort penems [117]. The *β*-lactam hydrolysis process mediated by *β*-lactamases requires the presence of nucleophilic metallic ions (in metallo-*β*-lactamases, MBLs) or serine residues (in serine-*β*-lactamases, SBLs) at the active site of the enzyme. The inhibition of the MBL may be mediated using inhibitors containing a thiol compound. Thiols hold Zn^2+^ ions in place at the MBL active site [20]. Krasavin et al. found an effective S-H insertion reaction of α-diazo-*γ*-butyrolactams with a range of aromatic and aliphatic thiols using Rh(II) catalysis [118]. Rh(II) catalyzes an effective S-H insertion reaction between α-diazo-*γ*-butyrolactams and several aromatic and aliphatic thiols. The reaction proceeded after the matching Rh(II) carbenes were coupled to propane-1,3-dithiol, ethane-1,2-dithiol, or both. This resulted in the desymmetrization of the latter and the synthesis, in modest to good yields, of alkylthio-substituted thiols (**154(a,b)**). Molecular docking studies were carried out using the most straightforward analog, (3a), and the strongest derivative (**154b**). Thus, the X-ray structures of **154a** and **154b** were docked with structures of both potential enantiomers, NDM-1 in combination with a thiol-containing compound (PDB code 4EXS), the L-captopril inhibitor. The **154a** docking mode that was gained demonstrated that the thiol group, which was thought to be free, is situated between the Zn^2+^ ions in the negatively charged center of the catalysis. As a result, the polarized water is moved, accounting for the hydrolysis of the *β*-lactams. The most efficient molecule was compound **154b**, which exhibited a balanced inhibitory activity against both enzymes. For VIM-1, its IC_50_ values were 0.02 μM, while for NDM-1, they were 0.3 μM. When docked, the strongest derivative (**154b**) and the simplest counterpart (**154a**) show the same predominant interactions (Scheme 29) [119].

### 5.6. Indole Carboxylate EBL-3183

**Scheme 30:**
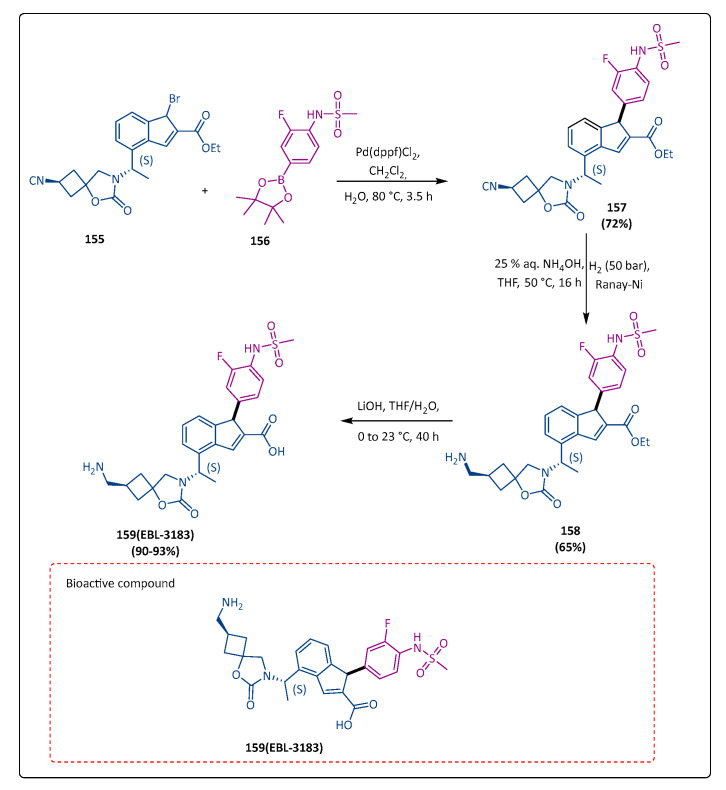
Synthesis of indole carboxylate EBL-3183.

Very few MBL inhibitors, with a breadth of potency against the three main classes of MBLs—NDM, IMP, and VIM variants—that are probably needed for widespread clinical usage, have been identified, and even fewer have any true potential for clinical development [120]. A new synthetic method that complies with the scale and purity requirements for preclinical research has been developed to prepare new MBL inhibitors. Baran et al. developed a new synthetic pathway and executed it at the kilogram level to generate EBL-3183. EBL-3183 is a potential metallo-*β*-lactamase inhibitor precandidate. Compound (**155**) and compound (**156**) reacted via stereoselective reduction catalyzed by ruthenium to form compound (**157**). For the preclinical research, there was plenty of the ultimate target (EBL-3183) in terms of quantity and quality. Then, compound (**157**) was reduced using Raney Ni to produce compound (**158**), which subsequently reacted with LiOH in the presence of THF/H_2_O to form compound (**159**), which is the desired product (EBL-3183). The precandidate (**159**, EBL-3183) for a new MBL inhibitor complies with preclinical research’s scale and purity requirements (Scheme 30) [121].

### 5.7. Aryl Sulfonyl Hydrazone Derivatives

**Scheme 31:**
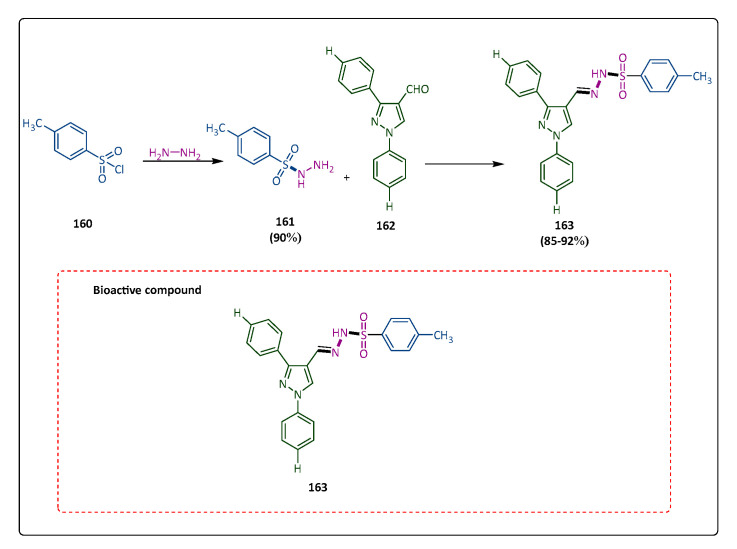
Synthesis of aryl sulfonyl hydrazones that are conjugated.

Class B includes metallo-*β*-lactamases (MBLs), which have zinc ions with divalent structures. Most penicillins*,* cephalosporins, and carbapenems are rendered as inactive by these enzymes through the utilization of zinc-bound hydroxyl groups [122]. Using *Klebsiella pneumoniae* NDM-1 as a model, Shaaban et al. synthesized metallo-*β*-lactamases (MBLs) from aryl sulfonyl hydrazones coupled with 1,3-diaryl pyrazoles. For the majority of the proposed compounds, the in vitro MBL inhibition demonstrated a significant inhibitory constant against AIM-1, IMP-1, NDM-1, and NDM-1 MBLs at a low micromolar range (1.5–16.4 μM). Compound (**160**) reacted with hydrazine to produce compound (**161**)**.** By the condensation of aldehyde (**162**) with (**161**) in heated ethanol, compound (**163**) is produced [123]. The in vitro MBL inhibition observed was justified through docking experiments conducted on NDM-1 and IMP-1. The promising pharmacokinetic and drug-like characteristics of the active compound (**163**) were shown using in silico predictions. Overall, this study offers a useful framework for expanding the chemical space where metallo-*β*-lactamases can be inhibited. This makes use of eco-friendly and sustainable practices. When it came to clinical isolate K_i_, (**163**) had the best antibacterial activity (Scheme 31) [124].

### 5.8. Triazolethioacetamide Derivatives

**Scheme 32:**
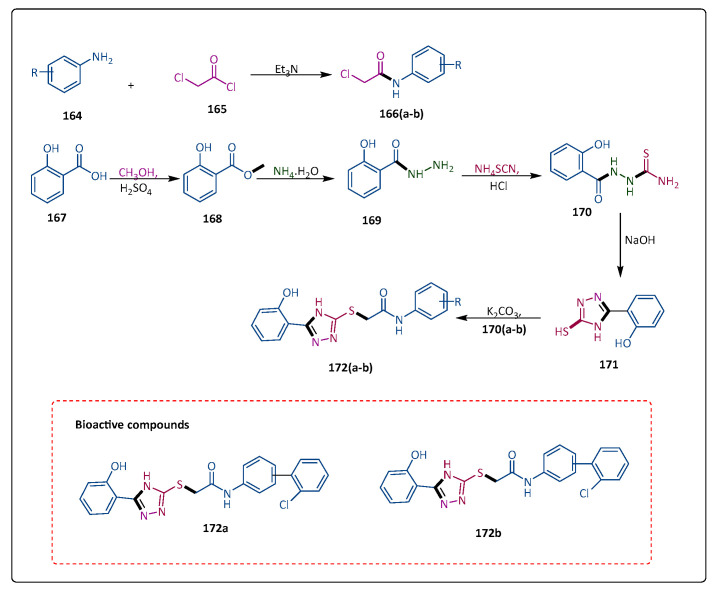
Synthesis of halogen-substituted triazolethioacetamides.

The term metallo-*β*-lactamases (M*β*Ls) refers to the enzymes that target antibiotic resistance to *β*-lactams. One or two Zn ions are active at metallo-*β*-lactamase (M*β*L) active sites. There are currently no clinically available, efficient inhibitors against M*β*Ls, despite the discovery of numerous promising inhibitor molecules in recent publications [113]. Zhang et al. produced triazole thioacetamides replaced with halogens, which are highly effective inhibitors of M*β*Ls. IC_50_ values range from 0.032 to 15.64 μM, and every drug showed inhibitory action against ImiS. The halogen-substituted triazolethioacetamides are synthesized as follows: Briefly, compound (**164**) reacted with compound (**165**) in the presence of triethylamine to form compound (**166**). Then, compound (**167**) reacted with methanol to form compound (**168**), which reacted with ammonium hydroxide to give compound (**169**). Compound (**169**) further reacted with ammonium thiocyanate to form compound (**170**), which reacted with sodium hydroxide to generate compound (**171**). Then, the crosslink between compound (**171**) and compounds (**166(a,b)**) gives the corresponding target compounds (**172(a,b)**) in moderate to good yields. Each compound was identified using ^1^H and ^13^C NMR, and MS verification was obtained. Docking studies showed that **172a** and **172b**, which had the strongest inhibitory effects on ImiS, suit the binding site of CphA as a substitute of ImiS between the triazole group bridging ASP120 and the hydroxyl group bridging ASN233 (Scheme 32) [125].

### 5.9. H_2_dedpa Derivatives

**Scheme 33:**
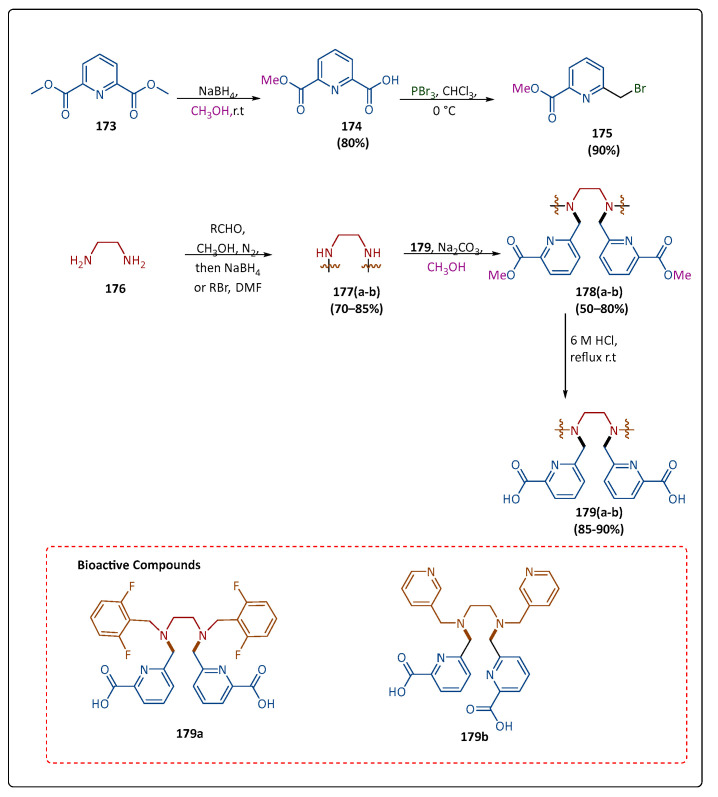
Synthesis of H_2_dedpa derivatives.

NDM-1, an enzyme dependent on zinc (II), can hydrolyze nearly all the antibiotics of beta-lactams, including carbapenems. This is a risk to global health and may lead to bacterial antibiotic resistance. Cui et al. carefully produced a variety of H_2_dedpa compounds, predicated on the discovery that H_2_dedpa (1,2-[[6-carboxy-pyridin-2-yl]-methylamino]ethane) demonstrates a potent inhibition of NDM-1. IC_50_ values for these drugs ranged from 0.06 to 0.94 μM, indicating remarkable potency against NDM-1. The compound (**175**) was first prepared by the partial reduction of compound (**173**), followed by the bromination of the alcohol (**174**). Compounds (**177(a,b)**) were prepared by reductive amination with different aldehydes. The reaction between (**175**) and (**177(a,b)**) lead to the formation of the esters (**178(a,b)**). Finally, ester hydrolysis produced the target compounds (**179(a,b)**). According to the docking studies, compounds **179a** and **179b** inhibit NDM-1 by binding with the Zn^2+^ ions, with the carbonyl group of one **179a** carboxylic acid molecule binding with two zinc ions of NDM-1 via coordinate bonds. Compounds **179a** and **178b** can restore meropenem’s effectiveness against strains of Proteus *mirabilis*, *Klebsiella pneumoniae*, and *Escherichia coli* that have either IMP or NDM. Mechanistic analyses showed that chemicals **179a** and **179b** block NDM via the chelation of the enzymes with Zn^2+^ ions (Scheme 33) [126].

### 5.10. Rhodanine Derivatives

**Scheme 34:**
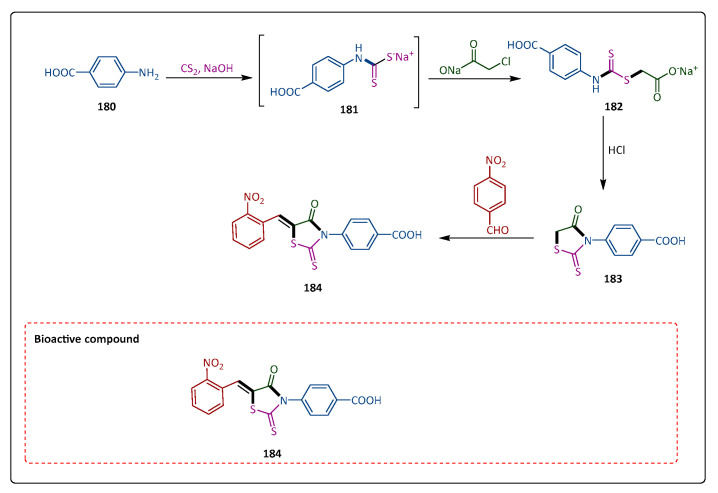
Synthetic route of rhodanine derivatives and thioenolates.

Over the past seven decades, the development of antibiotics of *β*-lactams has made medications to treat a variety of bacterial illnesses available. In response to the rise in M*β*L-mediated drug resistance, numerous M*β*L inhibitors, including *β*-lactam analogs, hydroxamic acids, azolyl thioacetamides, and cyclic boronates, have been described. Xiang et al. described a group of rhodanines as powerful scaffolds for the synthesis of a wide range of metallo-*β*-lactamase inhibitors, with their *Z*-conformations verified by X-ray crystal structures of small molecules and their anti-metallo-*β*-lactamase (M*β*L) activities quantified. The compound (**180**) and carbon disulfide reacted for 16 h at room temperature in an aqueous NaOH solution to generate compound (**181**). Compound (**181**) was reacted with sodium chloroacetate to form compound **182**, and then compound (**182**) was acidified with HCl and refluxed for 16 h to yield *N-*substituted rhodanines (**183**) [127]. The required rhodanine (**184**) was obtained in a quantitative yield via the Knoevenagel condensation of the aryl aldehyde and in acetic acid [128,129]. According to docking studies, one or two Zn(II) ions are coordinated with compound (**184**). In contrast, the inhibitor’s *N*-phenyl group strengthens its hydrophobic contact with M*β*Ls. These investigations show that the diaryl-substituted rhodanine makes useful building blocks for the production of upcoming wide-range inhibitors of M*β*Ls (Scheme 34) [130].

**Scheme 35:**
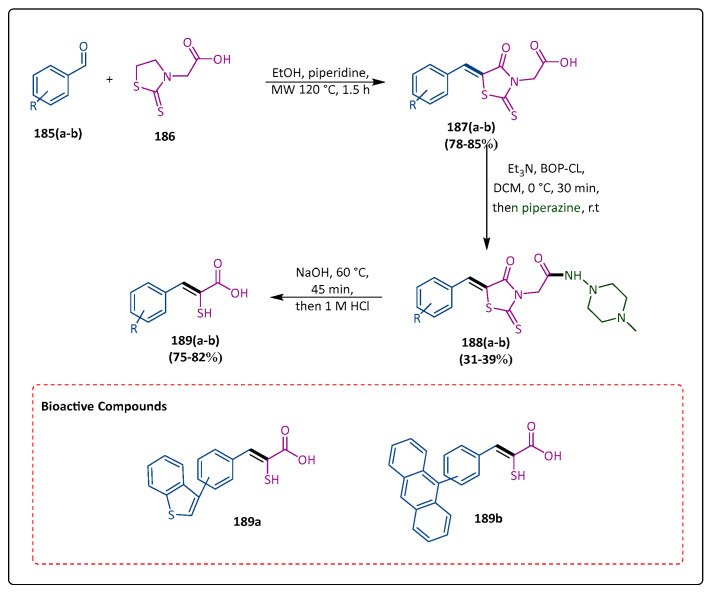
Synthesis of rhodanine-derived ene–rhodanine derivatives.

Bacterial resistance to practically all kinds of antibiotics of *β*-lactams is made possible by metallo-*β*-lactamases (MBLs). Zhang et al. present research on M*β*L inhibitors that contain enethiols and were synthesized through the hydrolysis of rhodanine. The M*β*Ls from various subclasses are inhibited by the enethiols. Studies have revealed the connection between derived enethiol inhibitors of metallo-*β*-lactamases and rhodanines in terms of structure–activity relationships. Compounds (**189(a,b)**) were prepared in two steps, by the Knoevenagel-type condensation of compound **185** and compounds (**186(a,b)**) to provide, predominantly, derivatives of (**187(a,b)**), [128,131] which were coupled with compound **188** to give the desired analogs (**189(a,b)**) [132]. Compounds having elevated steric bulk, such as thianaphthene (**189a** or **189b**), demonstrated strong or rather-strong inhibitions against the majority of the M*β*Ls, with one distinct exception in each instance (NDM-1 with **189a** and BcII with **189b**) (Scheme 35) [133].

### 5.11. Isatin-β-Thiosemicarbazone Derivatives

**Scheme 36:**
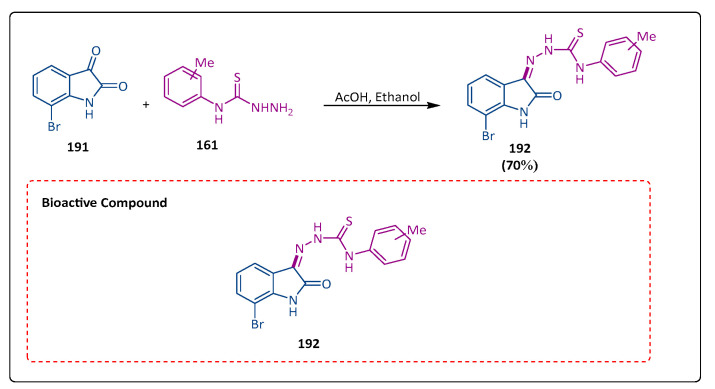
Synthesis of isatin-*β*-thiosemicarbazone derivatives (IBTs).

Bacterial resistance to *β*-lactam antibiotics is conferred by New Delhi metallo-*β*-lactmase-1 (NDM-1) cleaving the *β*-lactam ring and hydrolyzing the antibiotics. Song et al. synthesized a group of isatin-*β*-thiosemicarbazones (IBTs) and assessed them physiologically as new NDM-1 inhibitors. For all the IBT compounds, IC_50_ values were less than 10 μmol/L, with 2.72 μmol/L being the highest. In the presence of ethanol and AcOH, compound **(191**) and compound (**161**) reacted to generate compound (**192**). The docking study revealed that compound (**192**) interacted with NDM-1 residues, forming hydrophobic interactions and hydrogen-bonding contacts. Zinc ions form coordination bonds with sulfur atoms, indicating their essential roles in inhibition. Compound (**192**) is the strongest, exhibiting an IC_50_ value of 2.72 μmol/L, which provides excellent NDM-1 inhibition results (Scheme 36) [134].

### 5.12. Zndm19

**Scheme 37:**
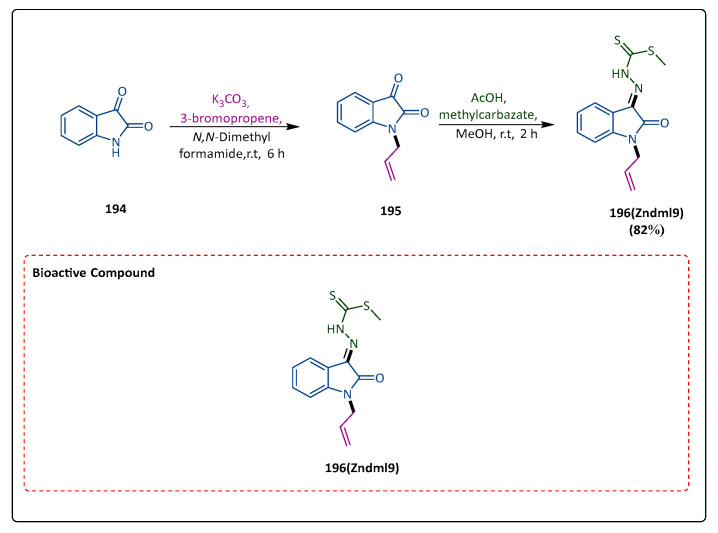
Synthesis of Zndm19.

Because it can hydrolyze almost all the *β*-lactam antibiotics, New Delhi metallo-*β*-lactamase-1 (NDM-1) is a hazard to public health because it leaves NDM-1-positive bacteria with few alternatives for treatment. Unfortunately, no efficient NDM-1 inhibitors are being used in clinical practice at this time. This forces us to look for new chemicals to fight bacterial infections that are resistant to drugs (MDR). Using the checkerboard method, time-killing assay, and combined disk test, Lv et al. evaluated the synergistic bactericidal effectiveness of Zndm19 in conjunction with meropenem (MEM). Zndm19 was shown to be a novel NDM-1 inhibitor by the use of virtual screening and an NDM-1 enzyme activity inhibition assay. Imidization and nucleophilic addition were the two primary synthetic methods utilized for producing the target molecule. Acidification with acetic acid helped to speed up the reaction during the synthesis of Zndm19 (**196**). With no need for further purification, the total yield was 82.8%. The bactericidal action of the MEM against NDM-1-positive *Escherichia coli* was restored in vitro using molecular docking to show that 16 μg/mL of Zndm19 (**196**) reduced NDM-1’s activity without changing NDM-1’s expression (Scheme 37) [135].

### 5.13. Sulfahydantoin Derivatives

**Scheme 38:**
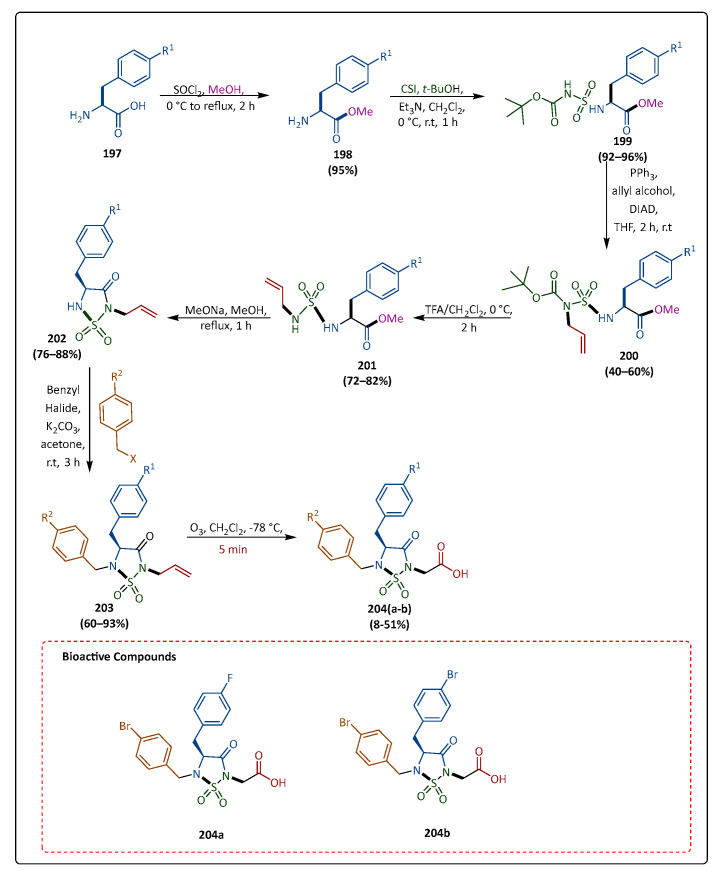
Synthesis of sulfa hydantoin derivatives.

Sulfahydantoin-based compounds could be able to address the growing problem of antibiotic resistance. These compounds have the potential to function as *β*-lactamase enzyme inhibitors, which are essential for specific resistance mechanisms. Cote et al. synthesized six new sulfahydantoin derivatives by cyclizing α-amino-acid-derived sulfamides produced by the critical reaction of chlorosulfonyl isocyanate to sulfahydantoins. The fast synthesis allows for the production of the necessary molecules in eight steps. Compound (**198**) was formed when compound (**197**) reacted with thionyl chloride in the presence of methanol. After that, compound (**199**) was generated by only mixing compound (**198**) with *t*-butyl alcohol with chlorosulfonyl isocyanate (CSI). Subsequently, compound (**199**) reacted with allyl alcohol and diisopropyl azodicarboxylate (DIAD) to form compound (**200**), which further reacted with trifluoroacetic acid to form compound (**201**). Compound (**202**) was formed when compound (**201**) reacted with sodium methoxide in anhydrous methanol. Compound (**202**) reacted with benzyl halide to form compound (**203**). Then, potassium peroxymonosulfate (Oxone) was added right away to produce the final desired products (**204(a,b)**). The *β*-lactamases are significantly inhibited by two compounds, **204a** and **204b**; their inferred K_i_ values range from 32 to 55 μM, and their IC_50_ values vary from 130 to 510 μM (Scheme 38) [136].

### 5.14. α-Aminophosphonate Derivatives

**Scheme 39:**
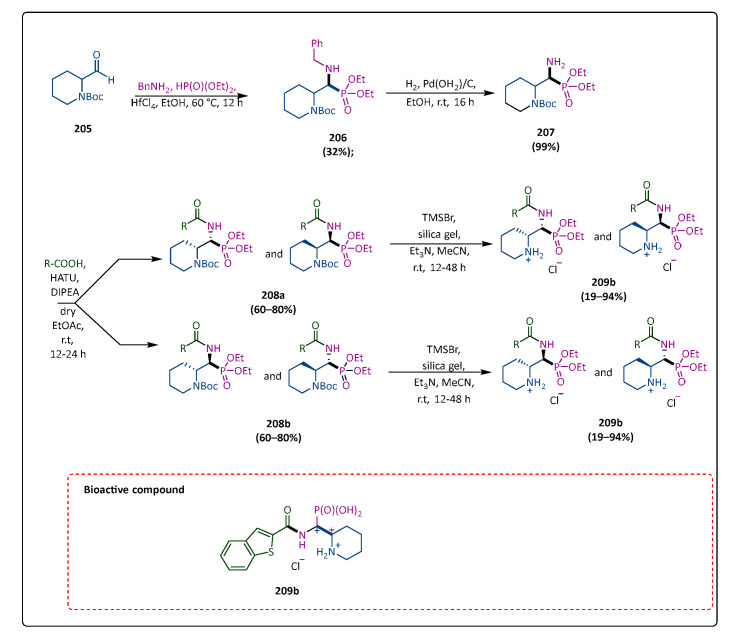
Synthesis of *α*-aminophosphonate derivatives.

The threat to human health posed by the rise in antibiotic resistance is growing. Because bacterial metallo-*β*-lactamases break down the most commonly prescribed family of antibiotics, *β*-lactams, we are especially concerned about resistance caused by these enzymes. The usage of currently available antibiotics of *β*-lactams, such as carbapenems, ns, penicillins, and cephalosporins, which usefulness is progressively decreasing, may be permitted if metallo-*β*-lactamases are inhibited. Palica et al. produced a wide range of novel, non-cytotoxic inhibitor candidates derived from *α*-aminophosphonate and revealed the results of the computational analysis and solution NMR spectroscopy analysis of the VIM-2 and NDM-1 binding locations and binding modalities. Through a process that uses the Kabachnik–Fields reaction catalyzed by hafnium chloride [88], which concurrently introduces the amine and phosphonic acid functional groups, (**209**) is synthesized. Next, Pearlman’s catalyst was used to gently hydrogenate the benzyl-protecting group of compound (**206**) [137,138]. At atmospheric pressure, Pd(OH)_2_/C and H_2_ produce compound (**207**). This intermediate is then used to make a range of amides (**208(a,b)**) using different carboxylic acids. Following the hydrolysis of the phosphonic ester using McKenna’s technique and TMSBr, (**208(a,b)**) underwent simultaneous Boc deprotection. Nevertheless, the target compounds (**209(a,b)**) underwent undesired *N*-alkylation as a result of ethyl bromide liberation. The final compounds were transformed to their hydrochloric salts (**209(a,b)**) to improve the aqueous solubility. The majority of the compounds exhibited inhibitory effects against metallo-lactamases, with (**209b)** demonstrating the highest activity (IC_50_ = 4.1 mM, VIM-2) (Scheme 39) [139].

### 5.15. 8-Thioquinoline Conjugates

**Scheme 40:**
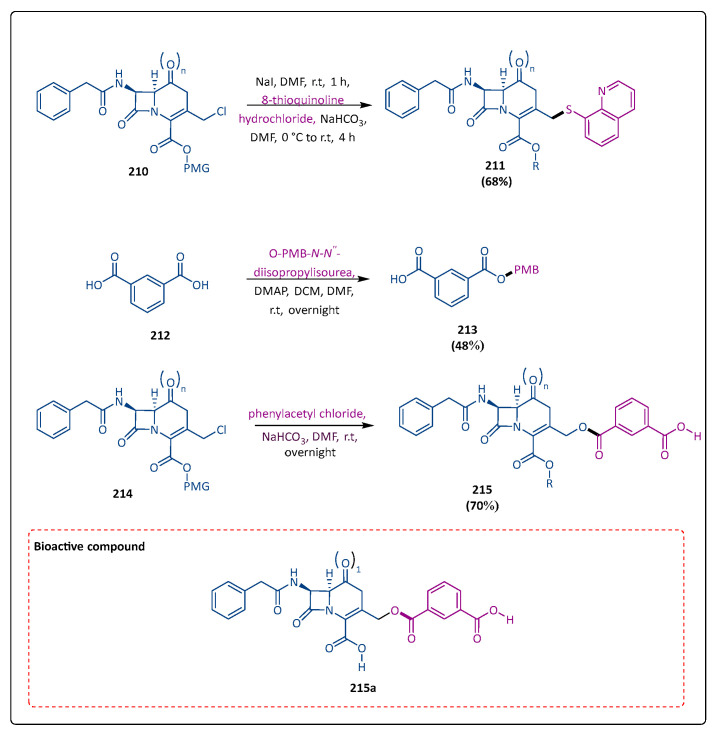
Synthesis of 8-thioquinoline conjugate derivatives.

One concerning development in antibiotic resistance is the rise in the frequency of bacteria expressing metallo-*β*-lactamases (MBLs). Because MBLs’ hydrolytic activities depend on active-site zinc ions, zinc chelators have been studied in the search for inhibitors of MBLs. Haren et al. produced a series of cephalosporin prodrugs comprising dipicolinic acid (DPA) and 8-isoquinoline (8-TQ), two strong zinc binders. The 8-TQ and DPA cephalosporin prodrug conjugates were synthesized by tying both substances to the 7-phenacyl cephalo-scaffold. To prepare the 8-TQ conjugates, compound (**210**) reacted with 8-thioquinoline hydrochloride in the presence of DMF, where NaHCO_3_ was used as a base, to form compound (**211**). Compound (**212**) reacted with O-PMB-*N-N*″-diisopropylisourea in the presence of DCM/DMF and DMAP as a coupling reagent to form compound (**213**). The ester connection between DPA and the cephalosporin core was broken; nevertheless, when the ester-linked conjugate (**214**) was attempted to be deprotected under identical conditions as those for compound (**215**), the IC_50_ values for the DPA and conjugate sulfoxide (**215**) against NDM-1 and VIM-2, respectively, were 0.299 μM and 0.756 μM, indicating nearly ten times more activity (Scheme 40) [140].

### 5.16. Dipicolinic Acid Isosteres

**Scheme 41:**
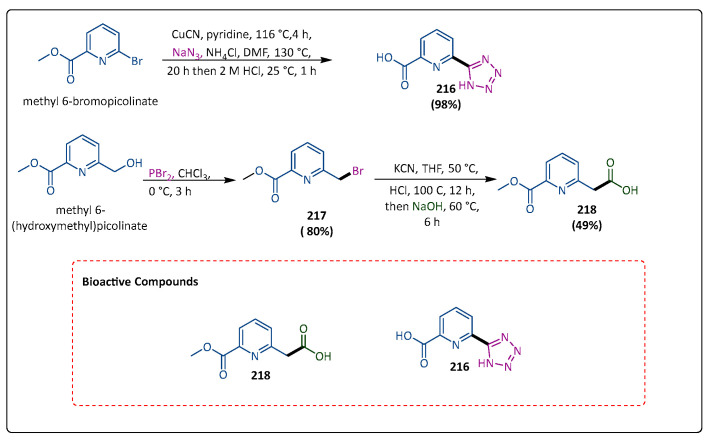
Synthesis of isosteres.

The *β*-lactam antibiotics, which are the most extensively prescribed and effective class of medicine, are severely threatened by New Metallo *β*-lactamase-1 (NDM-1). There are no inhibitors that are clinically significant to treat NDM. Most of the >500 inhibitors that have been identified so far, which are based on carboxylic acid sequences and the NDM-1 active site, bind Zn^2^ ions. Chen et al. synthesized dipicolinic acid (DPA) isosteres with strong inhibitory action against NDM-1 (as well as related metallo-*β*-lactamases IMP-1 and VIM-2) by isosterically replacing one carboxylate group of DPA. The mode of action and effectiveness of the NDM-1 inhibition were found to be influenced by the carboxylate isostere selection. Isostere (**216**) was achieved by the conversion of methyl 6-bromopicolinate to a nitrile via the Rosenmund–von Braun reaction, followed by an azide–nitrile cycloaddition. Briefly, compound (**217**) was obtained from commercially available methyl 6-(hydroxymethyl)picolinate. The substitution of the alkyl bromide of compound (**217**) with a nucleophile, followed by hydrolysis yielded isostere (**218**). Higher IC_50_ values (7.7:0.6 and 7.0:0.5 mm, respectively) were found for compounds (**216**) and (**218**). The parent DPA fragment was found to have the same tendencies as the IC_50_ values for meropenem when it came to the inhibition of NDM-1 by fluorocillin, of these five compounds. The IC_50_ values for compounds (**216**) and (**218**) were greater (Scheme 41) [141].

**Scheme 42:**
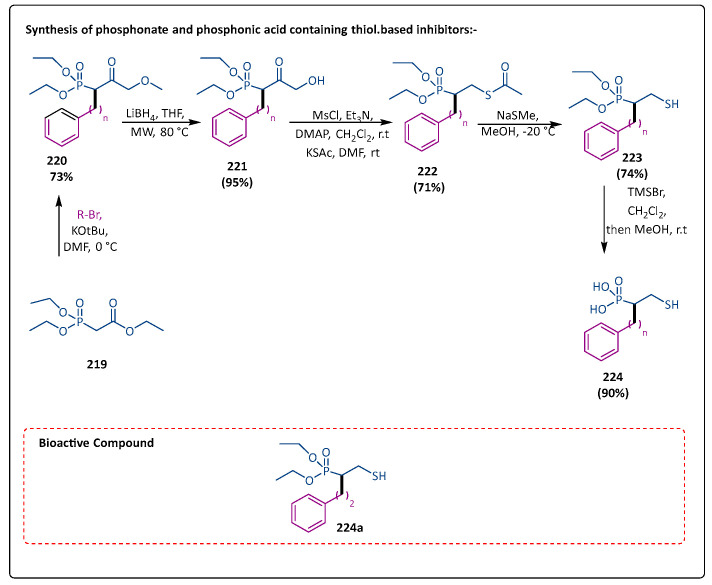
Synthesis of a series of bioisosteres.

The effectiveness of carbapenems and other *β*-lactam antibiotics is diminished by bacterial resistance. The primary mechanism of resistance against *β*-lactams is the hydrolysis of the *β*-lactam ring by serine- or metallo-*β*-lactamases (MBLs). One of the most well-known scaffolds linked to MBL inhibition is mercaptocarboxylic acid. Skagseth et al. synthesized several bioisosteres of previously published inhibitors and assessed them against the MBLs VIM-2, NDM-1, and GIM. In this study, bioisosteric groups, such as phosphonic acids, NH–tetrazoles, and phosphonate esters, were employed instead of mercaptocarboxylic acids’ carboxylate groups. It was assessed how the replacement affected inhibitor binding and bioactivity. Compound (**219**) was alkylated using KOtBu as base to afford the monoalkylated acetates (**220**) in moderate yields. The chemoselective reduction of the ester in the presence of the phosphonate was obtained with lithium borohydride to provide the corresponding alcohols (**221**). Subsequent mesylation followed by substitution with potassium thioacetate gave the thioacetates (**222**). Several methods for the deprotection of the thioacetates were evaluated [142,143,144]. The best results were obtained by treatment with NaSMe, providing the free thiols (**223**), which reacted with TMSBr in the presence of methanol to form (**224**). It was not possible to identify a broad-spectrum inhibitor that would target VIM-2, NDM-1, and GIM-1; nevertheless, thioacetate phosphonic acid (**224a**) appears to target both NDM-1 and VIM-2. Therefore, a structure-guided approach to designing an inhibitor that targets both VIM-2 and NDM-1 at the same time can benefit greatly from the published VIM-2 (Scheme 42) [145].

### 5.17. 1,2-Benzisothiazol-3(2H)-one Derivatives

**Scheme 43:**
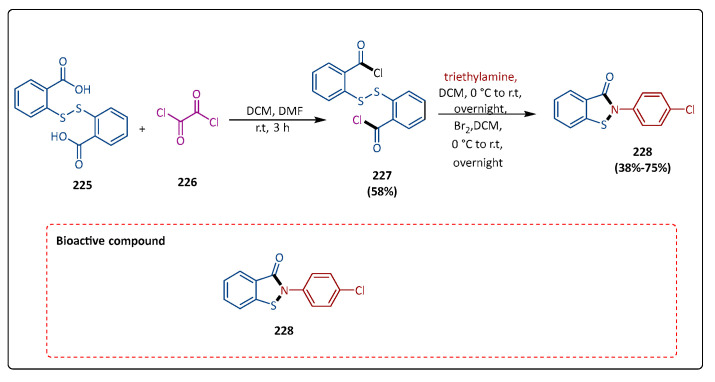
Synthesis of 1,2-benzisothiazol-3(2H)-one derivative.

Because of its hydrophobic amino acid residues and extremely flexible loops at the active site, New Delhi metallo-*β*-lactamase (NDM-1) is a potent *β*-lactamase that produces a fairly widespread and shallow drug-binding pocket that can accommodate and hydrolyze a wide spectrum of substrates. Jin et al. designed, synthesized, and characterized compounds to evaluate the synergistic activity and cytotoxicity of a small chemical library that included meropenem and related compounds, along with the 2-substituted 1,2-benzothiazole-3 (2H)-one derivative, against the strain of *E. Coli* Tg1 (NDM-1). Using a catalytic amount of dimethylformamide (DMF) and oxalyl chloride, compound (**225**) reacted with compound (**226**) to yield (**227**). The desired 2-substituted 1,2-benzothiazole-3 (2H)-one analog (**228**) was effectively generated by the intramolecular cyclization of the diamide under the molecular catalysis of bromine in the presence of dichloromethane (DCM) as a solvent, after amidation with a variety of primary aryl and alkyl amines. Compound (**228**) showed the greatest synergistic efficacy with Mem as well as acceptable tolerability. According to docking, compound (**228**) is predicted to form hydrogen-bonding interactions with Asp120 and hydroxide ions and a strong π-π-stacking interaction between its 4-chlorophenyl ring and His263 imidazole ring, aligning with SAR studies. Compound (**228**) demonstrated the highest potency, with an IC_50_ value of 0.44 ± 0.02 μM. It inhibited NDM-1 in a non-competitive and covalent manner, presenting a promising route for future research and possible chemical scaffolds (Scheme 43) [146].

### 5.18. N-Sulfamoylpyrrole-2-Carboxylates

**Scheme 44:**
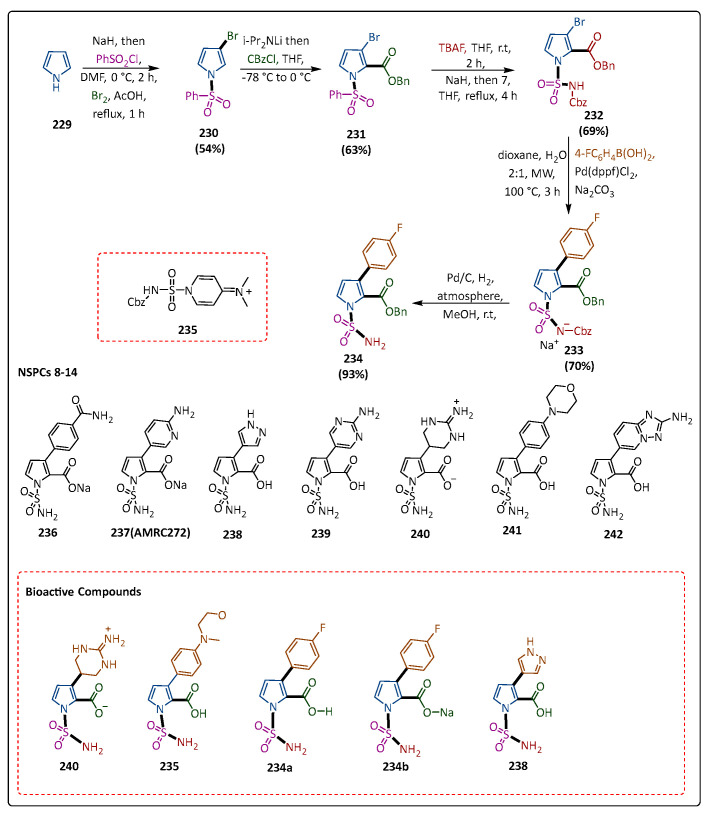
Synthesis of *N*-sulfamoylpyrrole-2-carboxylates.

Among the most significant classes of antibacterial medications is the *β*-lactam family; nonetheless, resistance, especially from *β*-lactamases, is steadily reducing their effectiveness. MBLAs are capable for efficiently breaking down all the *β*-lactam antibiotics, except monobactams. Although avibactam and clavulanic acid are examples of serine-*β*-lactamase (SBL) inhibitors that are clinically beneficial, no such MBL inhibitors are presently available on the market. Using strong inhibitors of clinically relevant B1-subclass MBLs, such as NDM-1, Farley et al. synthesized and studied the mechanism of action of *N-*sulfamoylpyrrole-2-carboxylates (NSPCs). From compound (**229**), the effective synthesis of compound (**252**) was easily accomplished in seven steps. PhSO_2_Cl is used to perform an initial *N*-sulfonylation of compound (**230**) through regioselective electrophilic C3-bromination with Br_2_. Compound (**231**) is obtained by electrophilic entrapment with benzyl chloroformate (CbzCl) and subsequent direct *ortho*-metalation via tetrabutylammonium fluoride (TBAF)-mediated *N*-sulfonyl deprotection, sodium-hydroxide-mediated pyrrole NH deprotonation, and electrophilic trapping with compound (**232**). The compound (**233**) was obtained as a sodium sulfonylazanide salt via Pd-catalyzed Suzuki–Miyaura cross-coupling with 4-fluorophenyl boronic acid. Sodium carboxylate (**233b**) (93%) was the product for hydrogenating this salt. Using aqueous HCl for acidification and Pd, C, and H_2_ for global deprotection, (**232**) is converted to the free acid (**233a**). An extremely effective VIM-1 inhibitor (pIC_50_ = 8.5), cyclic guanidine is comparable in potency to taniborbactam, a bicyclic boronate (formerly known as VNRX-5133), which is undergoing Phase III clinical studies [21]. A submicromolar activity (IC_50_) was demonstrated by the majority of the NSPCs for the inhibitions of VIM-2 and NDM-1, whereas **254**, **257a**, **257b**, **261,** and **263** demonstrated nanomolar potencies against NDM^−1^ (Scheme 44) [147].

### 5.19. 1H-Imidazole-2-Carboxylic Acid

**Scheme 45:**
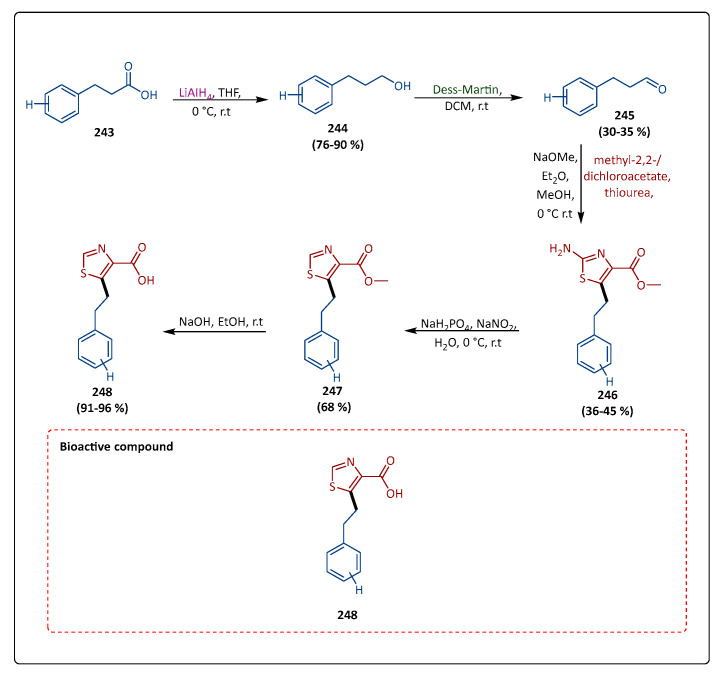
Synthesis of 1H-imidazole-2-carboxylic acid.

The world has been paying more and more attention to metallo-*β*-lactamases, or MBLs. Except for monobactams, ambler subclass B1 MBLs can hydrolyze nearly all the *β*-lactams, making them the most therapeutically relevant. Clinically helpful medications to treat MBL-mediated resistance are still lacking, nevertheless. Li et al. reported on the structural optimization of substituents and 1*H*-imidazole-2-carboxylic acid. The results of the structure–activity relation (SAR) analysis indicated that substituting other structurally highly comparable MBPs for thiazole-4-carboxylic acid or for 1*H*-imidazole-2-carboxylic acid lowered the MBL inhibition. After being used as a hydrogen source, compound (**243**) was reduced to the corresponding compound (**244**), which then oxidized at room temperature to produce compound (**245**). After that, target compound (**247**) was easily synthesized from various aldehydes by employing a procedure similar to that of target compound (**248**). Compound **248**’s molecular docking simulations predict binding modes with VIM-2, forming coordination and hydrogen-bonding interactions with Zn^2^ and Arg228 and enhancing VIM-2 inhibition through substituent L3 and L10 loop interactions. About IMP-1 (IC_50_ = 2.92 μM), VIM-1 (IC_50_ = 0.42 μM), VIM-2 (IC_50_ = 0.22 μM), and VIM-5 (IC_50_ = 0.43 μM), compound (**248**), with an unsubstituted phenyl ring, exhibited strong inhibition, whereas NDM-1 (IC_50_ = 82.36 μM) showed moderate inhibition (Scheme 45) [148].

**Scheme 46:**
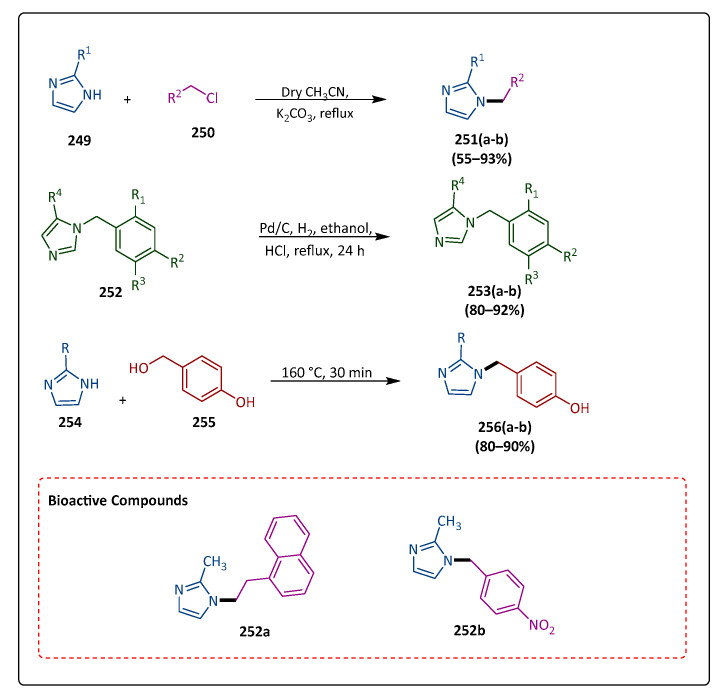
Synthesis of imidazole derivatives.

The development of metallo-*β*-lactamase enzymes, which can open and inactivate the lactam ring of *β*-lactam antibiotics, can lead to antibiotic resistance in bacteria. Metallo-*β*-lactamases (MBLs) do not yet have any known therapeutically accessible inhibitors, and pharmacological inhibitors of serine-*β*-lactamase are ineffective against MBLs. Arjomandi et al. produced mostly imidazole compounds, and their inhibitory actions were assessed. Later, the structure and arrangement of the ligands at the enzyme’s active site were predicted using an in silico binding model. Salvio et al.‘s [149] general methodology was used to generate compounds (**251(a,b))**. Using Pd/C, HCl, and H_2_ in ethanol, compounds (**353(a,b))** are synthesized in a single step according to the general protocol [150]. These compounds have a yield range of 80–92% and are synthesized in a single process. Compounds (**256(a,b))**, having good yields, were synthesized by following the general protocol described by Vallée et al. [151]. To achieve a yield range of 80–90%, the initial material mixture is heated to 160 °C for 30 min without the addition of a solvent. With an IC_50_ of 39 μM, compound (**252a**) exhibited the strongest inhibitory activity. Compound (**252b**) is the second most powerful enzyme-based inhibitor, with an IC_50_ of 46 μM (Scheme 46) [152].

### 5.20. Pyridine Derivatives

**Scheme 47:**
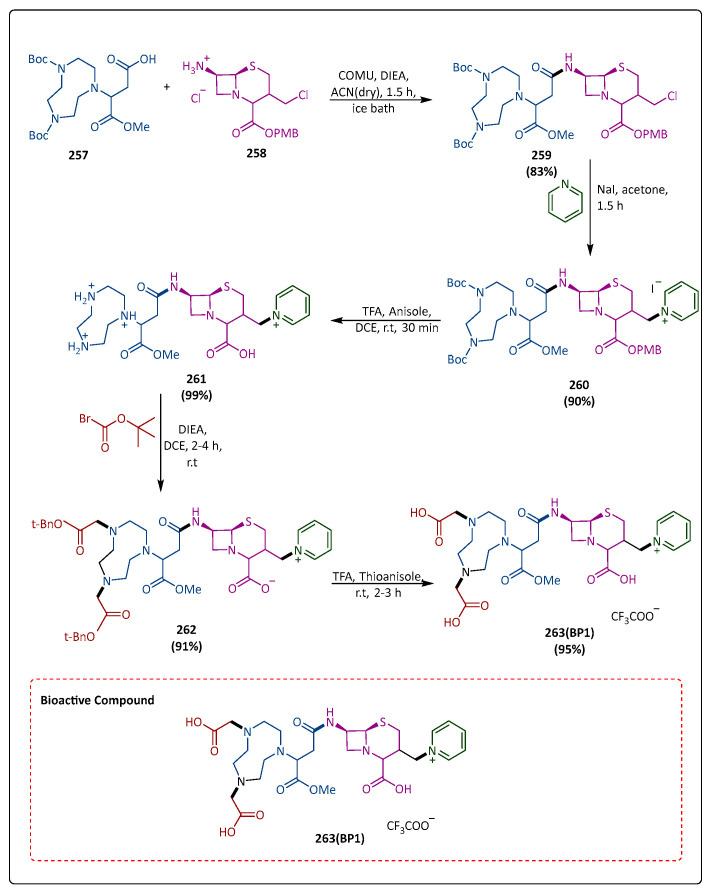
Synthesis of BP1.

The most frequently given class of medications is *β*-lactam antibiotic drugs because of their effective and extensive antibacterial capabilities. Nevertheless, alarming proportions of antibiotic resistance are now compromising these drugs’ efficacy in therapeutic situations, especially for *Enterobacterales* that produce metallo-*β*-lactamases and have carbapenem resistance. Peters et al. developed an MBL inhibitor, BL derived, to help MBL-expressing carbapenem-resistant *Enterobacterales* regain their meropenem minimum inhibitory concentration (MIC). The compound (**257**) reacted with compound (**258**) in diisopropylethylamine, utilizing the peptide coupling agent COMU to give compound (**259**), which reacted with pyridine to give compound (**260**). After Boc deprotection, pyridine was the leaving group in compound (**260**), at the 3-position, to generate compound (**261**), which was isolated in a 99% yield. The compound (**261**) was then alkylated to give compound (**262**), and the protecting moieties were then removed using TFA to yield the final desired compound (**263**) (BP1). A computational analysis validated the theory that BP1 binds to Zn^2+^ ions to inhibit MBLs. All things considered, research indicated that **263** (BP1) is a potentially effective MBL inhibitor that is safe for hepatocarcinoma cells in humans (IC_50_ > 1000 mg/L) and has efficacies of (Kiapp) 97.4 and 24.8 μM against VIM-2 (Verona integron-encoded MBL) and NDM-1 (New Delhi metallo-*β*-lactamase) (Scheme 47) [153].

**Scheme 48:**
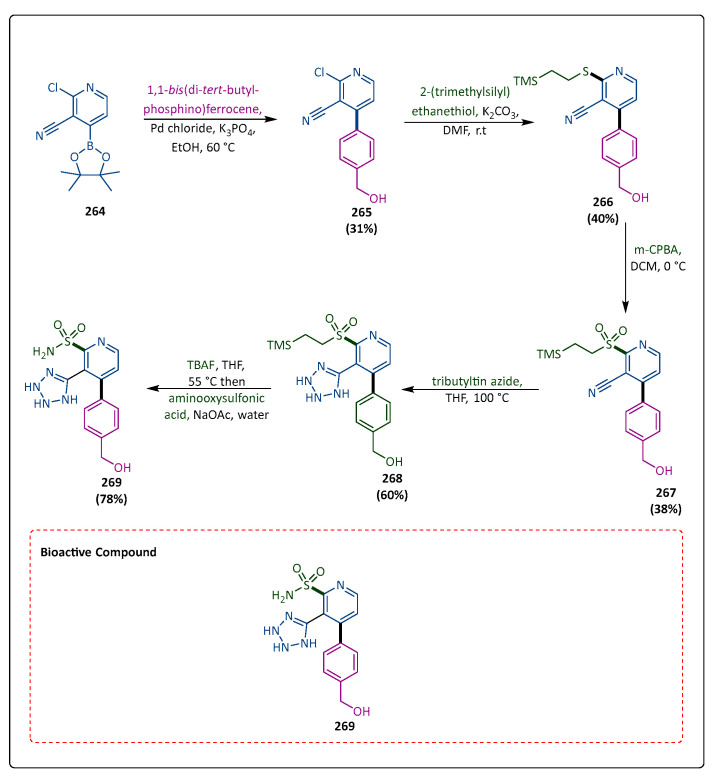
Synthesis of 4-pyridine sulfonamide analogs.

With NDM-1 spreading quickly and clinically relevant VIM-1 and IMP-1 present, our fight against bacterial infection has become more and more dependent on finding pan-inhibitors that target metallo-beta-lactamases (MBLs). To this end, we conducted a survey of MBLIs in conjunction with our functional moiety and large-scale screenings to indicate early medicinal chemistry engagement. Mandal et al. developed the first evaluation of the compounds and found them through a literature search and virtual screening. These efforts served as a basis for our concurrent hypothesis-driven structure-based drug design and SAR investigation. Before the transfer of the α-chlorine to the pyridine N_2_ by 2-(trimethylsilyl)-ethanethiol, the biaryl moiety was installed to form compound (**266**). The resulting compound (**267**) was oxidized and reacted with tributyltin azide to provide compound (**268**). Ultimately, compound (**269**) was produced by treating the sulfone molecule of compound (**269**) sequentially with tetrabutylammonium fluoride and then aminooxysulfonic acid, converting it to sulfonamide. Compound (**269**) was the driving force behind the development of the MBLI clinical candidates after it was shown in vivo experiments to effectively reduce the bacterial burden in a mouse infection model when combined with imipenem (IPM) (Scheme 48) [154].

### 5.21. Metal Chelators

**Scheme 49:**
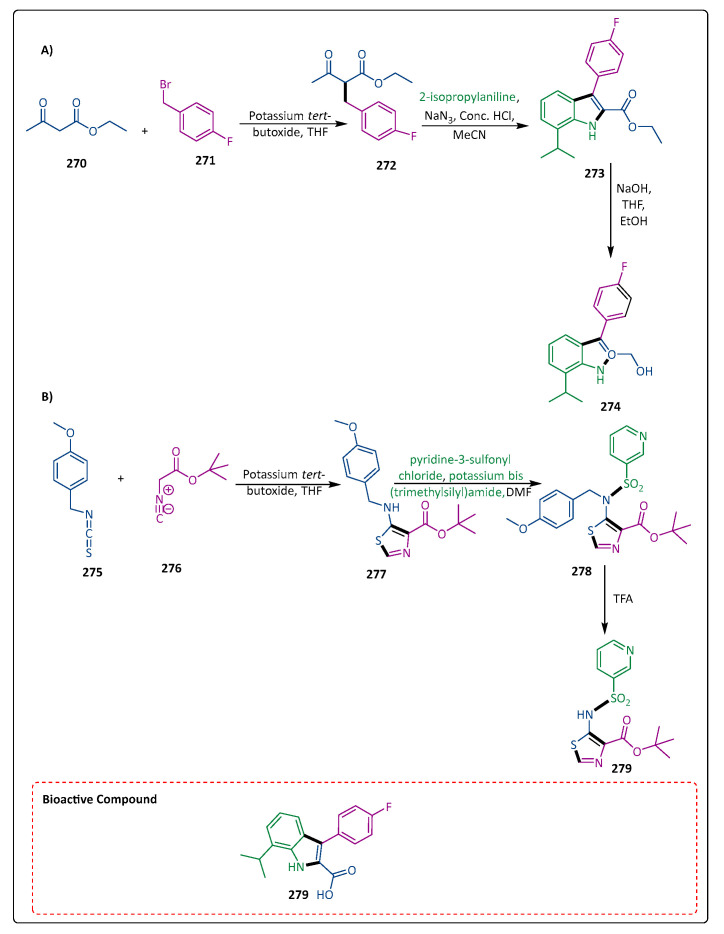
Synthesis of metal chelators.

Bacterial enzymes known as MBLs (metallo-*β*-lactamases) depend on zinc to make the majority of the *β*-lactam antibiotic classes not in use, covering the last-choice carbapenems. Because there are currently no inhibitors of MBLs with clinical approval, agents must comprehend the inhibitory mechanisms of their medicines. Wade et al. described a thorough mechanistic study of several structurally different MBL inhibitors that have been documented in patents and the scientific literature. They particularly determine the maximum IC_50_ against MBLs of each inhibitor that are members of the IMP and NDM families. Compound (**297**) was prepared according to Scheme A. Briefly, compound (**270**) reacted with compound (**271**) to form compound (**272**), which reacted with 2-isopropyaniline via in situ diazotization to form compound (**273**) by acidic cyclization. Chemical (**274**) was then generated by ester hydrolysis. Following a reaction with pyridine-3-sulfonyl chloride, compound (**275**) reacted with compound (**276**) to generate compound (**277**), which then reacted with pyridine-3-sulfonyl chloride to yield compound (**278**). Compound (**279**) is produced in a quantitative yield by sulfonamide synthesis, which is followed by deprotection under TFA in Scheme B. With IC_50_ values in the nanomolar range, the indole carboxylate derivative (**279**) showed very strong actions against NDM-1 and IMP-1 (Scheme 49) [155].

**Scheme 50:**
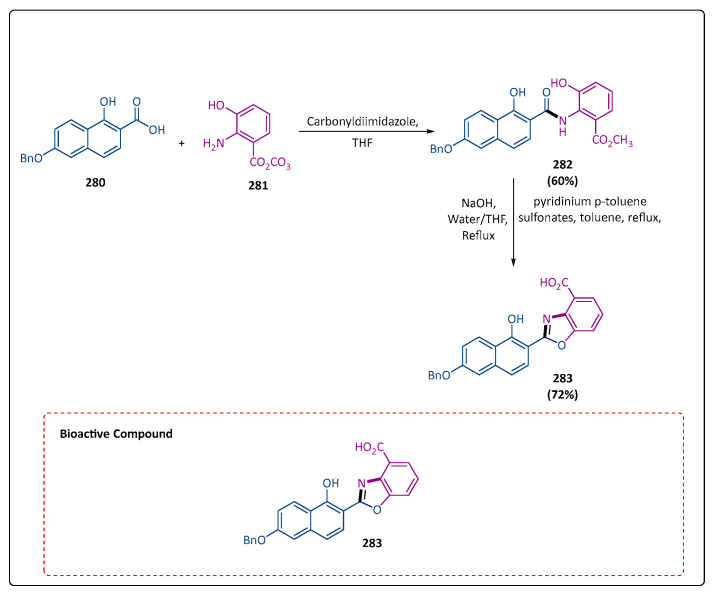
Synthesis of benzoxazole and benzimidazole–zinc chelator.

NDM-1, as a metallo-*β*-lactamase, employs catalytic zinc ions within its active sites to break down nearly all the commercially accessible *β*-lactam antibiotics. The development of inhibitors for metallo-*β*-lactamases is imperative to combat this resistance mechanism. For inhibition development, zinc-binding compounds are appealing candidates because they are found in several NDM-1 inhibitors, along with pharmacophores that bind zinc. Jackson et al. reported zinc chelators, including benzoxazole and imidazole, to exhibit an inhibition against NDM-1, a metallo-*β*-lactamase of therapeutic significance. The compound (**282**) was generated through the reaction of compounds (**280**) and (**281**) with the acid-catalyzed cyclodehydration of the amide molecule and the hydrolysis of the ester, which resulted in the formation of the benzoxazole ring to give compound (**283**). With an IC_50_ of 0.38 μM, inhibitor compound (**283**) had the highest efficacy and offered encouraging prospects for the formation of NDM-1 inhibitors (Scheme 50) [156].

**Scheme 51:**
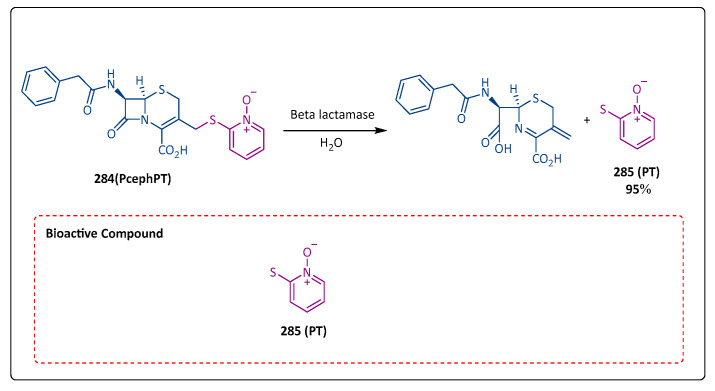
Synthesis of prochelator PcephPT.

The production of *β*-lactamases has a role in the rapidly growing clinical issue of antibiotic resistance. The increasing frequency of MBLs brings a major hurdle because currently, known inhibitors of *β*-lactamases work averse to the active sites of serine-*β*-lactamases but not against the zinc-containing active sites of MBLs. Jackson et al. studied an inhibitor of New Delhi metallo-*β*-lactamase 1 (NDM-1), the antibacterial chelator-releasing prodrug PcephPT (2-((6*R*,7*R*)), 8-oxo-7-(2-phenyl acetamido) -2-carboxy oct-2-en-3-yl) methyl) thio) pyridine 1-oxide) as -5-thia-1-aza bicyclo [4.2.0], which cephalosporin core releases a metal-chelating agent when it reacts with the enzymes cephalosporinase and carbapenemase. PT (**285**) was hydrolyzed by PcephPT (**284**). Pyrithione (**285**) (PT), a well-researched antibacterial molecule with a metal-chelating bidentate framework, is the releasing group of the PcephPT. PT (**285**) can cause the hyperaccumulation of metals, particularly copper, and disrupt metal homeostasis (Scheme 51) [157].

**Scheme 52:**
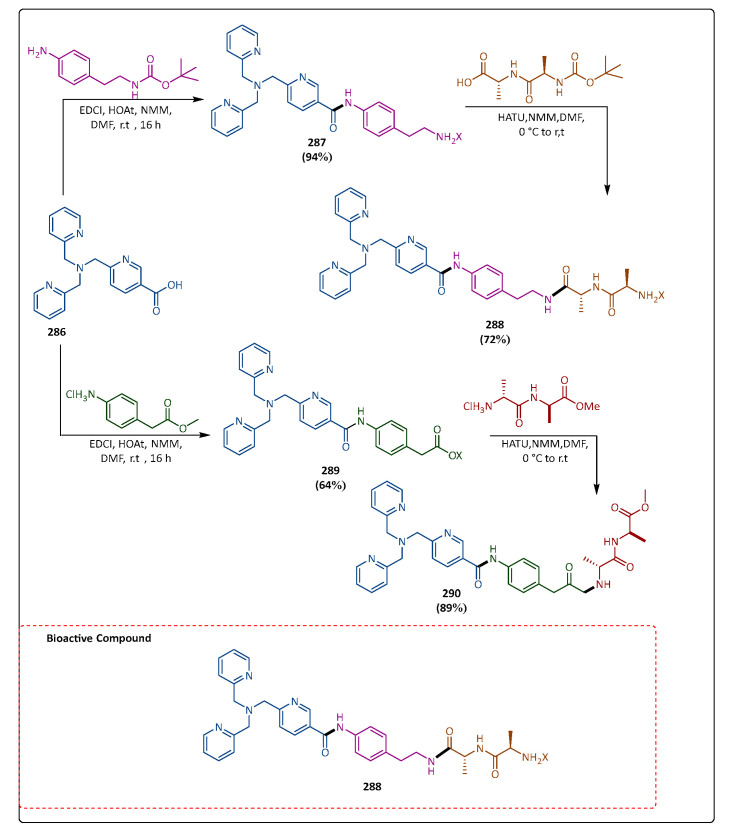
Synthesis of zinc chelator *tris*-picolylamine (TPA).

The need for efficient and accessible MBL inhibitors for therapeutic usage stems from the global rise in AMR (antimicrobial resistance) and the growth of organisms expressing drug-resistant MBLs. Research in this field is urgently needed to advance, as there is currently no effective inhibitor based on tris-polyamine (TPA), a selective zinc chelator. Schnaars et al. designed the synthesis, biological evaluation, and development of chemical compounds that can revive the bactericidal activities of MEM (meropenem) against *Klebsiella pneumoniae* and *Pseudomonas aeruginosa*, which express carbapenemases, New Delhi MBL 1 (NDM-1), and Verona integron-encoded MBL (VIM-2). Compounds (**287**) and (**288**) were synthesized by combining the appropriately protected anilines with EDCI and then deprotecting the resulting compounds with either LiOH or TFA. Following further deprotection, the resulting C- and N-terminal TPA-linker fragments were coupled by HATU with Boc-D-ala-D-ala-OH or H-D-ala-D-ala-OMe, yielding corresponding compounds (**289**) and (**290**). Among the best compounds, (**288**) reduced the MEM MIC at 50 μM by a factor of 32–256 in all the analyzed MBLs exhibiting clinical isolates that did not exhibit any activity against serine-carbapenemase-expressing isolates. Utilizing pure VIM-2 and NDM-1 and (**288**) biochemical assays, the inhibitory kinetics, with K_i_ values of 12.5 min-1/0.500 min-1 mM-1, were determined (Scheme 52) [158].

### 5.22. Phosphonamidate Monoesters

**Scheme 53:**
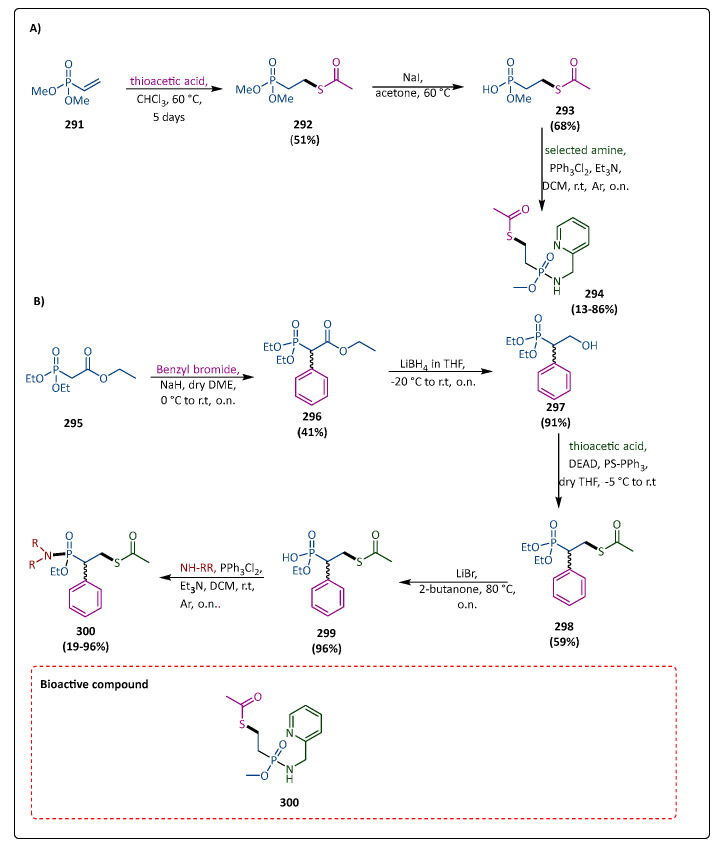
Synthesis of phosphonamidate monoesters.

Most commonly, resistance develops in bacteria through the degradation of the present family of *β*-lactam antibiotic drugs. It is expected that the suppression of MBLs will allow for the ongoing use of the present antibiotic drugs, which range is reducing. Palica et al. reported the results of phosphonamidate monoesters’ cytotoxicity tests and the NMR spectroscopic identification of their protein-binding sites. They also established an effective synthetic approach to synthesize putative phosphonamidate inhibitors of MBLs. Phosphonamidate (**294**) is generated when dimethyl vinyl phosphonate (**291**) is reacted with thioacetic acid to produce a new phosphonic ester (**292**). This ester then reacted with sodium iodide to produce monophosphonic acid (**293**) in scheme section A. Triethyl phosphonoacetate (**295**) combined with benzyl bromide in NaH and 1,2-dimethoxyethane (DME) to form (**296**). Then, compound (**296**) was reduced to the corresponding alcohol with LiBH_4_ to give compound (**297**). The Mitsunobu reaction, using immobilized triphenylphosphine, provided the thioester (**298**). Analogous to the first synthetic pathway, compounds (**300**) were obtained in 19–96% yields by the hydrolysis of the phosphonic ester (**298**) to the monoacid (**299**) using LiBr (96%), followed by phosphonamidation in scheme section B. A molecular docking study suggested that the compound (**300**) bound to NDM-1 at the hydrophobic substrate’s binding site, likely driven by hydrophobic interactions. Further optimization could lead to phosphonamidate-mimicking MBL inhibitors. The most potent substance toward GIM-1 (**300**), undergoes deacetylation of its thioester (Scheme 53) [159].

### 5.23. New Thiazole Thioacetamide Derivatives

**Scheme 54:**
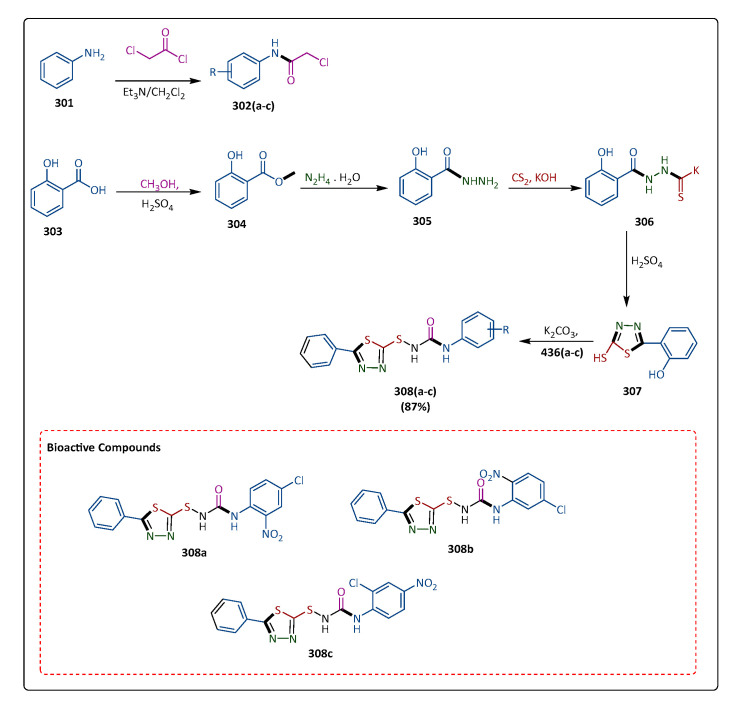
Synthesis of new thiazole thioacetamide derivatives.

Antibiotic resistance caused by M*β*Ls poses a substantial danger to the management of bacterial diseases. Sulfur-containing moieties are important in the creation of M*β*L inhibitors, as the sulfur atom can connect to zinc ions, the enzyme’s active sites, and replace the bridging water molecules, thus lowering the M*β*L activity [160,161]. Zhang et al. identified thioacetamides as possible M*β*L inhibitor skeletons. By altering the aromatic substituent, new thiazolethioacetamides were produced to enhance the skeletons’ information. *N*-substituted-2-chloroacetamides (**302(a–-c)**) were produced via the reaction of substituted anilines (**301**) with chloroacetyl chloride. By esterifying salicylic acid (**303**), methyl-2-hydroxybenzoate (**304**) is formed. It then condensed with hydrazine to form hydrazides (**305**). Under basic conditions, the hydrazides reacted with CS_2_ to form (**306**). After stirring with H_2_SO_4_ in an ice-water bath, compound (**307**) was produced, which subsequently reacted with K_2_CO_3_ to yield (**308(a-c)**). This study used molecular docking to examine inhibitor interactions, with binding energies of −6.97, −6.59, −12.64, and −8.14 kcal/mol for the VIM-2/8, VIM-2/12, CphA/5, and CphA/8 complexes, respectively, and found that the substituent location and the electron-withdrawing group have substantial impacts on thiazole thioacetamide’s inhibitory activity, which can help to further improve these compounds. Compounds (**308(a–c)**) exhibit greater efficacies as inhibitors of ImiS (Scheme 54) [162].

## 6. Dual Nature of *β*-Lactamase Inhibitors

Boron-containing compounds have demonstrated broad inhibitory effects on both MBLs and SBLs [22,163]. Developed early in the realm of boronic acid derivatives, vaborbactam is the first cyclic-boronate-based SBL inhibitor. The U.S. FDA has licensed it for use in conjunction with meropenem to treat adult patients with severe urinary tract infections [68]. Krajnc et al. described the synthesis of VNRX-5133. It inhibits a variety of SBLs and clinically relevant MBLs, comprising VIM-1/2 and NDM-1. VNRX-5133 was evaluated for B2/B3 MBLs and demonstrated either little or no effect against IMP-1 [21]. Furthermore, several other cyclic boronates, including taniborbactam, QPX7728, and RPX-7350, are now being developed as broad-spectrum MBL and SBL inhibitors in the preclinical or clinical stages [13,22,163,164].

### 6.1. 4-Amino-1,2,4-Triazole-3-Thione Derivatives

**Scheme 55:**
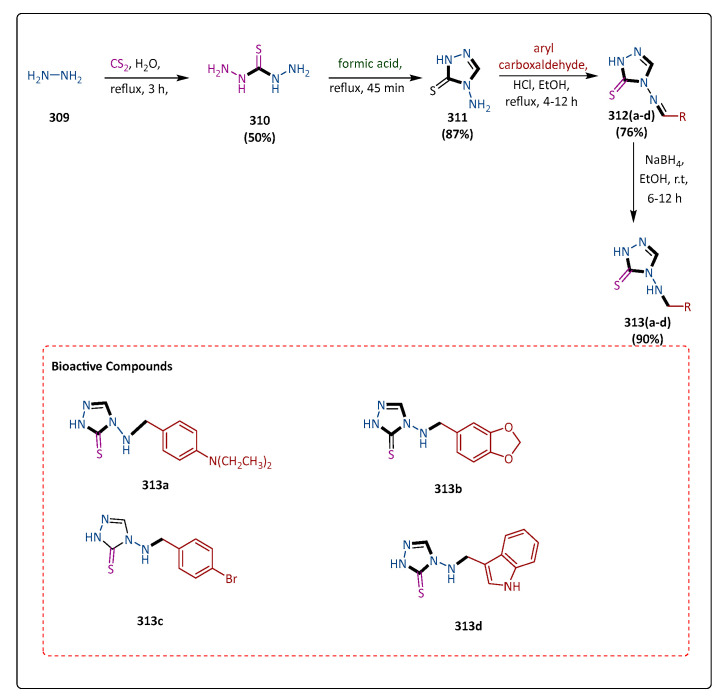
Synthesis of 4-amino-1,2,4-triazole-3-thione scaffold.

Gram-negative bacteria that are resistant to multiple drugs (MDR) are a recognized public health problem, as they are increasingly contributing to worldwide mortality rates and decreasing the effectiveness of treatments for bacterial infections [165]. The effectiveness of the contemporary arsenal of *β*-lactam antibiotics is at risk because of the appearance of bacteria that simultaneously express metallo- and serine carbapenemases. Linciano et al. discovered the 4-amino-1,2,4-triazole-3-thione functional framework and used it as the original chemical fragment to generate a small assembly of inhibitors of BLIs with a prolonged profile of biological significance. The produced compounds have undergone in vitro evaluation against class B1 metallo-*β*-lactamases (MBLs) VIM-1 and IMP-1, as well as the class A SBL KPC-2. The compound (**309**) and CS_2_ reacted to produce compound (**310**). The compound (**310**) reacted with formic acid to yield compound (**311**). Then, compound (**311**) reacted with aryl carboxyaldehde in refluxing ethanol and HCl to give compound (**312**). Finally, the reduction of the compound (**312**) with NaBH_4_ in 80% aqueous ethanol for 6–24 h led to the respective final compounds **313(a–d)** in good yields. Four compounds docked at the protein’s active site indicated thiotriazole candidates’ orientation in carbapenemases. Zinc ions at MBLs’ binding sites favor the thiol form and deprotonation, while the thione form is predominant in SBLs. Cross-class micromolar inhibitory potency was demonstrated by four of the synthesized derivatives “**313(a–d)**”. Consequently, in silico analyses were conducted to ascertain the mechanism by which these compounds bonded to the catalytic pockets of serine- and metallo-BLs (Scheme 55) [166].

### 6.2. Thioether-Substituted Bicyclic Boronate

**Scheme 56:**
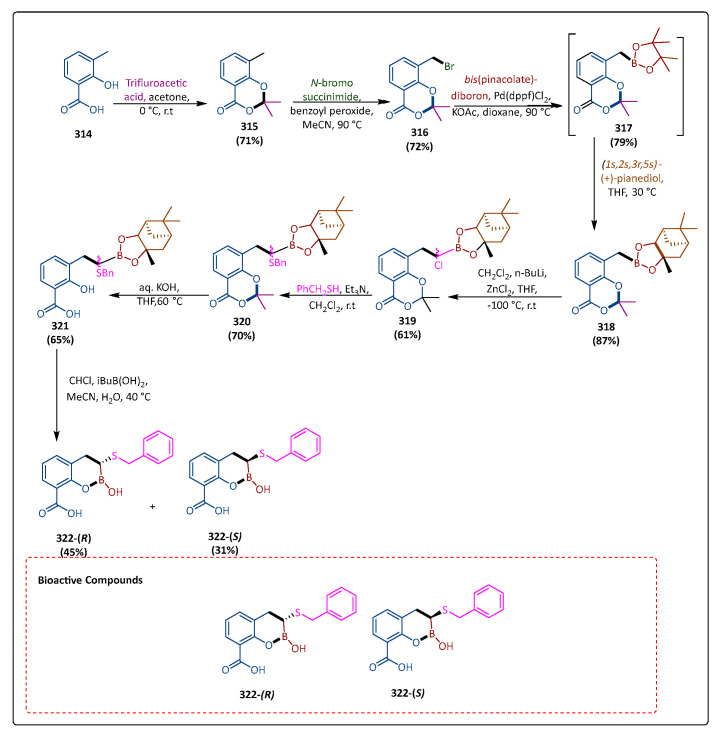
Synthesis of *C-*2 substituted bicyclic boronates.

*β*-Lactamases exhibit the predominant form of resistance against *β*-lactam antibiotics. A novel class of BLI cyclic boronates has shown promise in inhibiting SBLs and MBLs. *β*-Lactamases constitute a bicyclic boronate, which is the most prevalent form of resistance to *β*-lactam antibiotics. Cyclic boronates have shown promise in potently inhibiting SBLs and MBLs. Parkova et al. described a bicyclic boronate with a thioether side chain positioned analogously to the usual C-6/C-7 side chain of cephalosporins/penicillins as the subject of biochemical and biophysical research. The activities of the thioether bicyclic boronate ester against metallo- and serine-*β*-lactamases were evaluated; 3-methyl salicylic acid was the first stage in the eight-step stereoselective process used to generate the thioether-substituted cyclic boronate (**321**). The compound (**315**), produced by one-pot protection and benzoic acid groups in an acidic environment, subsequently underwent bromination through *N*-bromosuccinimide to generate compound (**316**) (72%). Then, the compound (**317**), produced via the Miyaura borylation of the bromide (**316**), underwent transesterification in situ using (1*S*,2*S*,3*R*,5*S*) -(+)-pinanediol, yielding a good yield (87% over two stages) of the boronic acid (+)-pinanediol ester (**317**). After that, boronate (**318**) underwent Matteson homologation [41,42], yielding compound (**319**) in the form of a mixture of stereoisomers (8*S*:8*R* 72:28, correspondingly). Compound (**320**) was obtained via nucleophilic substitution (assumed inversion) with benzyl mercaptan. The required compound (**322**) was obtained by hydrolyzing the compound (**321**) and cleaving the isopropylidene-protecting group. It was then separated as a mixture of (*R*/*S*)-enantiomers. On the other hand, All the studied *β*-lactamases were inhibited by compound (**322**); however, potency differences were seen, similar to those of published acylamino bicyclic boronates (Scheme 56) [70,167].

### 6.3. VNRX-5133

**Scheme 57:**
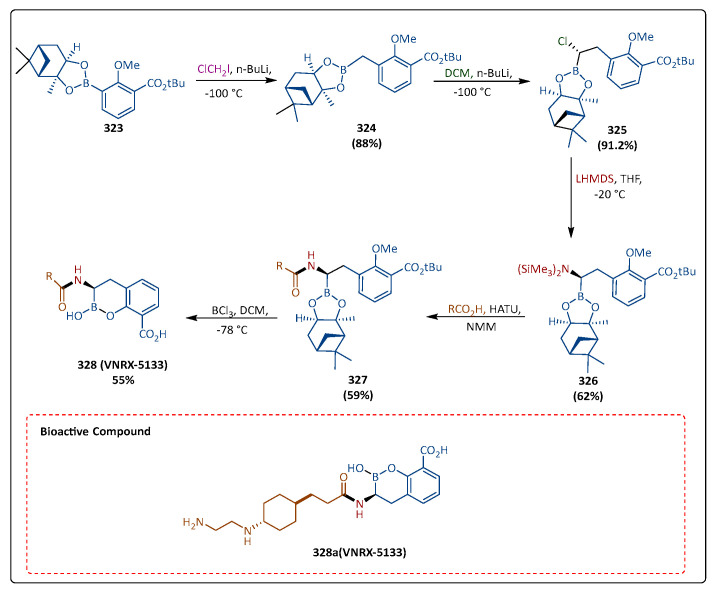
Synthesis of taniborbactam (VNRX-5133).

Taniborbactam, also known as VNRX-5133, is a pan-spectrum *β*-lactamase inhibitor that contains boronic acid. Research conducted in vitro*/*in vivo shows that the efficacy of *β*-lactam antibiotics was enhanced against *Enterobacteriaceae* and *Pseudomonas aeruginosa* that exhibit carbapenem resistance. Taniborbactam enters the clinical development stage as the initial pan-spectrum *β*-lactamase inhibitor. Utilizing microbiological profiling, biochemical testing, and structural biology. Liu et al. were able to identify taniborbactam, a very effective pan-spectrum BLI that has exceptional Gram-negative outer membrane penetration and inhibits *β*-lactamase enzymes belonging to all four ambler groups [21,22,99,163,168]. The compound (**323**) selectively reacted with (chloromethyl)lithium at a temperature below 100 °C to yield (**324**), which then reacted with dichloromethane to yield (*S*)-α-chloro-boronate (**325**). The intermediate compound (**326**) was then produced by the reaction of the chlorine atom of compound (**325**) with lithium *bis*(trimethylsilyl)amide at −20 °C. Then, compound (**326**) was treated in situ (NMM and HATU) with the acid to form compound (**327**). After being treated with BCl_3_ (1 M in DCM) at -78 °C, the resulting amides were deprotected and cyclized to produce crude boronates (**328**) in good yields. The increased permeability of the Gram-negative outer membrane, periplasmic accumulation, and broad-spectrum inhibition of *β*-lactamases were discovered to be dependent on the side chain *N-(*2-aminoethyl)-cyclohexylamine of (**328a**) (VNRX-5133) (Scheme 57) [22].

**Scheme 58:**
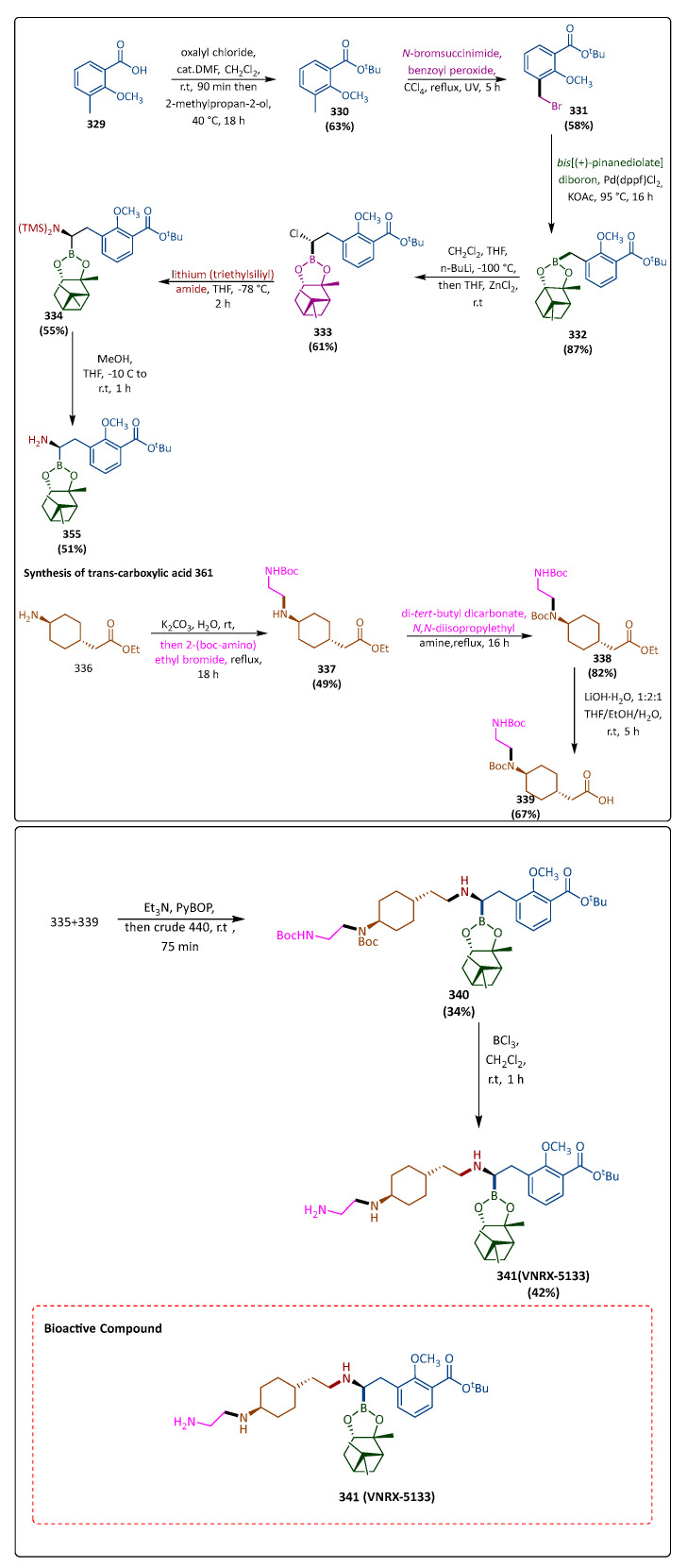
Synthesis of VNRX-5133.

The primary resistance mechanism for *β*-lactams, the most significant class of antibacterials, is *β*-lactamase-catalyzed hydrolysis. Nucleophilic SBLs, which are inhibitors of one of the two molecular groups of *β*-lactamases [169], are well-established medications that should be taken in conjunction with a suitable *β*-lactam antibiotic partner. Krajnc et al. reported the development of VNRX-5133, which inhibits various clinically significant MBLs, such as VIM-1/2, NDM-1, and SBLs. VNRX-5133 showed either negligible or no action contrary to IMP-1 and tested B2/B3 MBLs. Using a modified version of the reported [21] stereocontrolled method, VNRX-5133 was created in 11 steps via Matteson homologation [170] from compound (**329**). Compound (**329**) reacted with anhydrous 2-methylpropan-2-ol in anhydrous CH_2_Cl_2_ (20 mL) and oxalyl chloride to form compound (**330**), which reacted with *N*-bromosuccinimide in CCl_4_ and benzoyl peroxide to form compound (**331**). As per reported methods [168], the necessary (+)-pinanediol boronate precursor (**332**) (87%) was made. (*S*)-chloride compound (**333**) was produced via the stereoselective homologation of one carbon utilizing *in situ*-generated compound (**332**) [171]. At −90 °C, (**333**) reacted with lithium *bis*(trimethylsilyl)amide to produce compound (**334**), which had a configuration inversion. Compound (**335**) was produced using stoichiometric anhydrous methanol (TMH) from −10 to 25 °C to prevent decomposition with compound (**334**). The required carboxylic acid side chain with the trans stereochemistry (**339**) was produced from compound (**336**) in three steps. Using 5 mol% benzyl ammonium chloride as a phase-transfer catalyst under biphasic conditions (1:1, CH_2_Cl_2_: H_2_O) produced **337** (49%), which was then Boc-protected to produce compound (**338**) (82%). Ion-exchange chromatography (Amberlite H-120) after saponification produced (**339**). Using (benzotriazole-1-aryloxy)tripyrrolidinophosphonium hexafluorophosphate (PyBOP) [172], the amide connecting (**335**) and (**339**) was formed to give compound (**340**), yielding 34%. VNRX-5133 **(341)** (42%) was produced by a one-pot breakdown of the Boc, methyl ether, and tert-butyl-protecting groups, and the chiral auxiliary employing BCl_3_ (−78 °C, CH_2_Cl_2_) and an acidic workup (presumably to promote the bicyclization spontaneously). With especially strong activity, VNRX-5133 (**341**) exhibits submicromolar maximal-half IC_50_ values (0.53–0.008 μM) contemporary to all the main classes of therapeutically important *β*-lactamases examined (Scheme 58) [21].

### 6.4. 3-Aryl-Substituted Benzoxazole Derivatives

**Scheme 59:**
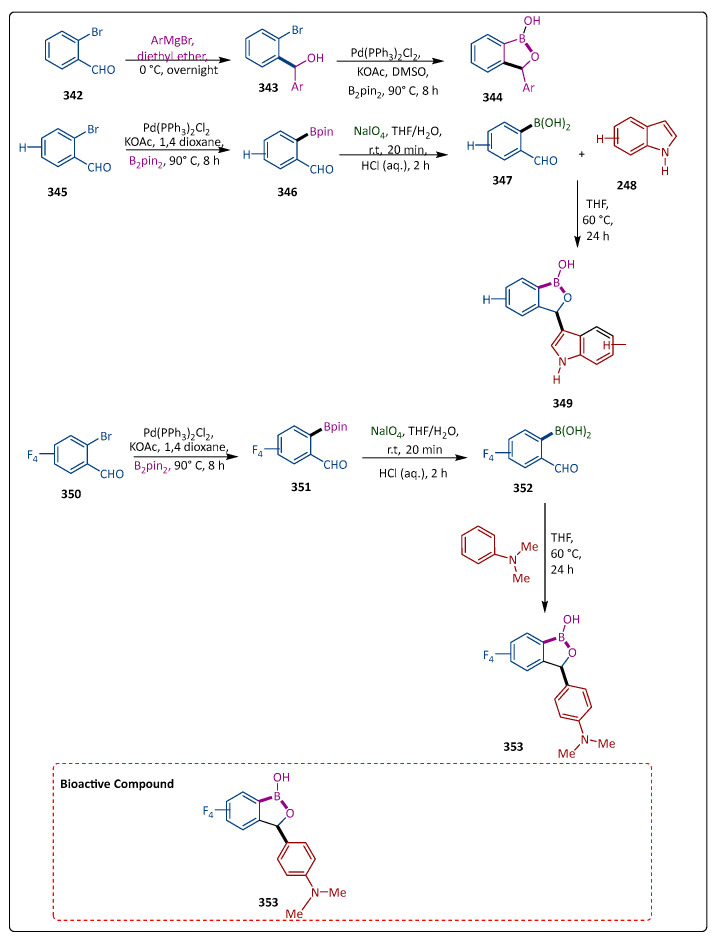
Synthesis of 3-aryl-substituted benzoxazole derivative.

The formation of *β*-lactamases is the major outcome of the resistance to therapeutically relevant *β*-lactam medicines. It has been demonstrated that boron-containing compounds have the potential to be utilized as broad-spectrum BLIs to combat *β*-lactam resistance. Yan et al. reported a group of 3-aryl-substituted benzoxazole analogs to exhibit broad-spectrum inhibition of typical metallo-*β*- and serine-*β*-lactamases (MBLs) and SBLs. The compound (**342**) reacted with the Grignard reagent to yield (**343**), which underwent a Miyaura borylation/cyclization cascade catalyzed by palladium to become the corresponding compound (**344**). However, (**345**) is employed in the synthesis of (**359**). First, (**346**) is accessible by borylation catalyzed by palladium. Using substituted indoles as nucleophiles, the pinacol ester is hydrolyzed to yield the corresponding analogs (**347**), from which the final products (**349**) are produced via a cyclization cascade through nucleophile (**348**) [173]. Using *N-*substituted anilines as nucleophiles, (**352**) and, in the same synthetic sequence, (**353**) were produced in excellent yields. Docking simulations were conducted to predict the binding modes of (**353**) with KPC-2 and NDM-1. The results showed that (**353**) likely forms covalent bonds with Ser70 in KPC-2 and hydrogen bonds with Asn170 and Glu166. With an IC_50_ value of 86 nM for the KPC-2 SBL and micromolar inhibitory potentials against other examined enzymes, (**353**) was the most effective inhibitor. Cell-based experiments further showed that (**353**) exhibited no discernible toxicity in HEK293T cells and could drastically lower meropenem’s MICs in clinically derived KPC-2-generating bacterial strains (Scheme 59) [174].

### 6.5. Azetidinimine Derivatives

**Scheme 60:**
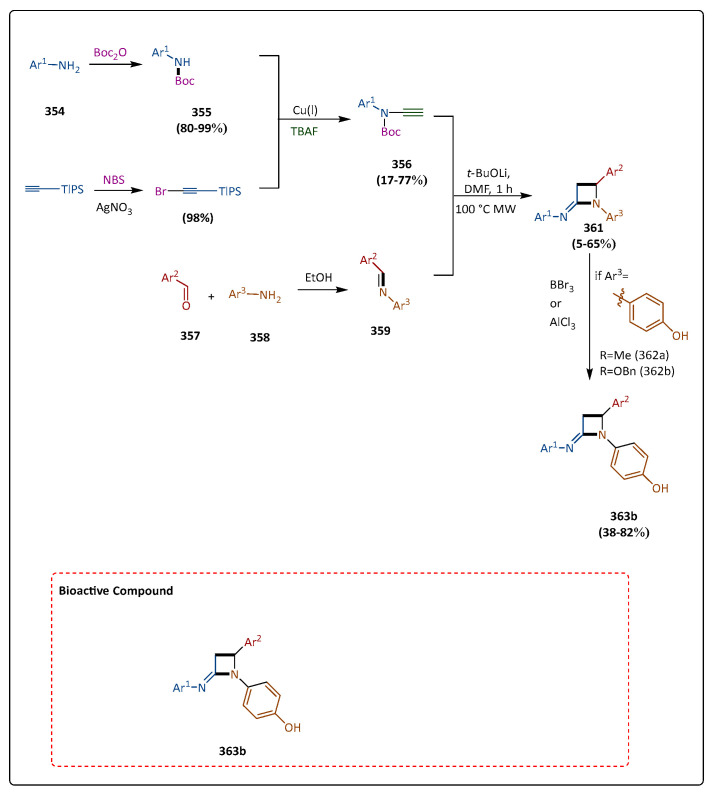
Synthesis of triarylated azetidinimines.

One of the main causes of the fast-increasing levels of resistance in Gram-negative bacteria is the broad dissemination of highly efficient *β*-lactamases, which render most *β*-lactam antibiotics as useless. Romero et al. reported the development of azetidinimines, a class of imino analogs of *β*-lactams that are potent non-covalent *β*-lactamase inhibitors. The Ar_1_ group originates from aniline (**354**), which is shielded by a Boc moiety to give (**355**). Following TBAF-promoted desilylation, coupling (**356**) with brominated triisopropylsilylacetylene yields dynamite (**357**). Imines (**360**) are produced when benzaldehyde (**358**) and aniline (**359**) condense. Using microwave heating, the crucial [2 ÷ 2] cycloaddition yields azetidinimines (**361**), but at the same time, the Boc protective group is lost. Through ether cleavage with AlCl_3_ (for OBn) or BBr_3_ (for OMe), compounds **362a** and **362b** were produced from compound (**363**), respectively. The docking of these enzymes with the two enantiomers of **363a** and **363b** was performed using GOLD, and the results were NDM-1(0.07 ± 0.02 μM), KPC-2 (0.07 ± 0.04 μM), and OXA-48 (0.28 ± 0.01μM). The **363b** enantiomers of the VIM variants interact with NDM-1 and VIM-1, unlike metallo-*β*-lactamase inhibitors. VIM-52’s activity loss may be because of its inability to access the active site. Overall, **363b** may be able to inhibit the three main therapeutically relevant carbapenemases of ambler classes A (KPC-2), B (NDM-1), and D (OXA-48), with K_i_ values below 0.3 mM, as well as the cephalosporins CMY-2 (class C), with 86% inhibition at 10 mM (Scheme 60) [175].

### 6.6. QPX7728

**Scheme 61:**
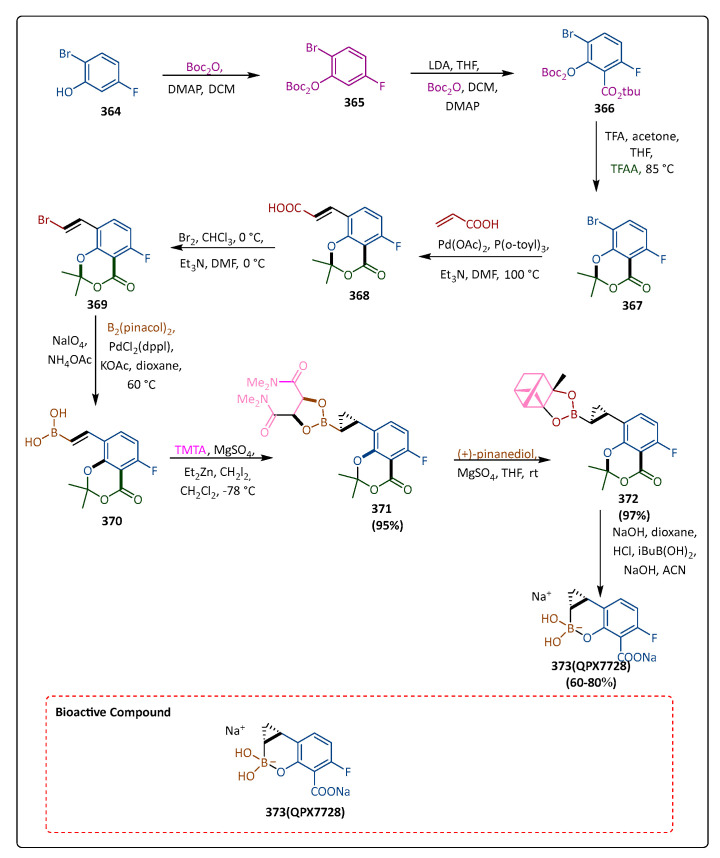
Synthesis of QPX7728.

The number of useful *β*-lactam therapy options [176] is severely limited because of the rapid evolution of multidrug-resistant strains and the rapid dissemination of bacterial resistance. It is commonly known that adding a *β*-lactamase inhibitor to *β*-lactams (ampicillin, ticarcillin, piperacillin, and amoxicillin) can increase and restore their activities. Boyer et al. synthesized the *β*-lactamase inhibitor QPX7728, which is scalable, highly yielding, and very selective, using nickel–boron catalyst insertion. High-throughput experimentation was necessary to determine the critical reagents (chiral auxiliary and catalyst) for both stages. The compound (**364**) was converted to compound (**365**), which was then treated with LDA to influence the acyl transfer and deprotonation. The resultant phenol was then reprotected to provide product (**366**). In compound (**367**), the two protective groups were swapped out for a single acetonide, which was subsequently introduced to the acrylate side chain through a Heck reaction (**368**). The *cis*-vinyl bromide (**369**) was produced by bromination and decarboxylative elimination. The production of the *cis*-vinyl boronate was achieved through the periodate breakdown of the pinacol ester after Pd-catalyzed Miyaura borylation with *bis*(pinacolato)-diboron (**370**). To achieve the enantioselective introduction of the cyclopropane, the TMTA boronate ester was first prepared. This was followed by Furukawa cyclopropanation, which produced (**371**) in a 95% yield [177,178]. After that, the enantiopurity was increased to >99% ee by substituting (+)-pinanediol for TMTA and using column chromatography to separate the diastereomers (**372**). Following the protecting group removal of the acetonide and pinanediol’s sequential hydrolysis, the free diacid (**373**) (QPX7728), which subsequently transformed into its crystalline disodium salt, was obtained. QPX7728 (**373**) (an ultrabroad-spectrum BLI) shows very good preclinical characteristics and considerable activities against *Acinetobacter*, *Pseudomonas*, and *Enterobacteriaceae* when coupled with other *β*-lactam antibiotics. It also inhibits both serine and metalloenzymes (Scheme 61) [70,179].

**Scheme 62:**
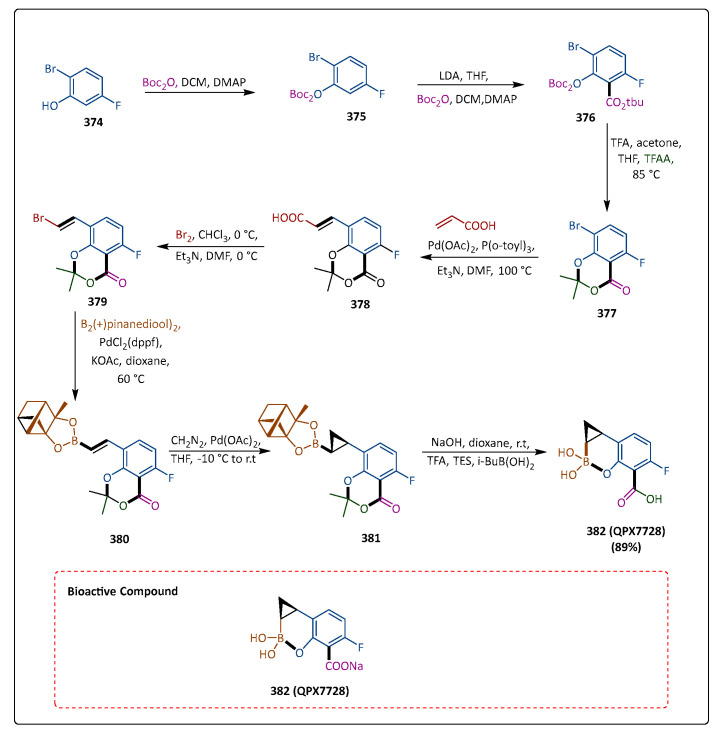
Synthesis of QPX7728.

Class B and class D enzymes are both remarkably broadly inhibited by QPX7728, which is mostly unaffected by either efflux or porin modifications. When combined with an antibiotic *β*-lactam, QPX7728 shows promise in dealing with some Gram-negative bacterial infections that are resistant to drugs and can be administered intravenously or orally. To produce a wider spectrum, increased efficacy (potential for both intravenous and oral uses) and decreased vulnerability to uptake resistance mechanisms, Hecker et al. discovered a serine and metallo-*β*-lactamase ultrabroad-spectrum inhibitor, cyclic boronic acid (QPX7728). Compound (**374**) is converted to tert-butylcarbonate (**375**); treatment with LDA effects deprotonation and acyl transfer, after which the resulting phenol is reprotected, giving (**376**). The resultant phenol is then reprotected, yielding compound (**377**). In compound (**377**), the two protective groups are swapped out for a single acetonide. This acetonide is then introduced to (**378**) via a Heck reaction that also introduces the acrylate side chain. To obtain *cis*-vinyl bromide (**379**), decarboxylative elimination (**378**) occurs after bromination. Palladium-catalyzed cyclopropanation with diazomethane yields (**380**) after *cis*-vinyl boronate (**381**) is produced by borylating tetrahydroxydiboron’s (+)-pinanediol ester. Following the HPLC separation of the two diastereomers, the pinanediol- and acetonide-protecting groups are hydrolyzed one after the other in a stepwise manner. The cyclopropane stereochemistry could be assigned thanks to the single-crystal X-ray structural identification of the diastereomer of (**381**), which leads to the potent final product (**382**), QPX7728, which exhibits strong action against most *β*-lactamase enzymes, including metalloenzymes, like NDM-1, as well as the serine enzymes of groups A and C. To facilitate the submission of an IND, QPX7728 has progressed to a late stage of preclinical development (Scheme 62) [70].

## 7. Summary

An era of antibiotic therapy that began with the discovery of penicillin is currently at risk because of the rapidly spreading problem of antibiotic resistance. Resistance against antibiotics of *β*-lactams is particularly concerning because they have been and continue to be the most-prescribed antibiotics globally. The synthesis of *β*-lactamases, which are enzymes that can hydrolyze *β*-lactams, is the primary mechanism of *β*-lactam resistance in Gram-negative bacteria (GNB). Some clinically concerning serine-*β*-lactamases (SBLs), like KPC-2 or OXA-48 (classes A and D, respectively), and/or metallo-*β*-lactamases (MBLs), like IMP-1, NDM-1, or VIM-1 (class B), do not spare even the strongest *β*-lactams, carbapenems.

Anticipating new antibiotics that target unexplored sites is challenging, but methods to combat *β*-lactam resistance, particularly by inhibiting *β*-lactamases, may help to maintain our present treatment options. With the advent of inhibitors of *β*-lactamases (BLIs), such as clavulanic acid and sulbactam, this tactic was put into practice more than 30 years ago, but it was not extensively studied until recently. Many new broad-spectrum second-generation *β*-lactamase inhibitors, belonging to classes A and C, have been discovered since 2012. Considerably noteworthy, the FDA approved the clinical use of avibactam and vaborbactam, while numerous congeners, including diazabicyclooctanes and other boron derivatives, including benzosiloxaboroles, are presently undergoing preclinical or clinical development.

**Figure 1:**
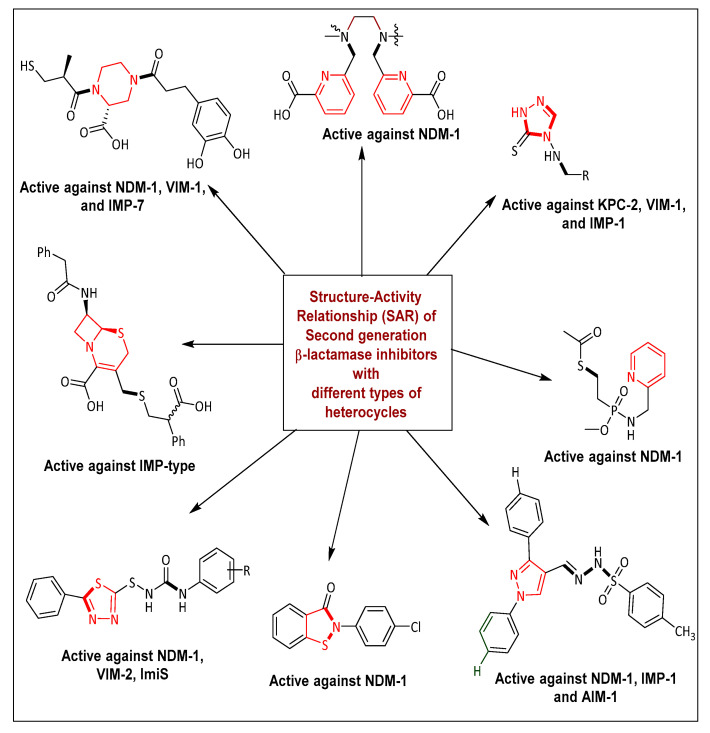
Structure–activity relationship (SAR) of different heterocyclic compounds.

These substances mostly have the ability to effectively block SBLs (classes A, C, and occasionally D), including carbapenemases; however, they typically have little effect on MBLs (class B). Parallel to this, ongoing efforts to generate effective MBL inhibitors have recently revealed promising compounds that can bind to the zinc atoms at the active sites of MBLs. These compounds include thiols or thiadiazoles, which include the antihypertensive drug aspergillomarasmine A, L-captopril, and other iminodiacetic acids, rhodanines, and their thienolate derivatives, or heteroaryl compounds, like ANT431 or ANT268. However, there is currently no MBL inhibitor approved for use in medicine. In Figure 1, The structure–activity relationship (SAR) defines the specific structural characteristic of heterocyclic compounds that are associated with its biological potential.

The discovery of inhibitors capable for concurrently blocking SBLs and MBLs becomes a relevant, albeit difficult, method as a result of an increase in bacterial isolates that produce two, or even three, separate carbapenemases belonging to different groups. With the exception of a few polyphenolic derivatives and tetrazoles with low actions, only heteroaryl phosphonates and derivatives of boronic acid have been demonstrated to efficiently inhibit both SBLs and MBLs. The first compounds to inhibit all kinds of b-lactamases were rigid cyclic analogs of vaborbactam, like VNRX-5133, which is presently undergoing phase 3 clinical trials. When it comes to several clinically significant MBLs (VIM-1 or VIM-2 and NDM-1) and SBLs (CMY-2, TEM-116, or OXA-10), VNRX-5133 demonstrates submicromolar inhibitory action. Novel thiol/boronic acid combinations, such as MS18, have been discovered recently and have shown intriguing dual inhibitory capabilities against both SBLs and MBLs. Although class B NDM-1 and OXA-48 are only mildly affected by MS18 and its congeners, they too have a rather wide spectrum. In Table 1, *β*-lactamase inhibitors are summarized.

Nowadays, the majority of the inhibitors reported in the literature are zinc-dependent. With the success of previous serine-*β*-lactamase inhibitor models and reports from modern researchers on zinc-independent MBL inhibitors, developing strong zinc-independent inhibitors could also be a promising strategy. Natural products have long been rich in possible MBL inhibitors. It is also interesting that a variety of computer techniques were employed in the development of inhibitors of MBL inhibitors. By employing these methods, inhibitor discovery efficiency can be greatly boosted. This has made a great deal of promising compounds available. The rapid growth of computers will lead to the creation and application of several techniques, including deep learning, in the upcoming years. Without a doubt, a key area for future MBL inhibitor discovery will be the integration of computer techniques with conventional inhibitor-screening methods.

## 8. Conclusions

The diversity of *β*-lactamases has increased 100-fold in the last 40 years. Developments in molecular diagnostics will help to identify *β*-lactamases, while chemical syntheses will target ever-emerging enzymes with the best *β*-lactamase inhibitors. The use of second-generation BLIs as a therapy contrary to microorganisms resistant to several antibiotics is essential. A combination of boronic acids, such as vaborbactam, and DBOs, particularly avibactam, helps *β*-lactams to resist bacteria that produce serine lactamases. Still, given the increasing predominance of bacteria resistant to BL-BLI combinations (avibactam–ceftazidime), it is unclear how long DBOs and boronic acids will serve as BLIs and keep their characteristics. For their catalytic activities, metallo-*β*-lactamases (MBLs) need metal ions. Even though combining inhibitors of *β*-lactamases with *β*-lactams is a well-known method to restore their efficacy, clinical practice currently lacks MBL inhibitors. Consequently, the production of inhibitors of MBLs is essential. Because of their rising global frequency in pertinent opportunistic Gram-negative bacteria, the public’s health is severely threatened by metallo-*β*-lactamases (MBLs). Several new *β*-lactamase inhibitor-containing anti-infective combinations, such as ceftazidime/avibactam and meropenem/vaborbactam, are available for purchase. The current trend among clinicians is to synthesize “universal” *β*-lactamase inhibitors because of limited time and a lack of sensitive diagnostics. Enhancing the antibacterial actions of *β*-lactam antibiotics using selective chelators with a compatible and modular linker can improve the biological efficacy of inhibitors and even bring them back to life. The recently developed second-generation *β*-lactamase inhibitors offer a potentially successful and highly promising strategy. Beta-lactamase inhibitors of the second generation play a variety of roles in drug development, structural biology, antibiotic discovery, bioconjugation chemistry, chemical biology, and biosensor development. They are useful instruments for addressing today’s healthcare issues and expanding our knowledge of biochemical processes because of their adaptable chemical characteristics and biological activities.

## 9. Future Perspectives

Second-generation *β*-lactamase inhibitors hold promise for combating antibiotic resistance. Researchers and healthcare providers can develop more effective treatments by enhancing stability, broadening the activity spectrum, and optimizing combination therapies. The integration of cutting-edge technologies and interdisciplinary approaches is crucial for future research. The roles of these inhibitors in chemistry are multifaceted, involving drug development, structural biology, antibiotic discovery, bioconjugation chemistry, biosensor development, and chemical biology. Continuous monitoring of resistance patterns and the development of novel chemical entities will ensure the continued efficacy of *β*-lactam antibiotics. Effective inhibitors must be developed in order to combat bacteria that produce metallo-*β*-lactamases. Even though there are a number of intriguing possibilities, further study is necessary to solve the problems with selectivity, stability, and broad-spectrum activity. The quest is still underway for novel inhibitors with improved pharmacokinetics, potency, and selectivity characteristics. Efforts include rational drug design, high-throughput screening of chemical libraries, and structure-based drug design utilizing molecular modeling and crystallography. An important step forward in the fight against antibiotic-resistant diseases would be the effective development of MBL inhibitors.

## Figures and Tables

**Table 1:** New *β*-lactamase inhibitors in clinical developments.

Sr. No.	Second-Generation *β*-Lactamase Inhibitors	Structure	Minimum Inhibitory Concentration (MIC) or IC_50_ (μg/mL or μM)	Yields
1	1,6-Diazabicyclo [3,2,1] Octanes	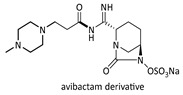	0.29 ± 2.34	[45]
2	1,6-Diazabicyclo [3,2,1] Octanes	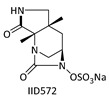	64	[57]
3	1,6-Diazabicyclo [3,2,1] Octanes	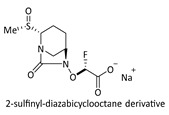	0.06	[64]
4	Boron-based inhibitors	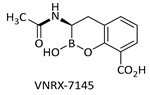	1	[71]
5	Boron-based inhibitors	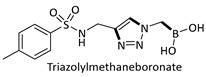	0.09 ± 0.01	[78]
6	Metallo-*β*-lactamase inhibitors	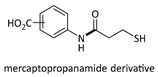	1.0 ± 0.4	[82]
7	Metallo-*β*-lactamase inhibitors	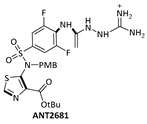	0.25	[109]
8	Metallo-*β*-lactamase inhibitors	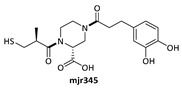	4.3 ± 0.44	[111]
9	Metallo-*β*-lactamase inhibitors	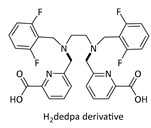	2 ± 0.25	[126]
10	Metallo-*β*-lactamase inhibitors	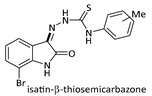	2.72	[134]
11	Metallo-*β*-lactamase inhibitors	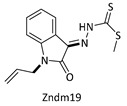	2.678	[135]
12	Metallo-*β*-lactamase inhibitors	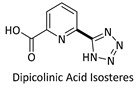	7.7 ± 0.6	[141]
13	Metallo-*β*-lactamase inhibitors	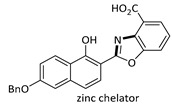	0.38	[156]
14	Dual nature of *β*-lactamase inhibitors	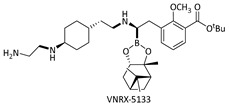	0.53 ± 0.008	[22]
15	Dual nature of *β*-lactamase inhibitors	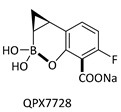	2 ± 8	[70]

## Abbreviations

Multidrug resistance (MDR); *β*-lactamase inhibitor (BLI); metallo-*β*-lactamase (MBL); diazabicyclooctane (DBO); diisopropylethylamine (DIPEA); cefotaxime (CTX); class A *β*-lactamase active against cefotaxime (CTX-M), first observed in Munich; extended-spectrum *β*-lactamase (ESBL); *N*,*N*,*N*′,*N*′-*t*etramethyl-O-(1H-benzotriazole-1-yl)uranium (HBTU); meropenem (MEM); class C chromosomally located cephalosporinase (AmpC); Klebsiella pneumoniae carbapenemases (KPC); New Delhi metallo-*β*-lactamase (NDM); *β*-lactamase named after the first patient, Temoneira (TEM); oxacillinase (OXA); penicillin-binding protein (PBP); phosphate-buffered saline (PBS); class A *β*-lactamase from Klebsiella pneumoniae was initially thought to be a “sulfhydryl variant” of the TEM enzyme (SHV); 3-nitrophenyl boronic acid (3-NPBA); tris[(1-benzyl-1H-1,2,3-triazole-4-yl)methyl]amine (TBTA); Verona integron-encoded metallo-*β*-lactamase (VIM); minimum inhibitory concentration (MIC); half-maximal inhibitory concentration (IC_50_).
